# Decellularized extracellular matrix as scaffold for cancer organoid cultures of colorectal peritoneal metastases

**DOI:** 10.1093/jmcb/mjac064

**Published:** 2022-12-02

**Authors:** Luca Varinelli, Marcello Guaglio, Silvia Brich, Susanna Zanutto, Antonino Belfiore, Federica Zanardi, Fabio Iannelli, Amanda Oldani, Elisa Costa, Matteo Chighizola, Ewelina Lorenc, Simone P Minardi, Stefano Fortuzzi, Martina Filugelli, Giovanna Garzone, Federica Pisati, Manuela Vecchi, Giancarlo Pruneri, Shigeki Kusamura, Dario Baratti, Laura Cattaneo, Dario Parazzoli, Alessandro Podestà, Massimo Milione, Marcello Deraco, Marco A Pierotti, Manuela Gariboldi

**Affiliations:** Department of Research, Fondazione IRCCS Istituto Nazionale dei Tumori, via G. Venezian 1, 20133 Milan, Italy; Peritoneal Surface Malignancies Unit, Colon and Rectal Surgery, Fondazione IRCCS Istituto Nazionale dei Tumori, via G. Venezian 1, 20133 Milan, Italy; Department of Pathology and Laboratory Medicine, Clinical Research lABoratory (CRAB), Fondazione IRCCS Istituto Nazionale dei Tumori, via G. Venezian 1, 20133 Milan, Italy; Department of Research, Fondazione IRCCS Istituto Nazionale dei Tumori, via G. Venezian 1, 20133 Milan, Italy; Department of Pathology and Laboratory Medicine, Clinical Research lABoratory (CRAB), Fondazione IRCCS Istituto Nazionale dei Tumori, via G. Venezian 1, 20133 Milan, Italy; Bioinformatics Core Unit, IFOM ETS, The AIRC Institute of Molecular Oncology, via Adamello 16, 20139 Milan, Italy; Bioinformatics Core Unit, IFOM ETS, The AIRC Institute of Molecular Oncology, via Adamello 16, 20139 Milan, Italy; Imaging Technological Development Unit, IFOM ETS, The AIRC Institute of Molecular Oncology, via Adamello 16, 20139 Milan, Italy; Imaging Technological Development Unit, IFOM ETS, The AIRC Institute of Molecular Oncology, via Adamello 16, 20139 Milan, Italy; C.I.Ma.I.Na. and Dipartimento di Fisica ‘Aldo Pontremoli’, Università degli Studi di Milano, via G. Celoria 16, 20133 Milan, Italy; C.I.Ma.I.Na. and Dipartimento di Fisica ‘Aldo Pontremoli’, Università degli Studi di Milano, via G. Celoria 16, 20133 Milan, Italy; Cogentech Ltd Benefit Corporation with a Sole Shareholder, via Adamello 16, 20139 Milan, Italy; Cogentech Ltd Benefit Corporation with a Sole Shareholder, via Adamello 16, 20139 Milan, Italy; Pathology and Laboratory Medicine Department, Fondazione IRCCS Istituto Nazionale dei Tumori, via G. Venezian 1, 20133 Milan, Italy; Pathology and Laboratory Medicine Department, Fondazione IRCCS Istituto Nazionale dei Tumori, via G. Venezian 1, 20133 Milan, Italy; Cogentech Ltd Benefit Corporation with a Sole Shareholder, via Adamello 16, 20139 Milan, Italy; Cogentech Ltd Benefit Corporation with a Sole Shareholder, via Adamello 16, 20139 Milan, Italy; Department of Pathology and Laboratory Medicine, Clinical Research lABoratory (CRAB), Fondazione IRCCS Istituto Nazionale dei Tumori, via G. Venezian 1, 20133 Milan, Italy; Peritoneal Surface Malignancies Unit, Colon and Rectal Surgery, Fondazione IRCCS Istituto Nazionale dei Tumori, via G. Venezian 1, 20133 Milan, Italy; Peritoneal Surface Malignancies Unit, Colon and Rectal Surgery, Fondazione IRCCS Istituto Nazionale dei Tumori, via G. Venezian 1, 20133 Milan, Italy; Pathology and Laboratory Medicine Department, Fondazione IRCCS Istituto Nazionale dei Tumori, via G. Venezian 1, 20133 Milan, Italy; Imaging Technological Development Unit, IFOM ETS, The AIRC Institute of Molecular Oncology, via Adamello 16, 20139 Milan, Italy; C.I.Ma.I.Na. and Dipartimento di Fisica ‘Aldo Pontremoli’, Università degli Studi di Milano, via G. Celoria 16, 20133 Milan, Italy; Pathology and Laboratory Medicine Department, Fondazione IRCCS Istituto Nazionale dei Tumori, via G. Venezian 1, 20133 Milan, Italy; Peritoneal Surface Malignancies Unit, Colon and Rectal Surgery, Fondazione IRCCS Istituto Nazionale dei Tumori, via G. Venezian 1, 20133 Milan, Italy; Cogentech Ltd Benefit Corporation with a Sole Shareholder, via Adamello 16, 20139 Milan, Italy; Department of Research, Fondazione IRCCS Istituto Nazionale dei Tumori, via G. Venezian 1, 20133 Milan, Italy

**Keywords:** colorectal cancer, peritoneal metastasis, organoids, extracellular matrix (ECM), decellularized extracellular matrix, engineered disease model, ECM stiffness

## Abstract

Peritoneal metastases (PM) from colorectal cancer (CRC) are associated with poor survival. The extracellular matrix (ECM) plays a fundamental role in modulating the homing of CRC metastases to the peritoneum. The mechanisms underlying the interactions between metastatic cells and the ECM, however, remain poorly understood, and the number of *in vitro* models available for the study of the peritoneal metastatic process is limited. Here, we show that decellularized ECM of the peritoneal cavity allows the growth of organoids obtained from PM, favoring the development of three-dimensional (3D) nodules that maintain the characteristics of *in vivo* PM. Organoids preferentially grow on scaffolds obtained from neoplastic peritoneum, which are characterized by greater stiffness than normal scaffolds. A gene expression analysis of organoids grown on different substrates reflected faithfully the clinical and biological characteristics of the organoids. An impact of the ECM on the response to standard chemotherapy treatment for PM was also observed. The *ex vivo* 3D model, obtained by combining patient-derived decellularized ECM with organoids to mimic the metastatic niche, could be an innovative tool to develop new therapeutic strategies in a biologically relevant context to personalize treatments.

## Introduction

The peritoneum is the second most common site of metastasis for colorectal cancer (CRC) after the liver (Herszeny and Tulassay, 2010). In the past, peritoneal metastases (PM) were considered a terminal condition, amenable only to palliative treatments. The advent of cytoreductive surgery (CRS) and hyperthermic intraperitoneal chemotherapy (HIPEC) in the 1990s have allowed some patients with PM to achieve long-term survival, pushing the median overall survival from 16 to 51 months (Baratti et al., 2015). However, ∼70% of treated patients still experience peritoneal relapse (Yan et al., 2006). The development of preclinical cellular models that faithfully recapitulate PM pathology, therefore, is crucial for the identification of more effective therapeutic strategies.

The peritoneal metastatic cascade consists of a series of steps that begin with cell detachment from the primary tumor (Jayne, 2007). Fine-tuned interactions between biochemical factors and biomechanical events, such as remodeling of the extracellular matrix (ECM), govern the cascade and allow the formation of the metastatic niche (Ksiazek et al., 2010; Lemoine et al., 2016; Peinado et al., 2017; Mikula-Pietrasik et al., 2018). The metastatic niche facilitates organotrophic metastasis through the direct promotion of cancer stem cell survival, exploiting a tissue-specific microenvironment that is more suitable for the attachment of exfoliated neoplastic cells (Lemoine et al., 2016). The biology behind these processes, however, is poorly understood due to the lack of organ-specific experimental models.

Most of the current data on metastatic spread have been obtained using cancer cell lines or patient-derived xenograft models, which do not fully reflect the physiopathology of their tumor of origin (Drost and Clevers, 2018). Tumor-derived organoids (TDO) are an intermediate model between cell lines and xenografts. They grow three-dimensionally and retain cell–cell and cell–matrix interactions, which more closely reflect the characteristics of the original tumor. Importantly, organoids can be established in a short time and are easy to manipulate (Fujii et al., 2016), and they retain the genetic status of the original tissues and can be used to identify new therapeutic targets (Bozzi et al., 2017).

The possibility to isolate natural decellularized ECMs (dECMs) while preserving both three-dimensional (3D) tissue architecture and biochemical properties, enabling the development of more physiological cancer models (Genovese et al., 2014; Chen et al., 2016), prompted us to develop a tissue-engineered PM model for *in vitro* studies. Our model is based on seeding PM-derived organoids onto decellularized peritoneum-derived ECMs. By being able to characterize the biochemical and biophysical properties of both organoids and ECM, the developed PM model allowed us to study the complex interactions between the ECM and neoplastic cells, gaining new insights into PM biology and the mechanisms underlying cell–microenvironment interaction in this system.

## Results

### Development of PM-derived organoids

Six organoid cultures (C1–C6) were developed from PM, following established protocols (Fujii et al., 2015, 2016), as detailed in the Materials and methods section. The main characteristics of the patients from which the organoids were derived are summarized in Supplementary Table S1. In line with previous works on organoids from advanced CRC (Fujii et al., 2015, 2016), the growth of the organoids required the supplementation of minimal niche factors. Organoids carrying mutations in the *KRAS* gene mainly required noggin-1 supplementation, while organoids carrying *FGR1* amplification grew in the medium with epidermal growth factor (EGF). *BRAF*-mutant TDO grew in the medium with minimal requirements for niche factors. These results highlight how different mutational profiles drive specific niche factor requirements (Supplementary Table S2; Fujii et al., 2016).

### PM-derived organoid characterization

**Figure 1:**
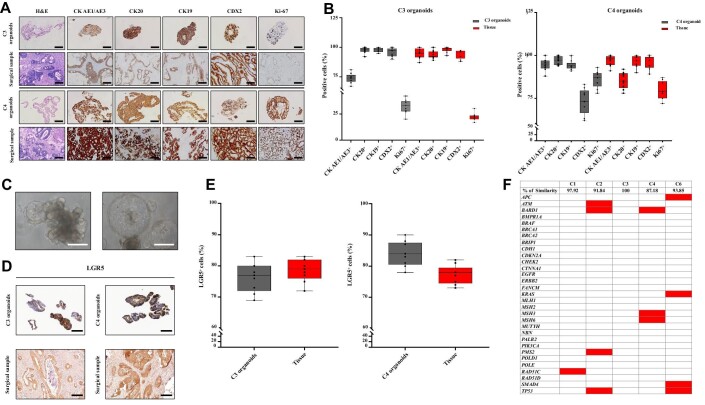
Establishment and characterization of human PM-derived organoids. (**A**) Comparative histochemistry (HC) and IHC analysis of PM-derived organoids and their tissue of origin. Scale bar, 100 µm. Surgical samples and the derived C3 and C4 organoids (passage numbers: P10 and P14, respectively) were developed from patients S16-8598 and S17-3963 who underwent CRS–HIPEC for PM (Supplementary Table S1). (**B**) Quantitative analysis for the expression of CRC markers and Ki-67 in C3 and C4 organoids versus their corresponding tumor of origin. Positive cells were measured as the percentage of CK AE1/AE3^+^, CK20^+^, CK19^+^, CDX2^+^, and Ki-67^+^ cells on the total number of cells present in regions free of dead cells. Three high-magnification (40×) fields per experiment were counted. Data are shown as median ± standard deviation (SD) of CK AE1/AE3^+^, CK20^+^, CK19^+^, CDX2^+^, and Ki-67^+^ cells present in each FFPE sample. Cells were counted using Qupath software. The experiment was performed in triplicate. (**C**) Micrographs showing a glandular-like branched (left) and a spherical-like cohesive (right) organoid. Scale bar, 100 µm. (**D**) IHC analysis of organoids (top) and their tissue of origin (bottom), using LGR5 immunostaining. Scale bar, 100 µm. (**E**) Quantitative counts of the percentage of LGR5^+^ cells in C3 and C4 organoids versus their corresponding tumor of origin. Positive cells were measured as the percentage of LGR5^+^ cells on the total number of cells present in regions free of dead cells. Three high-magnification (40×) fields per experiment were counted, and data are presented as median ± SD of LGR5^+^ cells present in each FFPE sample. Cells were counted using Qupath software. The experiment was performed in triplicate. (**F**) Summary of cancer-related genes with acquired mutations in TDO with respect to their tumor of origin (red boxes). The percentage of similarity was reported. Passage numbers of the organoid lines were: C1, P11; C2, P13; C3, P10; C4, P14; C6, P10.

PM-derived organoids retained the main characteristics of their tissues of origin, expressing the colorectal-specific markers CK AE1/AE3, CK20, CK19, and CDX2 in the same percentage of cells as the tissue from which they originated (Figure 1A and B; Supplementary Figure S1A, C, and E). The TDO exhibited the typical glandular features observed in the corresponding surgical sample, including signet ring cells, nest-like growth pattern, nuclear atypia, cuboidal nuclear morphology, and pleomorphism (Figure 1A and C).

All organoids and corresponding clinical samples were positive for LGR5, a well-established stem cell marker for the colonic niche (Figure 1D; Supplementary Figure S1B and D; Bozzi et al., 2017). The percentage of LGR5^+^ cells was similar to that of their tissue of origin (Figure 1E; Supplementary Figure S1F). The organoids were also Ki-67^+^, suggesting that they underwent active proliferation (Figure 1A; Supplementary Figure S1A and C). Moreover, the TDO mutational profiles were very similar to those of the tumor of origin (Figure 1F; Supplementary Figure S1G).

### Development of 3D-dECMs

**Figure 2:**
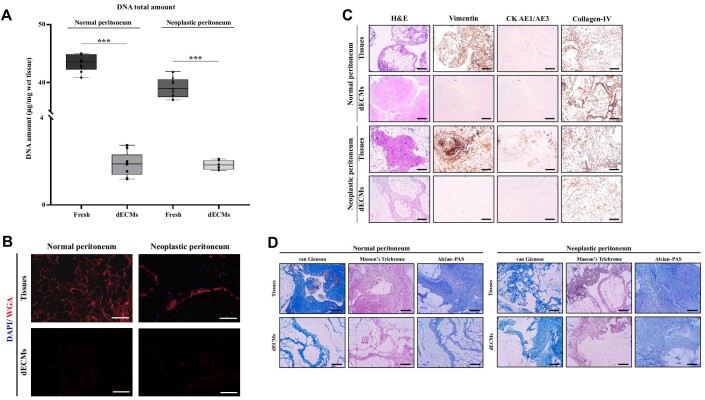
Establishment of 3D-dECM scaffolds from peritoneal cavity. (**A**) DNA quantification of normal and neoplastic peritoneal tissue samples before (fresh) and after the decellularization treatment (dECM). Student's *t-*test (***P < 0.001). (**B**) IF analysis of normal and neoplastic peritoneal samples before and after decellularization using the WGA (red) staining. The samples were counterstained with DAPI (blue). Scale bar, 100 µm. (**C**) IHC analysis of fresh peritoneum-derived tissues and the corresponding decellularized samples using H&E staining and vimentin, pan-Cytokeratin, and collagen-IV immunostaining. Scale bar, 200 µm. (**D**) Van Gienson, Masson's Trichrome, and Alcian–PAS staining on fresh and decellularized peritoneum-derived samples. Scale bar, 200 µm.

3D-dECMs from PM and normal peritoneum were generated following the protocol described by Genovese et al. (2014). DNA quantification showed almost complete DNA depletion after the decellularization procedure (***P < 0.001; Figure 2A; Supplementary Figure S2A). Fluorescence analysis of formalin-fixed paraffin-embedded (FFPE) sections highlighted the complete removal of the nuclei and lipids from the cellular membranes (Figure 2B). Immunohistochemistry (IHC) analysis showed the loss of cytokeratins and vimentin, indicating the absence of epithelial and mesenchymal cells, respectively (Figure 2C). IHC analysis also showed the retained distribution of collagen-IV, which is the main structural protein (Figure 2C). hematoxylin and eosin (H&E), van Gienson, Masson trichrome, and Alcian–PAS staining revealed the maintenance of the architectural structure of the corresponding non-decellularized samples (Figure 2C and D). Finally, lyophilyzed 3D-dECMs were added to the culture media and no differences were observed in cell viability after 72 h of growth, indicating that the decellularization procedure has no cytotoxic effects (Supplementary Figure S2B).

### 3D-dECMs: morphological features and mechanical properties

**Figure 3:**
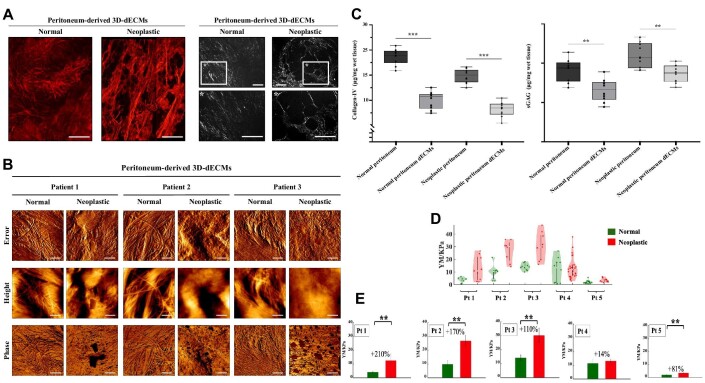
Morphological and mechanical properties of PM-derived 3D-dECMs. (**A**) Confocal and polarized light microscopy analysis of peritoneum-derived 3D-dECM samples. Confocal images (left panel) were obtained by scanning different areas of the samples (area ∼1 mm^2^), which were then assembled into a single mosaic figure. Scale bar, 200 µm (confocal images) and 50 µm (light microscopy images). (**B**) Topography analysis of peritoneum-derived 3D-dECMs. Phase, height, and peak force error images of both normal and neoplastic decellularized matrices are shown. Scale bar, 1 µm. (**C**) Quantification of collagen-IV and sGAG on fresh and decellularized peritoneal tissues. Student's *t-*test (***P < 0.001 and **P < 0.01). (**D**) Distribution of the YM values obtained for each patient and condition (normal and neoplastic). Violin-plots: each dot represents the median YM value extracted from a single measurement with Point and Shot made by ∼225 force curves. Pt, patient; KPa, kilo Pascal. (**E**) Statistical analysis results of the YM value for each patient and condition tested. The bars and error bars represent mean of the median YM values and effective SD of the mean. The percentages represent the relative stiffening of the neoplastic ECM. Student's *t-*test (**P < 0.01).

Confocal reflection, polarized light microscopy, and atomic force microscopy (AFM) techniques were used to characterize the biophysical properties of patients’ peritoneal tissues. Confocal reflection and polarized light investigations showed that the differences between normal and neoplastic tissues obtained from three different PM patients were related to both tissue texture and single collagen fiber distribution and integrity (Figure 3A). The normal 3D-dECMs showed a higher density and clusters of collagen fibers, with a random relative orientation; in the neoplastic 3D-dECMs, the collagen structure appears more irregular and porous, although a tendency toward alignment and clustering of fibers on a larger scale can be observed. AFM topographical analysis revealed an asymmetric distribution of collagen on a micrometric scale: matrices derived from normal tissues are organized in groups of very thin, intersecting fibers (with diameters <50 nm), characterized by a variety of orientations, while matrices derived from neoplastic tissues exhibit a more coiled and disarranged surface pattern (Figure 3B).

At odds with the case of ECM derived from healthy and CRC-affected tissues (Nebuloni et al., 2016), we do not observe a clear tendency for collagen fibers to form aligned bundles in the neoplastic state, although at this small scale, the neoplastic matrix appears structurally more compact than the normal ECM. The increased anisotropy of the ECM structure reported by Nebuloni et al. (2016) in the case of CRC is more evident in the confocal optical image of the neoplastic sample (Figure 3A). Collagen was less abundant in the neoplastic tissue than in normal tissue, but the latter showed higher levels of glycosaminoglycans (GAGs). Both collagen and GAG levels decreased in decellularized samples (∼20% loss in 3D-dECMs; ***P < 0.001 and **P < 0.01, Figure 3C).

AFM nanomechanical analysis (details in Supplementary Materials and methods) showed that the neoplastic 3D-dECMs had a wider distribution of Young's modulus (YM) values and were markedly stiffer than normal 3D-dECMs (Figure 3D). In addition, the YM distributions were considerably scattered and there was a significant overlap between the two conditions. These results indicate that the ECM is a complex system that remains locally heterogeneous on the scale of several typical cellular lengths, i.e. 10–100 µm, because the transition from the normal to the neoplastic condition, in terms of change in stiffness and structural organization, is not uniform across the whole macroscopic tissue region. Figure 3E shows YM values measured at the different sites and conditions: the stiffening is statistically significant for four out of five patients.

### Decellularized scaffolds sustain PM-derived organoid growth

**Figure 4:**
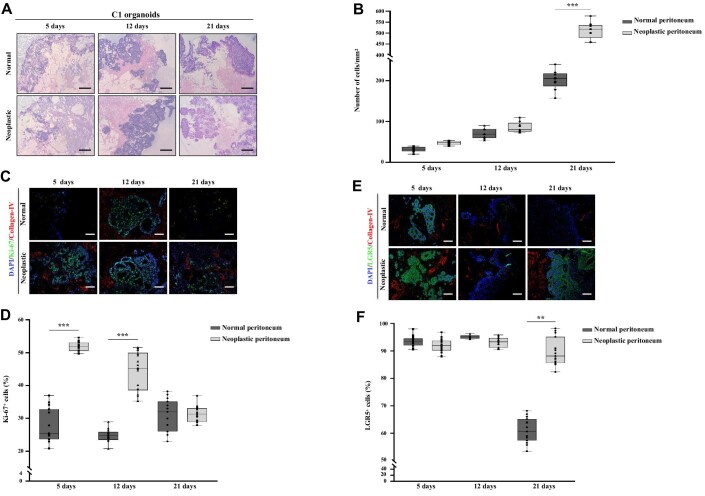
Peritoneum-derived 3D-dECM scaffolds support colonization, infiltration, and proliferation of PM-derived organoids, maintaining the stem cell pool. (**A**) H&E staining of decellularized matrices derived from normal (top) or neoplastic (bottom) peritoneum repopulated with PM-derived organoids (C1). Scale bar, 50 µm. The repopulation experiments were performed in triplicate. (**B**) Number of cells from PM-derived organoids grown on normal and neoplastic 3D-dECMs after 5, 12, and 21 days. Data are presented as median ± SD of three fields per experiment, counted using Qupath software. One-way ANOVA (***P < 0.001). (**C**) IF analysis of 3D-dECMs derived from normal (top) and neoplastic (bottom) peritoneum repopulated with organoids (C1) using Ki-67 (green) and collagen-IV (red) antibodies. The samples were counterstained with DAPI (blue). Scale bar, 20 µm. (**D**) Proliferation rate of organoids measured as the percentage of Ki-67^+^ cells present in fields devoid of dead cells. Data are presented as median ± SD of five fields per experiment (40× magnification), counted using Qpath software. One-way ANOVA (***P < 0.001). (**E**) IF analysis of 3D decellularized matrices derived from normal (top) and neoplastic (bottom) peritoneum repopulated with organoids (C1) using LGR5 (green) and collagen-IV (red) antibodies. The samples were counterstained with DAPI (blue). Scale bar, 20 µm. (**F**) Amount of stem cells in organoids, measured as the percentage of LGR5^+^ cells present in fields devoid of dead cells. Data are presented as median ± SD of five fields per experiment (40× magnification), counted using Qupath software. One-way ANOVA (**P < 0.01).

To develop an *in vitro* model of PM disease, 3D-dECMs were repopulated with C1, C2, and C3 TDO, with mutational profiles resembling the most common gene alterations in CRC (C1 was *KRAS*-mutant, C2 was *BRAF*-mutant, while C3 had amplified *FGFR1*). In line with literature data showing that colonization of decellularized matrices takes 8–12 days, TDO were grown on the matrices for 5, 12, and 21 days (Genovese et al., 2014; Chen et al., 2016; Weijing et al., 2021). Five days after seeding, C1 organoids were localized along the perimeter of the 3D-dECMs generated from the normal peritoneum (Figure 4A). A similar distribution was observed at 12 and 21 days after seeding, with a slight increase in the number of cells in the stromal region. In the 3D-dECMs generated from the neoplastic peritoneum, instead, C1 TDO were distributed throughout the matrix at 5 days after seeding, and colonization was evident with high stromal infiltration at Day 21 (Figure 4A; Supplementary Figure S3A and B). After 5 days, TDO maintained their spheroid shape on both scaffolds; at Day 12, single cells began to be observed, especially on the normal matrix, and their number increased significantly at Day 21 (Supplementary Figure S3C). After 21 days, TDO grown on neoplastic 3D-dECMs were able to infiltrate and colonize the ECM much better than TDO grown on normal 3D-dECMs (***P < 0.001; Figure 4B; Supplementary Figure S3D and E). The cell density of C1, C2, and C3 TDO grown on neoplastic 3D-dECMs was 520, 960, and 980 cells/mm^2^, respectively, and decreased to 200, 470, and 500 cells/mm^2^ when TDO were cultured on normal 3D-dECMs. The morphological characteristics and the efficiency of TDO infiltration were correlated with the grade and differentiation state of their tumors of origin. Organoids derived from grade III/G2 metastatic lesions with moderate undifferentiation and low invasive capacity (C1) infiltrated into the 3D-dECMs as single cells, while organoids from poorly undifferentiated grade IV/G3 tumors with *BRAF* mutation (C2) and *FGFR1* amplification (C3) infiltrated into the 3D-dECMs maintaining the spheroid shape (Figure 4A and B; Supplementary Figure S3A–E). Finally, we performed cleaved CASPASE3 (cCASPASE3) staining to assess cell death of TDO grown on the different 3D-dECMs, observing that cCASPASE3 expression was higher in TDO grown on normal peritoneum scaffolds (Supplementary Figure S3F).

### 3D decellularized scaffolds support the proliferation of organoids

Results showed that 3D-dECMs generated from neoplastic peritoneum had a significantly higher percentage of Ki-67^+^ cells at Days 5 and 12 (***P < 0.001) after seeding, indicating that these organoids underwent a faster growth (Figure 4C and D; Supplementary Figure S3G–L). The neoplastic peritoneum appears to be more favorable for organoid growth, partly because factors promoting TDO growth previously released by resident cells were not removed by the decellularization methods. No differences were observed at Day 21 after seeding. Most likely, after 21 days of growth, the cells on the neoplastic matrix are confluent and the stem cell pool stops growing.

LGR5^+^ staining showed that the stem cell pool was maintained on both normal and neoplastic 3D-dECMs at 5 and 12 days after seeding (Figure 4E and F; Supplementary Figure S3G–L). At Day 21, the stem cell pool was significantly lower on 3D-dECMs generated from normal peritoneum than on 3D-dECMs generated from neoplastic peritoneum. Most likely, the cells are confluent and the stem cell pool grown on the neoplastic matrix has an environment that favors the maintenance of its phenotype, as demonstrated by transcriptomics analyses (see below and Supplementary Figure S5C).

### Stiffness of 3D-dECMs does not activate YAP/TAZ proteins

**Figure 5:**
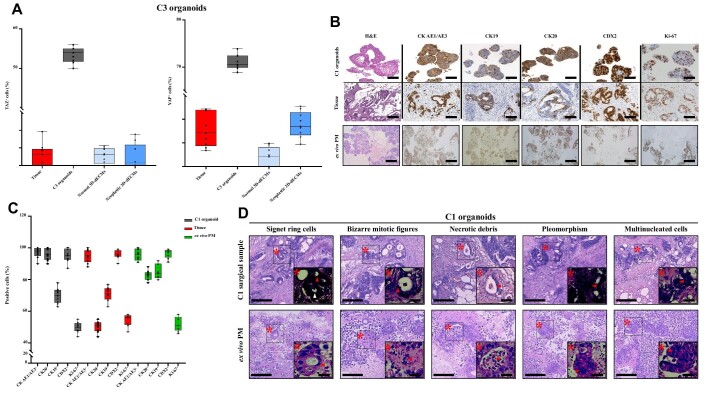
*Ex vivo* engineered PM lesions are comparable to PMs *in vivo*. (**A**) Percentage of YAP^+^ and TAZ^+^ cells in PM-derived TDO (C3). Data are presented as median ± SD of three fields per experiment (40× magnification), counted using Qupath software. (**B**) Comparative HC and IHC images of organoids (C1) versus their corresponding tumor of origin and the *ex vivo* engineered PM lesion. Samples were analyzed for the expression of CRC-specific markers and Ki-67. Scale bar, 100 µm. Images in the first two lanes were previously published (Bozzi et al., 2017). (**C**) Quantitative counts of the percentage of CRC marker-positive and Ki-67^+^ cells in C1 organoids versus their corresponding tumor of origin and the *ex vivo* engineered PM lesion. Data are presented as median ± SD of three fields per experiment, counted using Qupath software. One-way ANOVA did not show differences among the three groups. (**D**) HC comparison of PM surgical sample and neoplastic peritoneum-derived 3D-dECMs repopulated with PM-derived organoids (C1). Asterisks and arrows indicate the main morphological features. Scale bar, 20 µm.

The expression of Yes-associated protein (YAP) and Tafazzin (TAZ) was investigated on TDO grown on different substrates. IHC analyses showed that YAP and TAZ were expressed in all TDO grown in Matrigel and localized in the nucleus and in the cytoplasm, respectively (Supplementary Figure S4A–C). TDO were positive for YAP/TAZ expression. For example, C3 showed 55% of cells positive for TAZ and 75% of cells positive for YAP (Figure 5A; Supplementary Figure S4A–C). In contrast, YAP and TAZ were not expressed in TDO grown on normal and neoplastic 3D-dECMs, with cells positive for YAP and TAZ always <5% (Figure 5A–C; Supplementary Figure S4A–C), indicating that the peritoneum-derived matrix is able to modulate and block their expression levels. Both proteins were expressed only in C1 (Supplementary Figure S4A–C), with YAP mainly located in the nucleus and TAZ in the cytoplasm.

### Ex vivo engineered PM lesions reproduce patient PM

IHC analysis of the TDO using colorectal markers showed that repopulated 3D-dECMs retain the main characteristics and the morphology of their tumor of origin (Figure 5B; Supplementary Figure S4D and F), expressing the colorectal markers in almost the same percentage of cells as their tissue of origin (Figure 5B and C; Supplementary Figure S4H and I), except for CK20 and CK19 more expressed in the *ex vivo* model, probably because decellularized matrices stimulate their expression. However, ANOVA analysis among the three groups showed no significant variations. The *ex vivo* PM lesions had the typical histological features observed in PM patients, such as (i) signet ring cells, (ii) bizarre mitotic figures, (iii) necrotic debris, (iv) pleomorphic cell size and shape, and (v) multinucleated cells (Figure 5D; Supplementary Figure S4E and G). Signet ring cells have been reported in another *ex vivo* model system (Tian et al., 2018), highlighting that our model of PM is highly representative of *in vivo* lesions.

### Gene expression analysis of engineered PM lesions

**Figure 6:**
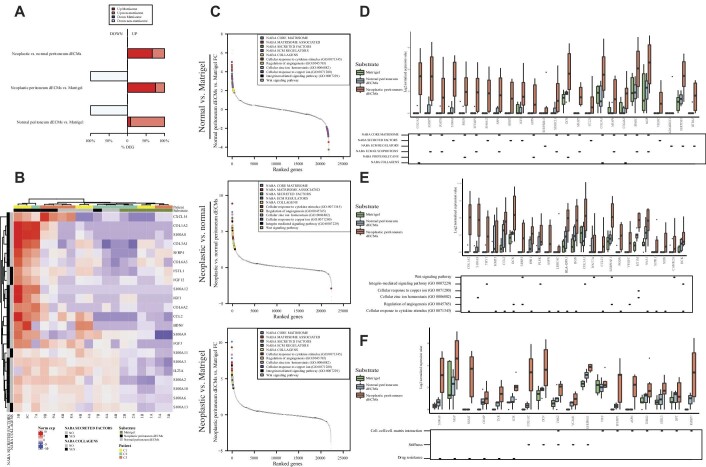
Gene expression analysis of engineered PM lesions. (**A**) Percentages of upregulated and downregulated genes belonging to the Matrisome dataset. (**B**) Unsupervised hierarchical clustering of the organoids according to the expression of the top DEGs included in Naba Secreted Factors and in Naba Collagens categories. (**C**) FCs of genes belonging to the indicated gene sets among the top 100 deregulated genes. Gene ranks for relative FC are shown on the x-axis and the logFCs on the y-axis. (**D**) Box plots showing the expression of genes selected for their involvement in the indicated processes of the Naba Matrisome datasets. Median and interquartile range are displayed as horizontal lines. Black squares in the bottom panel indicate which category the genes belong to. (**E**) Expression of genes selected for their involvement in the indicated processes of GO biological process and KEGG databases. Median and interquartile range are displayed as horizontal lines. Black squares in the bottom panel indicate which category the genes belong to. (**F**) Expression of genes selected for their involvement in the indicated processes, using a selection of genes related to the following biological processes: cell–cell/cell–matrix interactions, extracellular matrix stiffness, and drug resistance. Median and interquartile range are displayed as horizontal lines. Black squares in the bottom panel indicate which category the genes belong to.

RNA sequencing (RNA-seq) analysis of the TDO (Figure 6) revealed differences between 3D-dECMs and Matrigel substrates: 327 differentially expressed genes (DEGs) were identified in TDO grown on normal 3D-dECMs and 144 DEGs were identified in TDO grown on neoplastic 3D-dECMs, compared with the TDO grown in Matrigel (|fold change (FC)| > 1.5 and adjusted *P*-value <0.05).

The most represented biological processes include cell–cell/cell–matrix interactions, organoid behavior and interactions with the ECM, angiogenesis, metal ion homeostasis, and response to external stimuli. 3D-dECMs deregulated many genes critical for the 3D architecture/organization of the ECM. Many of the upregulated genes identified in TDO grown on neoplastic 3D-dECMs were assigned to the Matrisome database (67% for neoplastic vs. normal 3D-dECMs, and 25% for neoplastic 3D-dECMs vs. Matrigel). Only 8% of the upregulated genes identified in TDO grown on normal 3D-dECMs compared with TDO grown in Matrigel belonged to the Matrisome database (Figure 6A). Unsupervised hierarchical clustering of TDO showed a separation between TDO grown on neoplastic 3D-dECMs and TDO grown on normal 3D-dECMs and in Matrigel substrates (Figure 6B; Supplementary Figure S5A–C). TDO grown on normal and neoplastic 3D-dECMs exhibited an over-representation of DEGs involved in ECM composition, regulation, and modulation (Figure 6C). Growth on 3D-dECMs also promotes the expression of genes involved in the regulation of angiogenic processes, response to cytokine stimuli, the integrin pathway, and copper and zinc metabolism. The same categories were found for both normal and neoplastic 3D-dECMs (Supplementary Figure S5C and D).

Using the Matrisome database, we identified genes related to the ECM core composition and ECM interactors/regulators through secretion of specific factors, all of which were higher in 3D-dECMs derived from neoplastic tissue (Figure 6D; Supplementary Figure S5E–H). The 3D-dECMs had high expression of genes involved in stem cell pathways, cellular response to cytokines, zinc and copper metabolism, integrin pathway, and regulation of angiogenesis (Figure 6E; Supplementary Figure S5E–H). Similar results were found for genes involved in cell–cell/cell–matrix interactions (Figure 6F). Finally, gene set enrichment analysis showed no differences in genes from the pathways known to be regulated by YAP/TAZ (Supplementary Figure S5M).

The differences observed with transcriptomic data were validated by quantitative real-time polymerase chain reaction of some representative genes on C1, C2, and C3 TDO grown in Matrigel and on normal and neoplastic 3D-dECMs. The tissue of origin of the six TDO was also analyzed. The trends observed by RNA-seq were confirmed and all the genes analyzed were also expressed in the tissue of origin (Supplementary Figure S5I and L).

### 3D-dECMs decrease the efficacy of HIPEC treatments

**Figure 7:**
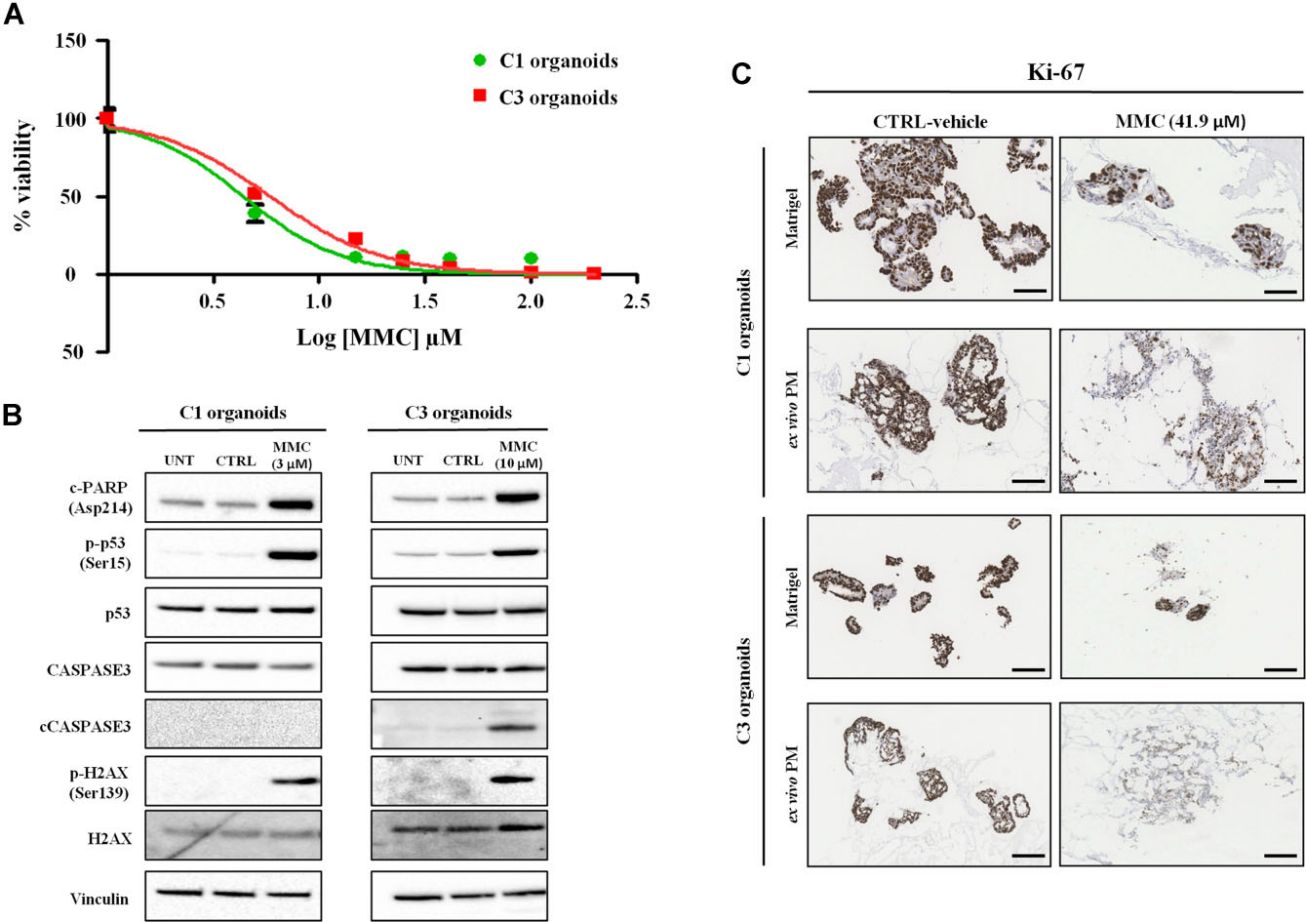
3D-dECM scaffolds decrease the efficacy of HIPEC treatments. (**A**) Dose–response curve of C1 and C3 organoids cultured in Matrigel and treated with MMC at different concentrations at 42.5°C for 1 h. (**B**) Immunoblots of C1 and C3 organoids treated with MMC at 3 µM and 10 µM, respectively. Vinculin was used as loading control. (**C**) IHC analysis of C1 and C3 organoids cultured in Matrigel and on neoplastic peritoneum-derived 3D-dECMs, after *in vitro* HIPEC treatments, using Ki-67 immunostaining. Scale bar, 50 µm.

The TDO analyzed had different IC_50_ values: C3 TDO were the most sensitive and C2 the least one to both mitomycin-c (MMC) and oxaliplatin (OXA) (Figure 7A; Supplementary Figure S6C and E). At these values, MMC treatment induced DNA damage and apoptosis in all TDO, as shown by phosphorylation of p53 (Ser15) and H2AX (Ser139) (Figure 7B; Supplementary Figure S6D) and cleavage of PARP and CASPASE3 (Figure 7B; Supplementary Figure S6D). OXA treatment also induced DNA damage and apoptosis in all TDO, although C2 TDO showed only PARP cleavage (Supplementary Figure S6G).

**Figure 8:**
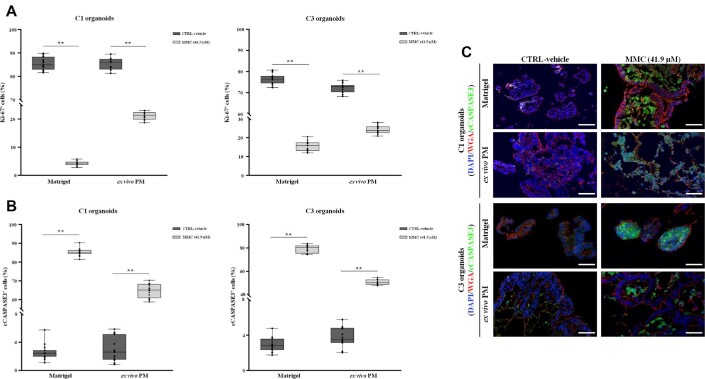
3D-dECMs reduce HIPEC-induced apoptosis in TDO. (**A**) Proliferation rate of PM-derived organoids (C1, left panel; C3, right panel) measured as the percentage of Ki-67^+^ cells present in fields free of dead cells. Data are presented as median ± SD of five fields per experiment (40× magnification), counted using Qupath software. One-way ANOVA (**P < 0.01). (**B**) Percentage of apoptotic organoids (C1, left panel; C3, right panel) measured as the percentage of cCASPASE3^+^ cells present in selected fields. Data are presented as median ± SD of five fields per experiment (40× magnification), counted using Qupath software. One-way ANOVA (**P < 0.01). (**C**) IF analysis of C1 and C3 organoids cultured in Matrigel and on neoplastic peritoneum-derived 3D-dECMs, after HIPEC treatments, using cCASPASE3 antibody (green). The samples were counterstained with WGA (red) and DAPI (blue). Scale bar, 20 µm.

To evaluate the contribution of the ECM to treatment response, we simulated treatment with HIPEC on *ex vivo* engineered micrometastases. C1, C2, and C3 TDO were grown on neoplastic 3D-dECMs for 12 days, a time considered sufficient for the matrix surface colonization (Weijing et al., 2021), and treated with MMC and OXA. Treatment with MMC induced cell rupture in C1 and C3 organoids (Supplementary Figure S6A). Ki-67 expression was lower in the treated group, also showing a diffuse cytoplasmatic signal (Figure 7C; Supplementary Figure S6B). The number of Ki-67^+^ cells in C1 TDO grown in Matrigel or on 3D-dECMs, was reduced by 95% and 72%, respectively, compared with the untreated control groups (**P < 0.01; Figure 8A). Proliferation was reduced by 80% and 66%, respectively, in C3 organoids grown in Matrigel or on 3D-dECMs, compared to the untreated control groups (**P < 0.01; Figure 8A). Immunofluorescence (IF) staining with cCASPASE3 confirmed that untreated C1 and C3 TDO were alive (Figure 8C, left panel). The number of cCASPASE3^+^ cells in C1 TDO grown in Matrigel or on 3D-dECMs was 80% and 60%, respectively, compared with the untreated control groups (**P < 0.01; Figure 8B), and 80% and 50%, respectively in C3 TDO (**P < 0.01; Figure 8B). MMC treatment had no effect on C2 organoids (Supplementary Figure S6C–E). Regarding OXA treatment, the number of cCASPASE3^+^ cells in TDO grown in Matrigel or on 3D-dECMs was 50% and 70% for C1, 60% and 85% for C2, and 45% and 65% for C3, compared with untreated control groups (**P < 0.01; Supplementary Figure S6F–H). These results demonstrate that HIPEC induces apoptosis in TDO and the presence of 3D-dECMs hinders its efficacy (Figures 7 and 8).

## Discussion

Here, we describe an engineered model that combines key features of TDO within the microenvironment, enabling the recapitulation of the PM niche under physiological conditions. Our findings are in line with previous works showing that cancer-derived ECM sustains the proliferation and invasion of CRC cell lines (D'Angelo et al., 2020).

Scaffolds derived from neoplastic peritoneum showed greater stiffness than those derived from normal peritoneum. Increased stiffness and crosslinking of the perilesional ECM was identified as an environmental change predisposing to CRC invasion and can be attributed in part to the more compact fine structure of the matrix and to the linearization of the fibers in bundles (Nebuloni et al., 2016). The increased amount of GAG observed in the perilesional ECM may also be related to increased stiffness. In fact, some authors have shown that negatively charged GAGs provide a repulsive force that opposes compression and shear in the ECM (Buschmann and Grodzinsky, 1995); in addition, the ability of GAGs to retain high quantities of water and hydrated cations confers resistance to compressive forces (Buschmann and Grodzinsky, 1995). The high amount of GAGs observed in neoplastic tissues could be the result of metastatic transformation. In fact, the sulfated GAG (sGAG) chondroitin-sulfate is the main binding site for the isoform ν of CD44, which is a key player in metastatic spread (Zanconato et al., 2019). Transcriptome analysis also confirmed higher expression of GAGs in the repopulated neoplastic 3D-dECMs.

Our observations support the proposed role of *YAP* as a tumor suppressor in metastatic CRC, where it can inhibit the Wnt pathway by reprogramming *LGR5*^+^ cells and, *in vivo*, by reducing Wnt activity (Cheung et al., 2020). Indeed, Wnt pathway activity was one of the most represented Gene Ontology (GO) categories higher in TDO grown on 3D-dECMs than in Matrigel. Analysis of YAP/TAZ expression in tumor tissue from which TDO were derived corroborates this hypothesis. The presence of YAP/TAZ in TDO grown in Matrigel is likely due to the different stiffness of Matrigel compared with 3D-dECMs and the absence of factors previously released from the tumor cells, which are instead present in the 3D-dECMs and could regulate YAP/TAZ signaling activity (Panciera et al., 2017). This result highlights how commercially available substrates fail to fully reproduce the *in vivo* context. Factors other than YAP/TAZ signaling, present in the stromal compartment, could influence the mechanobiology of the peritoneum. For example, cancer-activated fibroblasts can activate a complex signaling network capable of remodeling and/or removing the ECM, through the activation of the TGFβ pathway (Sanders et al., 2015). The interactions between the ECM and stromal and tumor cells could also regulate the secretion of specific molecules that can induce fibrosis through cytokines and growth factors-mediated pathways (Vasiukov et al., 2020). Indeed, the cytokine signaling-mediated pathway was one of the most represented GO categories observed. These data indicate the development of a complex cell–cell and cell–ECM communication network and suggest a prominent role of the stromal compartment in the mechanical characteristics of the peritoneal ECM. Future experiments will be needed to elucidate how stromal cells can affect mechanobiology of the peritoneal ECM through co-culture of tumor cells, ECM, and stromal cells.

Morphological and topographical experiments showed that the neoplastic scaffold undergoes complex structural modifications that enhance TDO adhesion and proliferation. In support of this concept, both normal and tumor 3D-dECMs showed an upregulation of genes involved in zinc and copper metabolism and homeostasis, which was greater in the neoplastic peritoneum-derived matrix. These metal ions are involved in the regulation and activation of the metalloproteinase enzymes, which are the main modulators of the ECM through a proteolytic activity (Ceelen et al., 2020) and could play a role in the activation of ECM remodeling pathways underlying the metastatic niche.

Transcriptomic analyses showed that repopulated 3D-dECMs presented typical features of the PM disease (Jayne et al., 2002; Siegel et al., 2014) and expressed genes involved in ECM remodeling, such as NABA Matrisome and stem cell-related genes, ECM regulators, and genes involved in the response to cytokine and pro-inflammatory stimuli, integrin interactions and collagen/proteoglycan modifications, epithelial cell differentiation, resistance to compression, and regulation of angiogenesis (Bajou et al., 2008; Schlesinger and Bendas, 2015). These pathways were less represented in normal 3D-dECMs and absent in Matrigel samples, confirming a previous study showing that growth on the ECM of normal colon organoids transfected with mutant *APC* induces features typically associated with CRC progression (Chen et al., 2016). The deregulation of genes belonging to pathways involved in the metastatic process, related to metastatic spread and the development of the metastatic niche (Varghese et al., 2007; Lemoine et al., 2016), is in agreement with the fact that our organoids are derived from metastatic lesions, where the cells activate a series of pathways to better fit to the niche.

HIPEC simulation experiments highlighted the potential role of the neoplastic ECM in the development of drug resistance. Transcriptomic data indicated the activation of mechanisms related to drug resistance along with the modification of the mechanical properties of the ECM. In fact, growth on scaffolds increased the expression of anti-apoptotic and pro-survival genes, as well as genes involved in platin-based drug resistance. High expression of stiffness-related genes was also observed. Integrins, which are involved in ECM remodeling and can function as mechanotransducers, contributing to cancer metastasis, stemness, and drug resistance (Su et al., 2020), were also one of the most representative GO categories, supporting the activation of the drug resistance mechanism when TDO are grown on 3D-dECMs. Dose–response curves to MMC and OXA showed that the TDO had different drug sensitivities and that clinically administered doses of MMC and OXA were insufficient to eliminate all cancer cells. Response to treatment was even less for TDO grown on 3D-dECMs, which showed greater resistance to both drugs. In support of these findings, genes involved in drug resistance, especially to platinum-based compounds, were upregulated in TDO grown on 3D-dECMs. The results observed with the model better reproduce the results in the clinics, as ∼60% of patients treated with HIPEC relapse in one year. All these findings highlight that our proposed engineered model could be a drug screening tool that can more faithfully recapitulate the tumor microenvironment and treatment response for tailored therapies than the classical monoculture two-dimensional (2D) models, or even 3D-cultures, which are still being used (Ghajar, 2015).

However, the model has some limitations, as it does not yet reach a level of resolution that allows it to mimic the PM niche in all its constituents. In fact, other components of the microenvironment play a key role in PM spread (Hussey et al., 2017; Bleijs et al., 2019), such as immune surveillance and the vascular system (Seebauer et al., 2016; Mohrmann et al., 2020). Further optimization of our model will therefore imply the reconstruction of a specialized physiological microenvironment by incorporating vascular networks, the immune system, and organ-specific microbes. In addition, the replacement of patient-derived 3D-dECMs with a synthetic support with the same biochemical and physical characteristics of the components of the decellularized matrix will improve the reproducibility and enable personalized drug screenings on the TDO.

Our model represents a physiological tool that could aid the identification of key players in the metastatic development and may allow the selection, in a biologically relevant context, of new therapeutic strategies. Finally, the approach described here could be used to generate other types of *ex vivo* metastatic niches.

## Materials and methods

### Human tissues

Peritoneal tissue was collected from six patients with peritoneal metastatic colorectal carcinoma, who underwent surgical resection at the Peritoneal Malignancies Unit of our Institution. The patients were staged according to the WHO classification (Ubink et al., 2018). The study was approved by the Institutional Review Board (134/13; 149/19) and was conducted in accordance with the Declaration of Helsinki, 2009. Written informed consent was acquired. Metastatic lesions and apparently normal tissue (>10 cm from the metastatic lesions) were harvested and one part of the metastatic tissue (1 cm in diameter) was placed in ice-cold phosphate-buffered saline (PBS; ThermoFisher Scientific) containing gentamicin (50 ng/ml, ThermoFisher Scientific) and amphotericin B (50 ng/ml, ThermoFisher Scientific) for the generation of PM-derived organoids, while a second specimen was frozen in liquid nitrogen for molecular and histopathological analyses. The remaining tissue was used to develop 3D-decellularized matrices (3D-dECMs).

Normal tissue was partly used to develop dECMs and partly frozen for further studies. FFPE blocks were prepared for IHC analyses of normal and metastatic tissue.

### Development of PM-derived organoids

PM-derived TDO were developed as described in Fujii et al. (2016) and Bozzi et al. (2017). TDO were grown in basal cell culture medium consisting of advanced DMEM-F12 (ThermoFisher Scientific) and supplemented with different growth factors (Supplementary Table S2) to mimic different niche factor conditions, as described in Fujii et al. (2016). Incubation was performed at 20% O_2_ and 5% CO_2_. After expansion, the TDO were cultured in cell culture medium lacking growth factors, which was refreshed every three days. Optimal cell culture medium conditions were determined separately for each organoid culture (Supplementary Table S3).

Organoids were split every 1–2 weeks as follows: they were mechanically removed from the Matrigel by pipetting, incubated in Cell Recovery Solution (Corning) for 1 h at 4°C, washed twice with ice-cold PBS, and seeded as described above.

Aliquots of each organoid culture were frozen or prepared for IHC analyses as follows: samples were fixed in 10% formalin at room temperature for 10 min and embedded into 200 µl Bio-Agar (Bio-Optica). The samples were then cooled at –20°C until solidification. For each sample, sections of 3 µm thickness were obtained.

### Preparation of 3D-dECMs

3D-dECMs were derived from both PM and the corresponding normal peritoneum. Each experiment was conducted using 3–10 different surgical specimens derived from different patients.

The decellularization was performed as described in Genovese et al. (2014). Briefly, both PM and normal peritoneum samples (60–100 mg wet weight) were washed with ice-cold PBS supplemented with 50 ng/ml gentamicin and 50 ng/ml amphotericin B, followed by treatment with solutions containing detergents and enzymatic agents.

The success of the decellularization procedure was evaluated by analyzing the DNA content of the 3D-dECMs. The 3D-dECMs were then washed with ice-cold PBS and either transferred into chilled freezing solution (90% DMEM-F12 and 10% dimethyl sulfoxide) and frozen for storage or fixed for IHC and IF analyses. All the decellularization experiments were performed in triplicate, using at least three different samples, each derived from a different donor.

### Ex vivo engineered PM lesion

TDO were removed from the Matrigel as described above and dissociated into single-cell suspensions with trypsin–EDTA by vigorous pipetting for 10 min. 3D-dECMs derived from normal peritoneal tissue and PM were incubated overnight at 37°C in DMEM-F12 supplemented with 10% fetal bovine serum (FBS; Euroclone) and 50 ng/ml gentamicin and amphotericin B. Then, 1 × 10^6^ cells were resuspended in 1 ml cell culture medium (Supplementary Table S2) and seeded on the top of 50 mg of 3D-dECMs. Repopulated matrices were placed in a 24-well cell culture plate (Corning) containing DMEM-F12 supplemented with 10% FBS and 50 ng/ml gentamicin and amphotericin B, followed by incubation for 2 h at 37°C. Each well was filled with 2 ml cell culture medium, which was changed every two days. Repopulated matrices were either frozen for RNA extraction or fixed for IHC and IF analyses. Representative 3-µm FFPE sections were cut at different depths to verify the presence of TDO in the inner part of the 3D-dECM scaffold. The repopulation experiments were performed in triplicate, using different neoplastic and normal peritoneum-derived matrices obtained from three different donors.

### Treatment with cytotoxic drugs

MMC (Kyowa Kirin Co., Ltd) and OXA (Fresenius Kabi) were used for the *in vitro* simulation of HIPEC treatment. MMC was dissolved in dimethyl sulfoxide to obtain a 60 mM stock solution. OXA was diluted in physiological solution (0.45% sodium chloride and 2.5% glucose) to obtain a 15 mM stock solution. Both drugs were diluted to the working concentration in the cell culture medium, where the final solvent concentration was <0.1% for all samples, including controls. The experiments were performed in triplicate, using different neoplastic and normal peritoneum-derived matrices obtained from three different donors.

### Ex vivo PM lesion to test HIPEC efficacy in vitro

PM-derived organoids were grown on the top of neoplastic 3D-dECMs in a 24-well cell culture plate for 12 days in order to allow a complete colonization of the matrix (Genovese et al., 2014; Chen et al., 2016; Weijing et al., 2021). TDO grown in Matrigel were used as control to evaluate the impact of native 3D-dECMs on the HIPEC treatment. The engineered PM lesions were treated with preheated MMC and OXA at a concentration of 41.9 µM for 60 min at 42.5°C and 252 µM for 90 min at 42.5°C, respectively, corresponding to the calculated clinical concentrations, which is in line with the current standard protocols used for HIPEC at Fondazione IRCCS Istituto Nazionale dei Tumori, Milan, Italy. After the treatments, the PM models were subjected to three washes with 1× PBS and incubated for 48 h with appropriate cell growth medium.

Samples were fixed in formalin and FFPE sections were obtained as described above. The impacts of HIPEC treatment on TDO proliferation and the activation of an apoptotic program were determined by Ki-67 and cCASPASE3 immunostaining, respectively. All the experiments were performed in triplicate, using different neoplastic and normal peritoneum-derived matrices obtained from three different donors.

## Supplementary Material

mjac064_Supplemental_File
