# Supplementary material for: Decellularized extracellular matrix as scaffold for cancer organoid cultures of colorectal peritoneal metastases
Source: J Mol Cell Biol. 2022 Dec 2;14(11):mjac064. doi: 10.1093/jmcb/mjac064 (PMC10170410; doi:10.1093/jmcb/mjac064)
Supplement: mjac064_Supplemental_File [file mjac064_supplemental_file.pdf]

Supplementary Matherial and Methods

Nucleic acids extraction

DNA from FFPE sections of the PM-derived organoids and their tissue of origin was used for mutational analysis. DNA was extracted using the Masterpure Complete DNA Purification Kit (Lucigen-Biosearch Technologies, Middleton, WI, USA) and quantified on the QIAxpert® spectrophotometer (QIAGEN, Hilden, Germany).

DNA from 20 mg of normal peritoneum and PMs, both decellularized and untreated, was used to evaluate the success of the decellularization procedure. DNA was extracted using the DNeasy Blood&Tissue kit (QIAGEN) according to the manufacturer's instructions, and quantified using Nanodrop 1000 (ThermoFisher Scientific) at 260/280 nm ratio. DNA from decellularized ECM, normal peritoneum, PM, and their corresponding non-decellularized samples was loaded onto a 1% agarose gel. The separated bands were visualized by exposing the gel to UV light and images were acquired using Gel Doc (Bio-Rad, Hercules, CA, USA). All of the experiments were performed in triplicate.

RNA from the three organoid cultures (C1, C2 and C3) grown both in Matrigel and on normal or neoplastic peritoneal 3D-dECMs was used for RNA-sequencing (RNA-seq) analyses. For the PM organoids, the Matrigel was digested with Cell Recovery Solution (Corning) as described above. The pellet was washed three times with ice-cold PBS and suspended in 1 ml TRIzol™ reagent (QIAGEN). Instead, for the repopulated 3D-dECMs, the matrices were washed three times with ice-cold PBS and homogenized using the TISSUE Tearor Homogenizer (QIAGEN) in 500 µl TRIzol™ reagent (QIAGEN). Then, RNA was extracted following the manufacturer's instructions, quantified on a ND-1000 spectrophotometer (ThermoFisher Scientific) and stored at -80 °C.

RNA from FFPE sections (10 µm) of the PMs from which the six TDO were derived was used to validate the results from the RNA-seq analysis. RNA was extracted using the miRNeasy FFPE kit (QIAGEN) and quantified on a NanoDrop™ 1000 (ThermoFisher Scientific, Waltham, MA).

Histochemistry (HC), IHC and IF

Before HC and IHC staining, FFPE sections were cut into slices and dewaxed in xylene, rehydrated through decreasing concentrations of ethanol and washed with water. Slices were stained with H&E for quality control. For HC analysis, sections were stained with Masson's trichrome (Aniline blue kit; Bio-Optica), Alcian blue stain (pH 2.5 kit, Bio-Optica), van Gieson trichrome (Bio-Optica), and Periodic Acid Schiff (PAS, Bio-Optica) following the manufacturers' instructions. IHC was performed using the following mouse anti-human antibodies: Ki-67, CK19, CK20, CK AE1/AE3, CDX2, LGR5, vimentin, YAP and TAZ. Images were acquired with a DM6000B microscope (Leica). Staining for Ki-67, CK19, CK20, CK AE1/AE3, CDX2, LGR5 and vimentin antibodies was performed automatically using theAutostainer Link 48 (Dako, Agilent, Santa Clara, CA. US). Antigen retrieval for YAP and TAZ antibodies was carried out using preheated target retrieval solution (pH 6.0) for 30 minutes. Tissue sections were blocked with FBS serum in PBS for 60 min and incubated overnight with primary antibodies. The antibody binding was detected using a polymer detection kit (GAM/GAR-HRP, Microtech) followed by a diaminobenzidine chromogen reaction (Peroxidase substrate kit, DAB, SK-4100; Vector Lab). All sections were counterstained with Mayer's hematoxylin. Dilutions and experimental conditions are listed in Supplementary Table S3. For IF analyses, FFPE sections were stained with Alexa680-conjugated Wheat Germ Agglutinin (WGA) marker (ThermoFisher Scientific) and DAPI (VECTASHIELD Mounting Medium with DAPI**,** Maravai LifeScencies, San Diego, CA, USA), with anti-human Ki-67 and LGR5 monoclonal antibodies, with anti-human Collagen-IV and anti-human monoclonal cCASPASE3 antibodies and DAPI, followed by Alexa488-conjugate goat anti-mouse or Alexa546-conjugated

goat anti-rabbit IgG polyclonal secondary antibodies for 1 hour at RT in dark (ThermoFisher Scientific). Images were acquired with a DM6000B microscope (Wetzlar, Germany Leica,) equipped with a 100 W mercury lamp, and analyzed using Cytovision software (Leica). Dilutions and experimental conditions are listed in Supplementary Table S4.

DNA sequencing

About 150–200 ng genomic DNA (measured with Qubit dsDNA HS assay kit, ThermoFisher Scientific), were sheared by the Sure Select Enzymatic Fragmentation kit (Agilent Technologies Inc., Santa Clara, CA, USA). NGS libraries were created using Sure Select XT2 Low input Custom library probes (Agilent Technologies Inc.). The probe set was custom designed by Cogentech (OncoPan panel) and includes the exonic regions of the following genes: APC, ATM, BARD1, BMPR1A, BRCA1, BRCA2, BRIP1, CDH1, CDKN2A (α and β isoform), CDK4 (exon 2), CHEK2 , CTNNA1, EPCAM , FANCM, MLH1, MSH2, MSH3, MSH6, MUTYH, NBN, NHTL1, PALB2, PMS2, POLD1, POLE, PTEN, RAD51C, RAD51D, SMAD4, STK11, TP53, KRAS, NRAS, BRAF, EGFR, HER2 (ERBB2), and PIK3CA. Sequencing was performed on Illumina MiSeq platform, in PE mode (2 x 150 bp). Raw reads were demultiplexed and aligned to a reference genome (Human GRCh37) using a pipeline developed in-house in collaboration with enGenome Software Company and annotated with the eVai tool. Results were compared to find the percentage of common SNVs (Single Nucletode Variants). Five PM-derived organoids (C1, C2, C3, C4 and C6) and their corresponding surgical samples were analyzed. FFPE tissue for C5, unfortunately,was not available.

Morphological evaluation of the decellularized matrices

3D-dECMs from normal peritoneum and PM lesions were washed twice with 1X PBS and placed in a 60 mm petri dish. Samples were illuminated with a widefield lamp laser to visualize the architecture of the collagen fibers. An image format of 1024x1024 pixels was used and all images were acquired with Leica Application Suite X, ver. 3 software. 3D-dECMs FFPE sections deriving from normal and PM peritoneum were used to perform polarized light microscopy (PLM). FFPEs were analyzed with an Olympus BX63 upright widefield microscope equipped with a motorized stage and a Hamamatsu OrcaAG camera, using Metamorph software. UplanSApo 4X/0.16 N.A objective was used to acquire the mosaics of the sections. Insets were acquired with UplanSApo 10X/0.4 N.A. and UplanSApo 20X/0.75 N.A. objectives. All experiments were perfomed at least in duplicate. Confocal reflection microscopy images were acquired with a Leica TCS SP8 laser confocal scanner mounted on a Leica DMi8 microscope through a HC PL FLUOTAR 20×/0.5 NA.

Nanoscale topographical analysis of 3D-dECMs

The topographical evaluation of the 3D-dECMs was performed by atomic force microscopy (AFM) analysis on samples deriving from normal peritoneum and PM of three different patients. Before the AFM analysis, the 3D-dECM slides were left for 30 minutes at RT to dissolve the optimal cutting temperature (OCT) compound. Then, the samples were carefully washed with ultrapure water and covered with 1X PBS buffer. AFM topographic measurements were carried out at RT using a NanoWizard3 AFM (JPK, Germany) coupled to an Olympus BX61 inverted microscope and equipped with tapping mode silicon ACTG AFM probes (APPNANO). The 50 µm thick tissue slices, instead, were mounted on polarized glass slides (ThermoFisher Scientific), left for 30 minutes at RT and carefully washed with ultrapure water. The topography of each tissue was

characterized by collecting at least 10 areas (5x5 μm^2) of the sample surface with 512×512 points (scan speed 3,5 $\mu m\ s^{-1}$).

ECM component quantification

Total collagen and sulphated glycosaminoglycan (sGAG) content in fresh and decellularized normal and PM peritoneum were quantified using the SIRCOL collagen assay (Biocolor, Carrickfergus, UK) and the Blyscan GAG assay kit (Biocolor), respectively. The experiments were performed in triplicate following the manufacturer's instruction. Data are the mean of three different neoplastic and normal-derived samples obtained from three different donors.

Nanoindentation measurements by AFM

AFM mechanical analysis was carried out on 3D-dECMs deriving from normal peritoneum and PM of five patients. 3D-dECMs were embedded in OCT and frozen with nitrogen-cooled 2-propanol for 10 seconds. Slices of 100 µm thickness were cut with a microtome (Leica) and attached to positively charged poly-lysine coated glass coverslips (ThermoFisher Scientific), exploiting the electrostatic interaction. Nanomechanical tests were performed in liquid on samples covered by a PBS droplet confined by a circular ridge of hydrophobic two-component silicone paste (Leica). A Bioscope Catalyst AFM (Bruker) was used, which was resting on an active anti-vibration base (DVIA-T45, Daeil Systems) and put into an acoustic enclosure (Schaefer). The measurements were performed at RT. Custom monolithic borosilicate glass probes consisting of spherical glass beads (SPI Supplies), with radii R in the range of 7.5–12.5 µm, were attached to tipless cantilevers (Nanosensor, TL-FM) with nominal spring constant k = 3-6 N/m. Probes were fabricated and calibrated, in terms of tip radius, according to an established custom protocol (Indieri et al., 2011). The spring constant was measured using the thermal noise calibration (Hutter and Bechoefer, 1993)

and corrected for the contribution of the added mass of the sphere (Chighizola et al., 2021; Laurent et al., 2013;). The deflection sensitivity was calibrated *in situ* and non-invasively before every experiment by using the previously characterized spring constant as a reference, according to the SNAP procedure described in (Schillers et al., 2017).

The mechanical properties of the 3D-dECMs were obtained by fitting the Hertz model to sets of force versus indentation curves (simply force curves, FCs), as described elsewhere (Schillers et al., 2017; Nebuloni et al., 2016; Puricelli et al., 2015; Shimshoni et al., 2020), to extract the value of the YM of elasticity, which measures ECM rigidity. FCs were collected in Point and Shoot (P&S) mode, selecting the regions of interest from optical images, exploiting the accurate alignment of the optical and AFM images obtained using the Miro software module integrated in the AFM software. Each set of FCs consisted of an array of typically 15x15=225 FCs spatially separated by 5-10 µm, each FC containing 8192 points, with ramp length L = 8-15 µm, maximum load Fmax = 150-1500 nN, and ramp frequency f = 1 Hz. The maximum load was chosen in order to achieve a maximum indentation in the range of 4-9 µm. Typical approaching speed of the probe during indentation was 16-30 µm/s. Five samples were characterized for each condition. In each sample, 3-10 P&S were acquired in macroscopically separated locations, for a total of 10-25 independent P&S per patient and condition (up to 2250-5500 FCs per patient and condition).

Stem cell maintenance, proliferation and apoptosis assays

Growing cells, stem cells and apoptotic cells were detected on FFPE sections. Growing cells, deriving from disaggregated TDO, were stained with anti-human Ki-67 monoclonal antibody (clone MIB-1) and DAPI, and their growth rate was expressed as the percentage of Ki-67-positive cells present in fields devoid of dead cells. Stem cells were stained with anti-human LGR5 monoclonal antibody (clone OTI2A2) and DAPI, and their density was expressed as the percentage of LGR5-

positive cells present in fields devoid of dead cells. Apoptotic cells were stained with anti-human cCASPASE3 monoclonal antibody (clone 9661) and DAPI, and the apoptotic rate was calculated as the percentage of cCASPASE3-positive cells present in the field. The percentage of Ki-67-positive, LGR5-positive and cCASPASE3-positive cells was obtained by dividing the number of positive cell present in one field by the total number of cells in one field, multiplied by 100. Cells in three independent fields (40X magnification) were counted using ImageJ software. The experiments were performed in triplicate using three different neoplastic and normal-derived matrices obtained from three different donors.

Qpath analyses

Percentage estimation and cell counting were performed using Qupath software (https://qupath.github.io, version 0.2.3). The images used for Qpath analyses were acquired using Aperio Leica ScanScope XT (Leica Biosystems, Wetzlar, Germany). The slides were evaluated by an expert pathologist. The percentage of CK AE1/AE3, CK20, CK19, CDX2, Ki-67, and LGR5 positive cells was calculated by dividing the number of positive cells present in each field by the total number of cells in the same field. TDO-derived infiltrating cells were evaluated by calculating the total number of H&E stained cells. Three fields were counted per experiments.

RNA-seq analysis

Gene expression profiles were conducted on C1, C2 and C3 organoid cultures grown in Matrigel and on 3D-dECMs. Total RNA was extracted using TRIzol™ reagent (QIAGEN). Qubit fluorimeter (ThermoFisher Scientific) and Agilent Bioanalyzer 2100 (RIN > 8) were used to measure and assess RNA abundance and integrity, respectively. Indexed library preparation was performed starting with 500 ng total RNA with the TruSeq stranded mRNA (Illumina) according to

the manufacturer’s instructions. RNA-seq was performed in PE mode (2x75nt) on an Illumina NextSeq550 platform, generating an average of 55 million PE reads per sample. For every condition (Matrigel, normal 3D-dCM and neoplastic 3D-dECM), two replicates per organoid were sequenced, for a total of 18 data points. Raw reads were aligned to the human transcriptome (hg38) with STAR (Dobin et al., 2013) using the quantMode option to generate transcripts counts. Differentially expressed genes in the three growth conditions were identified with DESeq2 (Alshehri, 2018). All *p*-values were adjusted for false discovery rate with the Benjamini-Hochberg method.

Gene Set Enrichment Analysis

Gene Set Enrichment Analysis was performed with the enrichR R package (Chen et al., 2013) on deregulated genes (absolute fold change > 2 and adjusted *p*-value <0.05). In particular, the enrichment for the Matrisome database was assessed. This database provides live cross-referencing to gene and protein databases for every ECM and ECM-associated gene, also integrating experimental proteomic data on ECM and ECM-associated proteins and genes from the ECM Atlas (Naba et al., 2016). Gene sets with adjusted *p*-value <0.05 were considered significantly enriched.

Quantitative real-time polymerase chain reaction (qRT-PCR)

For gene expression analysis, cDNA was synthesized from 100 ng of total RNA using a High-Capacity cDNA Reverse Transcription Kit (ThermoFisher Scientific, Waltham, MA) and qPCR was carried out with gene-specific assays for MT1A (Hs00831826_s1), LOX (Hs00942480_m1), THY1 (Hs00174816_m1), FZD9 (Hs00268954_s1), SPP1 (Hs00959010_m1), and performed using the TaqMan FAST Universal PCR Master Mix, no AmpErase® UNG in a PRISM 7900HT Real-Time PCR system (Thermo Fisher Scientific). The expression values of the genes were normalized to GAPDH (Hs99999905_m1).

Dose-response curves for HIPEC treatment

To determine the IC_{50} value of MMC and OXA, $5x10^3$ C1, C2 and C3 TDO were suspended in 100 µl of culture medium and seeded on 96-well plates (Costar 3904; Corning, New York, USA) coated with 40 µl of Matrigel. TDO were dispensed on the top of the matrigel. After two days, TDO were incubated with 100 µl preheated drug at concentrations ranging from 2.5 to 200 µM for MMC and between 10 and 700 µM for OXA, for 60 min (MMC) or 90 min (OXA) at 42.5 °C. The values were chosen by scaling up and down the concentrations used for patients (35 mg/m^2 for MMC and 200 mg/m^2 for OXA, which correspond to 41.9µM for MMC and 252 µM for OXA for *in vitro* treatments (Ubink et al., 2019). TDO viability, was assessed using a CellTiterGlo® 2.0 kit (Promega, Fitchburg, Wisconsin, USA) on a TECAN spark microplate reader (Tecan Trading AG, Switzerland). Viability was normalized to the mean of three control samples/plate (TDO treated with 0.5 % DMSO in MMC and physiological solution in OXA experiments). All the experiments were performed in triplicate.

Immunoblotting

After HIPEC simulation using drugs at a concentration corresponding to the IC_{50} value of each TDO, C1, C2, and C3 TDO were lysed in Ripa buffer (50 mM Tris, pH 8.0, 50 mM NaCl, 0.5% Triton X-100, 0.1% sodium deoxycholate, 0.25% sodium dodecyl sulphate [SDS]) supplemented with protease inhibitors (Merck Millipore, Billerica, MA, USA) and a phosphatase inhibitor cocktail (Sigma-Aldrich, St. Louis, MO, USA), for 3 h at 4 °C on a rotation wheel. Samples were then sonicated and their protein content was quantified using the Bradford protein assay (Bio-Rad, Hercules, CA, USA). For each sample, 40 µg of protein extract were separated on 4–12% polyacrylamide gels, transferred onto nitrocellulose membranes (Sigma-Aldrich, St. Louis, MO, USA) and incubated with primary antibodies (Supplementary Table S4). The signals were detected

using enhanced chemiluminescence, and protein levels were quantified using Imagelab software (Bio-Rad, Hercules, CA, USA). Each experiment was repeated at least three times.

Statistical analyses

Statistical analyses were performed using GraphPad Prism software (version 8.4.1 (676), GraphPad Software, San Diego, USA). Data are expressed as mean and SEM. A two-tailed Student's t test was used to compare paired groups. Differences among groups were evaluated using two-way ANOVA. In the case of AFM mechanical experiments, for each patient and each condition tested, the median values of the YM were extracted from each measured location (P&S) using the procedure described in Cramer et al (Shimshoni et al., 2020; Cramer, 1999). The distributions of the measured YM values were obtained by grouping all P&S measured in all locations, for each patient and each condition tested. The mean and median values and the corresponding standard deviations of the mean (as SEM) were calculated by averaging between P&S. The statistical significance of differences between normal and neoplastic conditions was estimated by applying the two-tailed *t* test. A *p*-value <0.05 was considered statistically significant.

References

Alshehri H.A.N. (2018). Compare and Contrast of Differential Gene Expression Software Packages of RNA-Seq.*Computational Science and Computational Intelligence (CSCI)*. Las Vegas, NV, USA: IEEE.

Butt H.J. , Jaschke M. (1995). Calculation of thermal noise in atomic force microscopy. *Nanotechnology* 6, 1.

Chen E.Y. , Tan C.M. , Kou Y. , *et al.* (2013). Enrichr: interactive and collaborative HTML5 gene list enrichment analysis tool. *BMC Bioinformatics* 14, 128.

Chighizola M. , Puricelli L. , Bellon L. , Podestà A. (2021). Large colloidal probes for atomic force microscopy: Fabrication and calibration issues. *J. Mol. Recognit.* 34, e2879.

Cramer H. (1999). Mathematical methods of statistics. *Princeton University Press* 1999.

Dobin A. , Davis C.A. , Schlesinger F. , *et al.* (2013). STAR: ultrafast universal RNA-seq aligner *Bioinformatics*. 29, 15-21.

Hutter J.L. , Bechhoefer J. (1993). Calibration of atomic-force microscope tips. *Rev. of Sci. Intrum.* 64, 1-5.

Indrieri M. , Podestà A. , Bongiorno G. , *et al.* (2011). Adhesive-free colloidal probes for nanoscale force measurements: production and Characterization. *Rev. Sci Instrum*. 82, 50-62.

Laurent J. , Steinberger A. , Bellon L. (2013). Functionalized AFM probes for force spectroscopy: eigenmode shapes and stiffness calibration through thermal noise measurements. *Nanotechnology* 7, 225504.

Naba A. , Clauser K.R. , Ding H. , *et al.* (2016). The extracellular matrix: Tools and insights for the "omics" era. *Matrix Biol.* 49, 10-24.

Nebuloni M. , Albarello L. , Andolfo A. , *et al.* (2016). Insight On Colorectal Carcinoma Infiltration by Studying Perilesional Extracellular Matrix. *Sci. Rep.* 4, 22522.

Puricelli L. , Galluzzi M. , Schulte C. , *et al.* (2015). Nanomechanical and topographical imaging of living cells by atomic force microscopy with colloidal probes. *Rev. Sci. Instrum.* 86, 033705.

Schillers H. , Rianna C. , Schäpe J. , *et al.* (2017). Standardized Nanomechanical Atomic Force Microscopy Procedure (SNAP) for Measuring Soft and Biological Samples. *Sci. Rep.* 11, 5117.

Shimshoni E. , Adir I. , Afik R. , *et al.* (2020). Distinct extracellular-matrix remodeling events precede symptoms of inflammation. *Matrix Biol.* s0945-053X, 30103-30107.

Ubink I. , Bolhaqueiro A.C.F. , Elias S.G. , *et al.* (2019). Organoids from colorectal peritoneal metastases as a platform for improving hyperthermic intraperitoneal chemotherapy. *Br. J. Surg.* 106, 1404-1414.

Supplementary Table S1

ID	Gender	Diagnosis	Grade	Mutations	Microsatellite	Chemotherapy	PM Organoid line
S07-7576	F	Moderately differentiated infiltrating adenocarcinoma	pT3G2	G12S KRAS;TP53	MSS	None	C1
S11-2361	M	Poorly differentiated mucinous multiple intestinal adenocarcinoma	pT4G3	V600E BRAF; TP53	MSS	None	C2
S16-8598	F	Intestinal mucinous adenocarcinoma	pT4G3N2	G12S KRAS; TP53; FGFR1 amplification	MSS	Yes	C3
S17-3963	F	Moderately differentiated adenocarcinoma	T4aG3N2aM1b	G12S KRAS	MSS	None	C4
S17-3610	M	Intestinal adenocarcinoma with mucinous component	pT4aG2N0Mx	G12S KRAS	MSS	None	C5
S18-8607	M	Colloidal / gelatinous mucinous adenocarcinoma associated with hairy adenocarcinoma of high and low grade	T4aN2bG3Mx	G12S KRAS; TP53	MSS	Yes	C6

Supplementary Table S1: The main pathological characteristics of the patients from whom the tumor specimens were obtained.

Supplementary Table S2

Factor	Description	Vendor	Working Concentration
Gentamicin	Antibiotic	ThermoFisher Scientific	50 ng/ml
HEPES	Buffer	ThermoFisher Scientific	10 mM
L-Glutamine (GlutaMAX)	Cell culture supplement	ThermoFisher Scientific	2 mM
B27	Cell culture supplement	ThermoFisher Scientific	1:50
Gastrin-1, recombinant human	Recombinant protein	Sigma Aldrich	10 nM
N-acetylcysteine	Colonic niche factor	Wako	1 mM
EGF, recombinant human	Recombinant protein	ThermoFisher Scientific	50 ng/ml
Noggin, recombinant human	Recombinant protein	Preprotech	100 ng/ml
R-spondin-1, recombinant human	Recombinant protein	Preprotech	100 ng/ml
Wnt3A, recombinant human	Recombinant protein	Preprotech	50 ng/ml
Prostaglandin E2	Colonic niche factor	Tocris	100 nM
A83-01	p38 inhibitor	Tocris	500 nM
SB202190	ROCK inhibitor	Sigma Aldrich	10 µM

Supplementary Table S2: The complete list of growth factors, media supplements and concentrations used for PM-derived organoid cultures.

Supplementary Table S3

Organoid culture	Medium composition
**C1**	DMEM-F12; B27; Glutamax; N-acetylcysteine; prostaglandin-E2; gastrin-I
**C2**	DMEM-F12; B27; Glutamax
**C3**	DMEM-F12; B27; Glutamax; N-acetylcysteine; prostaglandin-E2; gastrin-I; A83-01
**C4**	DMEM-F12; B27; Glutamax; N-acetylcysteine; prostaglandin-E2; gastrin-I; EGF; A83-01; Noggin
**C5**	DMEM-F12; B27; Glutamax; N-acetylcysteine; prostaglandin-E2; gastrin-I; A83-01; SB202190; Noggin
**C6**	DMEM-F12; B27; Glutamax; N-acetylcysteine; prostaglandin-E2; gastrin-I; A83-01; Noggin

Supplementary Table S3: The specific media composition for each PM-derived organoid culture.

Supplementary Table S4

Antigen (human)	Host	Clone	Vendor	Dilution	Antigen retrieval solution
**Ki-67**	Mouse	MIB-1	Dako	1:400	5 mM EDTA (pH 8), 10 min, 96 °C
**CK19**	Mouse	A53-B/A2	Dako	1:1000	5 mM EDTA (pH 8), 15 min, 96°C
**CK20**	Mouse	Ks20.8	Dako	1:500	5 mM EDTA (pH 8), 30 min, 96°C
**CK AE1/AE3**	Mouse	AE1+AE3	Dako	1:100	10 mM Citrate (pH 6), 15 min, 96°C
**CDX2**	Mouse	CDX2_88	Dako	1:50	5 mM EDTA (pH 8), 30 min, 96°C
**LGR5**	Mouse	OTI2A2	Origene	1:250	10 mM Citrate (pH 6), 15 min, 96 °C
**Vimentin**	Mouse	V9	Dako	1:400	10 mM Citrate (pH 6), 15 min, 96°C
**Collagen-IV**	Rabbit	CIV-22	Abcam	1:200	5 mM EDTA (pH 8), 10 min, 96 °C
**cCASPASE3**	Rabbit	#9661	Cell Signaling	1:250	10 mM Citrate (pH 6), 15 min, 96 °C
**TAZ**	Rabbit	WWTR1	Sigma	1:100	10 mM Citrate (pH 6), 15 min, 96 °C
**YAP**	Mouse	63.7	Santa Cruz	1:100	10 mM Citrate (pH 6), 15 min, 96 °C

Supplementary Table S4: The primary antibodies and the experimental conditions used for IHC and IF analyses.

Supplementary Table S5

Antigen (human)	Vendor	Host	Code	MW	Dilution
**Vinculin**	Sigma	Mouse	V9131	116 kDa	1:10000 in 5% MILK
**Cleaved PARP (Asp214)**	Cell Signaling	Rabbit	9541S	89 kDa	1:1000 in 5% MILK
**p-p53 (Ser15)**	Cell Signaling	Rabbit	9284S	53 kDa	1:1000 in 5% MILK
**p53**	Santa Cruz	Mouse	sc-126	53 kDa	1:1000 in 5% MILK
**Caspase-3**	Cell Signaling	Rabbit	9662	35, 19, 17 kDa	1:1000 in 5% BSA
**Cleaved Caspase-3 (Asp175)**	Cell Sigaling	Rabbit	9661S/L	17, 19 kDa	1:1000 in 5% MILK
**p-Histone H3(Ser10 Thr11)**	Abcam	Rabbit	Ab 32107	17 kDa	1:2500 in 5% BSA
**p-H2AX (Ser139)**	Upstate, Millipore	Mouse	05-636	17 kDa	1:1000 in 5% MILK
**H2AX**	Abcam	Rabbit	Ab11175	15 kDa	1:5000 in 5% MILK

Supplementary Table S5: The primary antibodies and the experimental conditions used for WB analyses.

Supplementary Figure S1

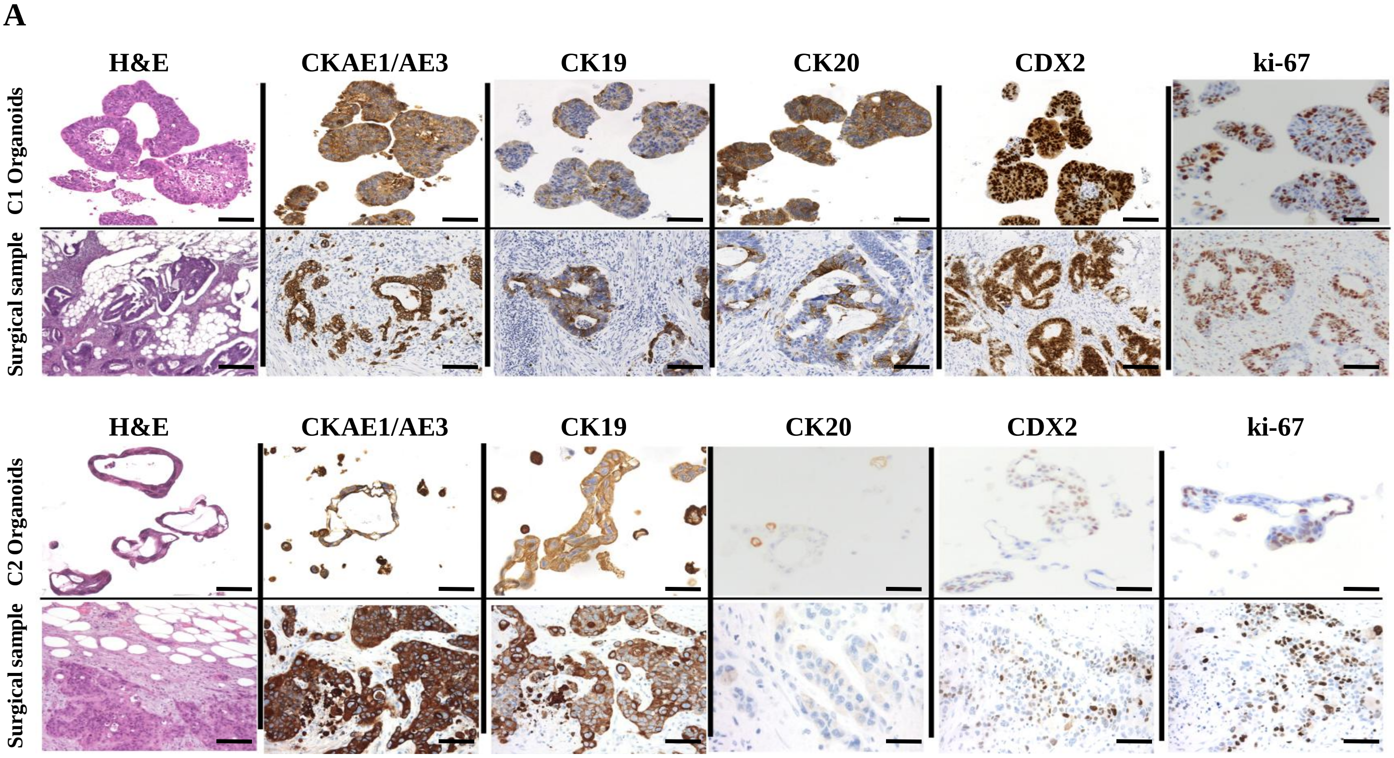

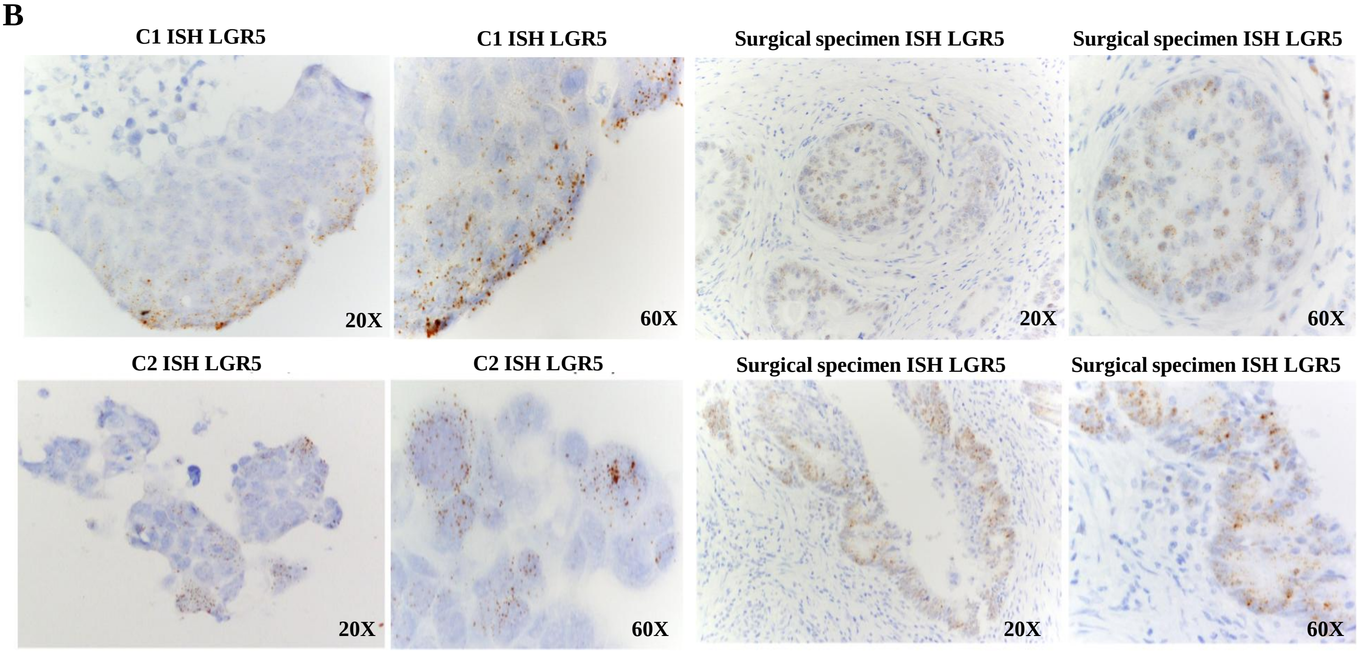

Supplementary Fig. S1. (A) IHC analysis of C1 and C2 organoids and their corresponding surgical samples, using H&E staining and CK AE1/AE3, CK19, CK20, CDX2 and Ki-67 immunostaining (20X magnification). The images were previously published by Bozzi et al. [11]. **(B)** In situ hybridization (ISH) of C1 and C2 organoids and corresponding surgical samples, using LGR5 immunostaining (20X magnification). The images were previously published [11].

C

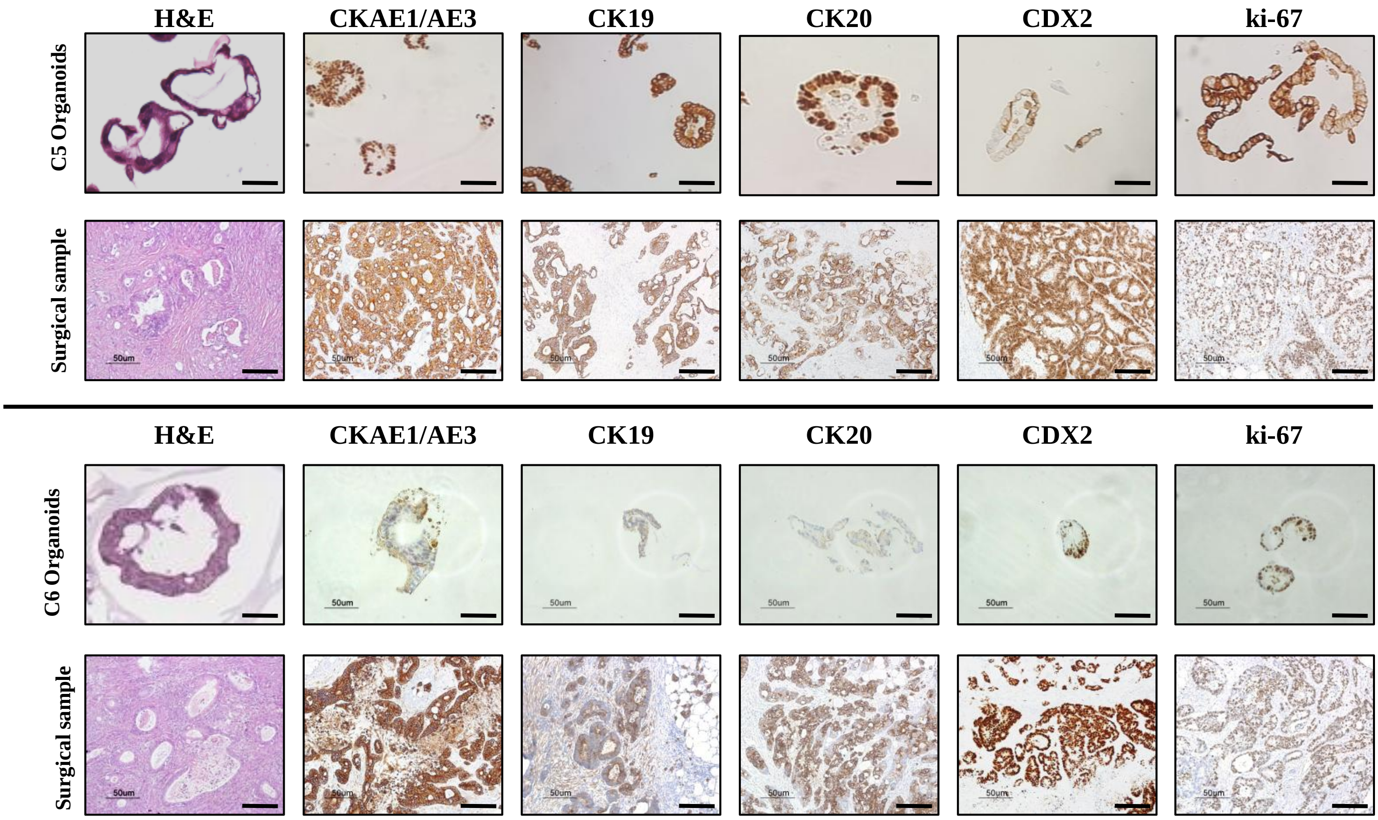

D

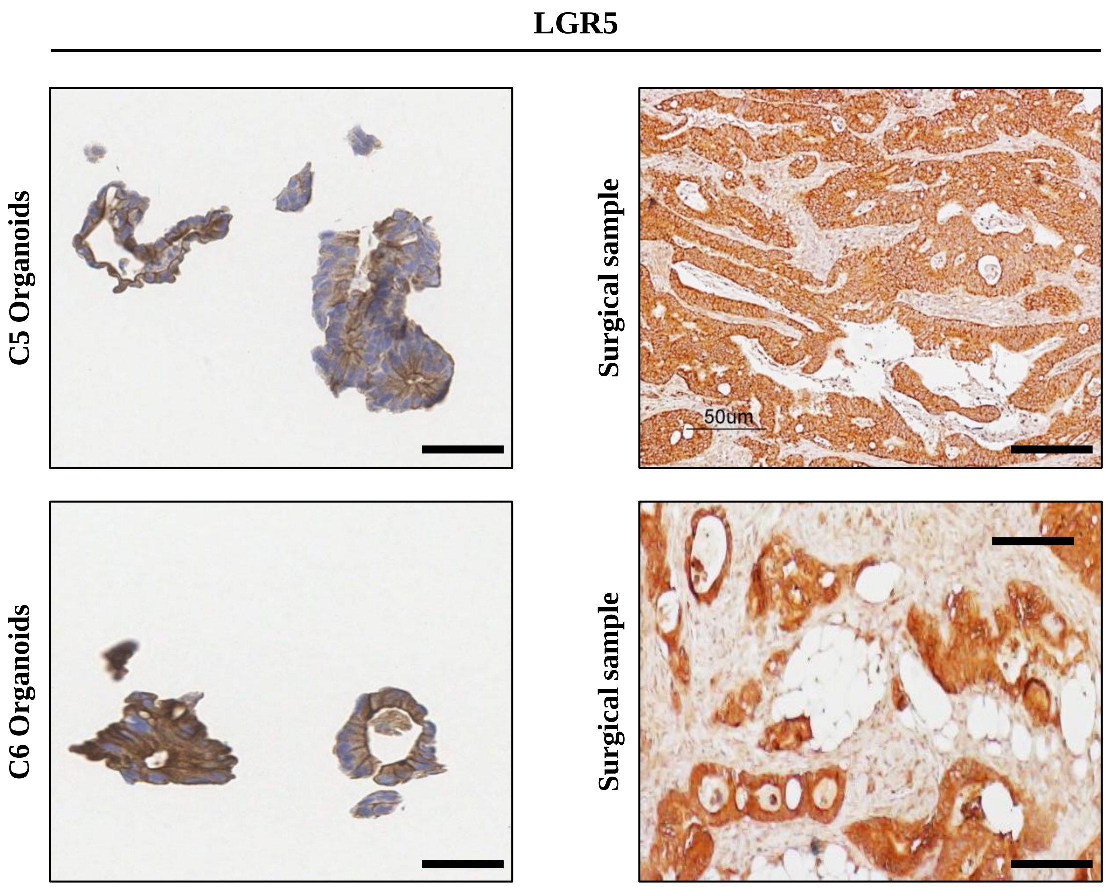

Supplementary Fig. S1. (C) H&E staining and CK AE1/AE3, CK19, CK20, CDX2, and Ki-67 immunostaining of C5 and C6 organoid cultures and their corresponding surgical samples. Scale bar: 50 µm. **(D)** IHC analysis of C5 and C6 organoid cultures and their tumor of origin, using LGR5 immunostaining. Scale bar: 50 µm.

E

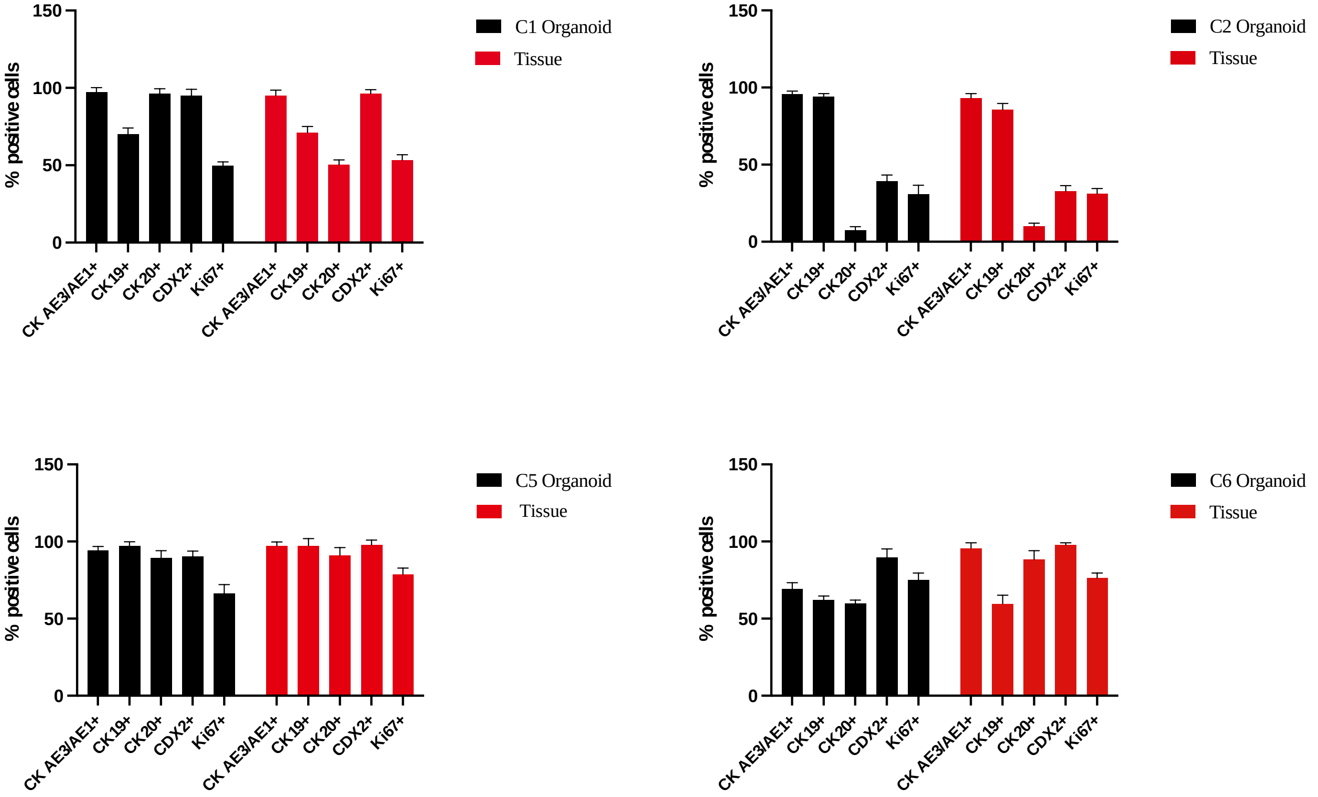

F

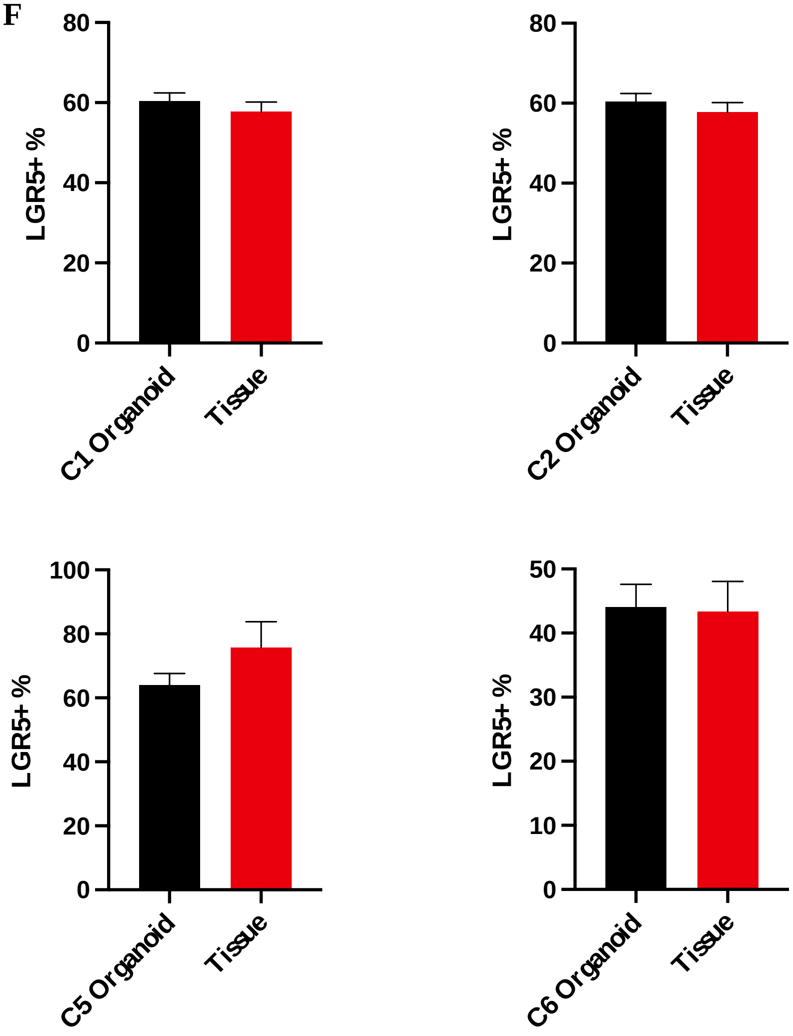

Supplementary Fig. S1. (E) Quantitative counts of the percentage of CK AE/AE3, CK19, CK20, CDX2 and Ki-67 positive cells in C1, C2, C5 and C6 PM-derived organoids Vs their corresponding tumor of origin. Three fields per experiments were counted using Qpath software. Data are presented as median and SD. One-way ANOVA did not show differences between the two groups. **(F)** Quantitative counts of the percentage of LGR5 positive cells in C1, C2, C5 and C6 PM-derived organoids Vs their corresponding tumor of origin. Three fields per experiments were counted using Qpath software. Data are presented as median and SD. One-way ANOVA did not show differences between the two groups.

G

Genes	C1 Organoid	Tissue of origin
MSH3 (A62P)		
BRCA2 (G267Q)		
BRCA2 (S2414S)		
POLE (L1903L)		
PMS2 (S260S)		
BRCA2 (K1132K)		
RAD51C (D253H)		
POLE (A252V)		
BRCA2 (V2466A)		
ATM (D1853N)		
BRCA1 (E1038G)		
BRCA2 (V2171V)		
EGFR (N158N)		
ERBB2 (A356D)		
BRCA1 (S694S)		
BRCA1 (K1183R)		
BRCA1 (L771L)		
BRCA1 (D693N)		
BRCA1 (P871L)		
BRCA1 (S1436S)		
BRIP1 (Y1137Y)		
EGFR (T629T)		
BRCA1 (S1613G)		
KRAS (R161R)		
ATM (N1983S)		
BRCA1 (S1040N)		
ERBB2 (P1140A)		
POLD1 (K738N)		
MSH3 (E949R)		
BRCA2 (L1521L)		
EGFR (A613A)		
MSH6 (G39Q)		
BRIP1 (Q879Q)		
TP53 (R213R)		
BRIP1 (S919P)		
CDH1 (A692A)		
EGFR (T903T)		
KRAS (Q61H)		
POLE (A1510A)		
POLE (T1052T)		
POLE (S2084S)		
BARD1 (H506H)		
APC (S1411S)		
TP53 (P72R)		
EGFR (Q787Q)		
TP53 (R175H)		
BARD1 (T351T)		
PMS2 (K541E)		

% of Similarity: 97.92 %

Genes	C2 Organoid	Tissue of origin
MSH2 (L556L)		
PALB2 (T1100T)		
BMPR1A (P2T)		
PALB2 (E559R)		
APC (E260)		
MLH1 (I219V)		
APC (T1556fs)		
PMS2 (V280V)		
PALB2 (G998Q)		
MSH6 (D180D)		
MSH6 (P92P)		
POLD1 (T495T)		
PIK3CA (I391M)		
CTNNA1 (L180L)		
MSH6 (R62R)		
BRCA2 (V2466A)		
KRAS (R162R)		
BARD1 (A40V)		
MSH3 (I78V)		
BRCA2 (V1269V)		
PALB2 (E672Q)		
FANCM (I1460V)		
ATM (S1270C)		
BRCA2 (I3412V)		
BRAF (G643G)		
BRCA2 (V2171V)		
MSH3 (Q949R)		
FANCM (V878L)		
ATM (N1983S)		
EGFR (T629T)		
BRAF (V600E)		
ATM (D1853N)		
MSH3 (A1045T)		
POLE (P1548T)		
BRCA2 (L1521L)		
CTNNA1 (S740S)		
EGFR (T903T)		
FANCM (S175F)		
BRIP1(E879E)		
NBN (D399D)		
BARD1 (P24S)		
CDH1 (A692A)		
BARD1 (H506H)		
TP53 (R273H)		
PMS2 (K541E)		
EGFR (Q787Q)		
EGFR (R521K)		
FANCM (P1812A)		
TP53 (P72R)		

% of Similarity: 91.84 %

Supplementary Fig. S1. (G) List of the gene mutations acquired in the TDOs compared to the tumor of origin (red boxes). The percentage of similarity is also reported. Passage numbers of the organoid lines were: C1: P11; C2: P13; C3: P10; C4: P14; C6: P10.

G

Genes	C3 Organoid	Tissue of origin
PMS2 (K541E)		
PMS2 (P470S)		
PMS2 (S260S)		
TP53 (R273C)		
TP53 (P72R)		
CDKN2A (A148T)		
PALB2 (V932M)		
KRAS (R161R)		
BRCA2 (N372H)		
BRCA2 (K1132K)		
BRCA2 (L1521L)		
BRCA2 (V2171V)		
BRCA2 (S2414S)		
BRCA2 (V2466A)		
RAD51D (R24R)		
ERBB2 (I625V)		
ERBB2 (P1104A)		
BRCA1 (S1613G)		
BRCA1 (S1436S)		
BRCA1 (K1183R)		
BRCA1 (E1038G)		
BRCA1 (P871L)		
BRCA1 (L771L)		
BRCA1 (S694S)		
BRCA1 (D693N)		
FANCM (S175F)		
FANCM (V878L)		
FANCM (I1460V)		
FANCM (I1742V)		
FANCM (P1812A)		
MSH6 (R62R)		
MSH6 (P92P)		
MSH6 (D180D)		
EGFR (N158N)		
EGFR (T903T)		
BRIP1 (Y1137Y)		
BRIP1 (S919P)		
BRIP1 (E879E)		
CDH1 (A692A)		
MSH3 (A55Adel)		
MSH3 (A61Pdup		
MSH3 (P67P69del)		
MSH3 (I79V)		
MSH3 (Q949R)		
MSH3 (A1045T)		
BMPR1A (P2T)		
NBN (P672P)		
NBN (D399D)		
NBN (E185Q)		
NBN (L34L)		
ATM (N1983S)		
APC (Q480)		
APC (D774D)		
APC (I1417fs)		
APC (V1822D)		
POLE (S2084S)		
POLE (A1510A)		
POLE (T1052T)		
CTNNA1 (A179V)		
PIK3CA (Q546R)		

% of Similarity: 100 %

Genes	C4 Organoid	Tissue of origin
KRAS (R161R)		
KRAS (G12S)		
BRCA2 (K1132L)		
BRCA2 (L1521L)		
BRCA2 (T1915M)		
BRCA2 (V2171V)		
BRCA2 (S2414)		
BRCA2 (V2466A)		
MSH6 (R62R)		
MSH6 (P92P)		
MSH6 (D180D)		
MSH6 (R1034Q)		
SMAD4 (D351N)		
POLD1 (T495T)		
EGFR (T903T)		
EGFR (D994D)		
BRIP1 (Y1137Y)		
BRIP1 (S919P)		
BRIP1 (E879E)		
CDH1 (T379M)		
CDH1 (A692A)		
MSH3 (A55A60del)		
MSH3 (P67P69del)		
MSH3 (I79V)		
MSH3 (Q949R)		
BMPR1A (P2T)		
ATM (N1983S)		
APC (Y486Y)		
APC (A545A)		
APC (S835fs)		
APC (L1488)		
APC (T1493T)		
APC (G1678G)		
APC (S1756S)		
APC (V1822D)		
APC (P1960P)		
POLE (S2084S)		
BRAF (G643G)		
BARD1 (V507M)		

% of Similarity: 87.18 %

Supplementary Fig. S1. (G) List of the gene mutations acquired in the TDOs compared to the tumor of origin (red boxes). The percentage of similarity is also reported. Passage numbers of the organoid lines were: C1: P11; C2: P13; C3: P10; C4: P14; C6: P10.

G

Genes	C6 organoid	Tissue of origin
PMS2 (P470S)		
PMS2 (S260S)		
TP53 (G245V)		
TP53 (P72A)		
CDKN2A (A148T)		
KRAS (R161R)		
KRAS (G12A)		
BRCA2 (N372H)		
BRCA2 (K1132K)		
BRCA2 (L1521L)		
BRCA2 (T1915M)		
BRCA2 (V2171V)		
BRCA2 (S2414S)		
BRCA2 (V2466A)		
RAD51D (R145C)		
MLH1 (I219V)		
ERBB2 (P1140A)		
BRCA1 (S1613G)		
BRCA1 (S1436S)		
BRCA1 (K1183R)		
BRCA1 (E1038G)		
BRCA1 (P871L)		
BRCA1 (L771L)		
BRCA1 (S694S)		
MUTYH (S515F)		
MUTYH (Q338H)		
MSH6 (R62R)		
MSH6 (P92P)		
MSH6 (D180D)		
MSH6 (Y214Y)		
SMAD4 (D315H)		
EGFR (N158N)		
EGFR (R521K)		
EGFR (Q787Q)		
EGFR (R836R)		
EGFR (T903T)		
BRIP1 (Y1137Y)		
BRIP1 (S919P)		
BRIP1 (E879E)		
CDH1 (A692A)		
MSH3 (A55A60del)		
MSH3 (P67P69del)		
MSH3 (I79V)		
MSH3 (Q949R)		
MSH3 (A1045T)		
BMPR1A (P2T)		
ATM (D1853N)		
ATM (N1983S)		
APC (Y486Y)		
APC (A545A)		
APC (L1129S)		
APC (Q1406fs)		
APC (T1493T)		
APC (G1678G)		
APC (S1756S)		
APC (V1822D)		
APC (P1960P)		
POLE (S2084S)		
POLE (A1510A)		
POLE (N1396S)		
POLE (T1052T)		
CTNNA1 (S740S)		
BRAF (G643G)		
BARD1 (H506H)		
BARD1 (T351T)		

% of Similarity: 93.85 %

Supplementary Fig. S1. (G) List of the gene mutations acquired in the TDOs compared to the tumor of origin (red boxes). The percentage of similarity is also reported. Passage numbers of the organoid lines were: C1: P11; C2: P13; C3: P10; C4: P14; C6: P10.

Supplementary Figure S2

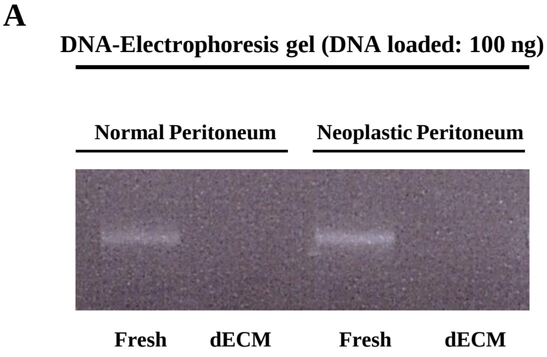

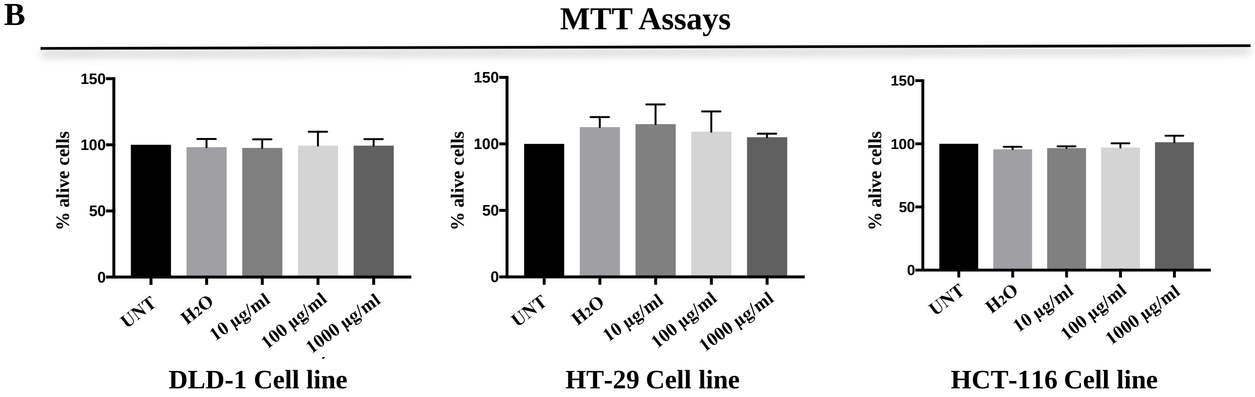

Supplementary Fig. S2. (A) To confirm the efficacy of the decellularization treatment, the loss of DNA content was assessed. DNA was extracted from 20 mg of non-decellularized or decellularized ECM and quantified with the Nanodrop instrument. Ten µl of DNA from PM or dECM were loaded onto a 1% agarose gel and the images were acquired using Gel Doc (Biorad). The results showed the absence of DNA in dECM samples, indicating the success of the decellularization procedure. The experiments were performed in triplicate. **(B)** Assessment of whether the components and procedures of the decellularization protocol can influence cell viability. CRC-derived DLD-1, HT-29 and HCT-116 cell lines were obtained from the American Type Culture Collection (ATCC, Rockville, MD, USA) and were authenticated by the Cell Culture Facility at the FIRC Institute of Molecular Oncology (IFOM, Milan, Italy) with the StemElite ID system (Promega). Cells were routinely tested for mycoplasma and cultured following the recommended ATCC's protocols. The 3D-dECMs were placed at − 80 °C overnight, lyophilized using freeze-drier cycles following standard procedures, and then added to the culture media of the three cell lines. During the experiments, three different concentrations of lyophilized 3D-dECMs were tested and cell viability was evaluated after 72 hours of growth by MTT (3-(4, 5-dimethylthiazol-2-yl)-2, 5-diphenyltetrazolium bromide) assay (Sigma Aldrich), following the manufacturer's instructions. The results showed no differences in cell viability between cells cultured with or without lyophilized ECM, highlighting that the decellularization procedure is safe for cell growth. The experiments were performed in duplicate.

Supplementary Figure S3

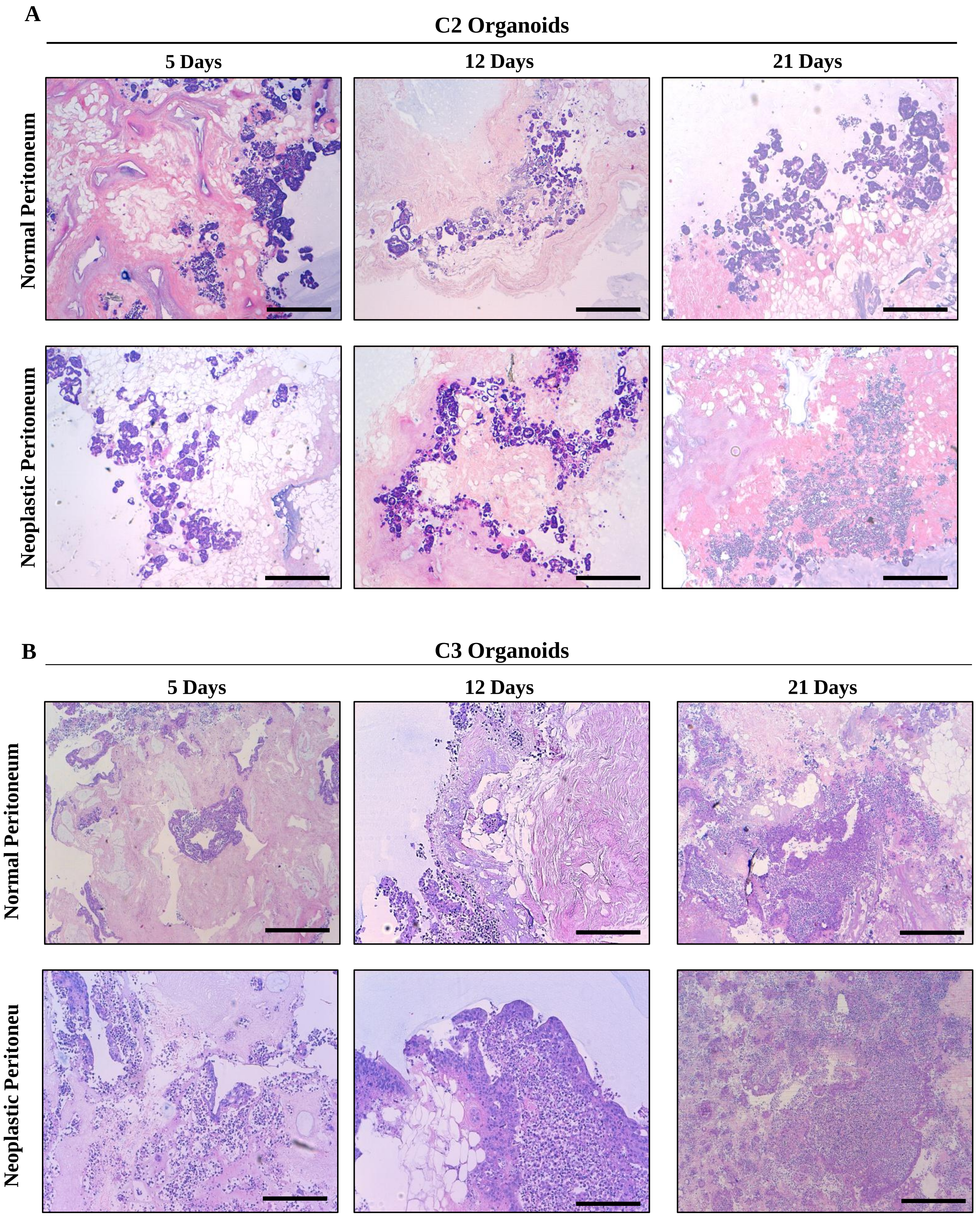

Supplementary Fig. S3. (A) H&E staining of decellularized matrices derived from normal (top) or neoplastic (bottom) peritoneum repopulated with PM-derived organoids (C2) at three different time points as indicated. Scale bar: 100 µm. The repopulation experiments were performed in triplicate. **(B)** H&E staining of decellularized matrices derived from normal (top) or neoplastic (bottom) peritoneum repopulated with PM-derived organoids (C3) on day 12. Scale bar: 100 µm. The repopulation experiments were performed in triplicate.

C **C2 Organoid**

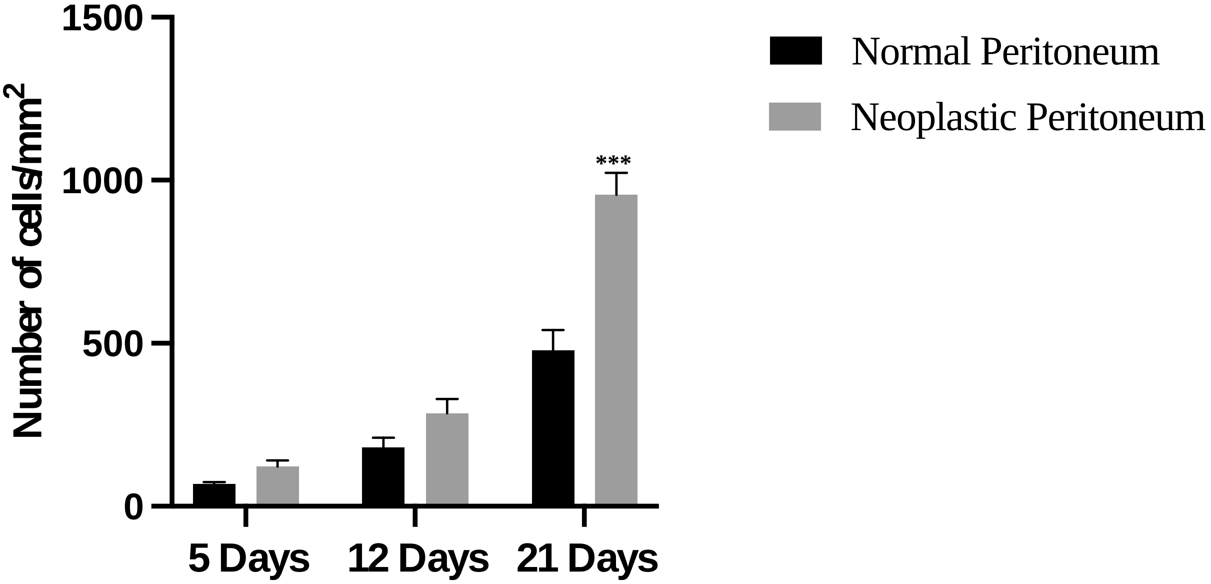

D **C3 Organoid**

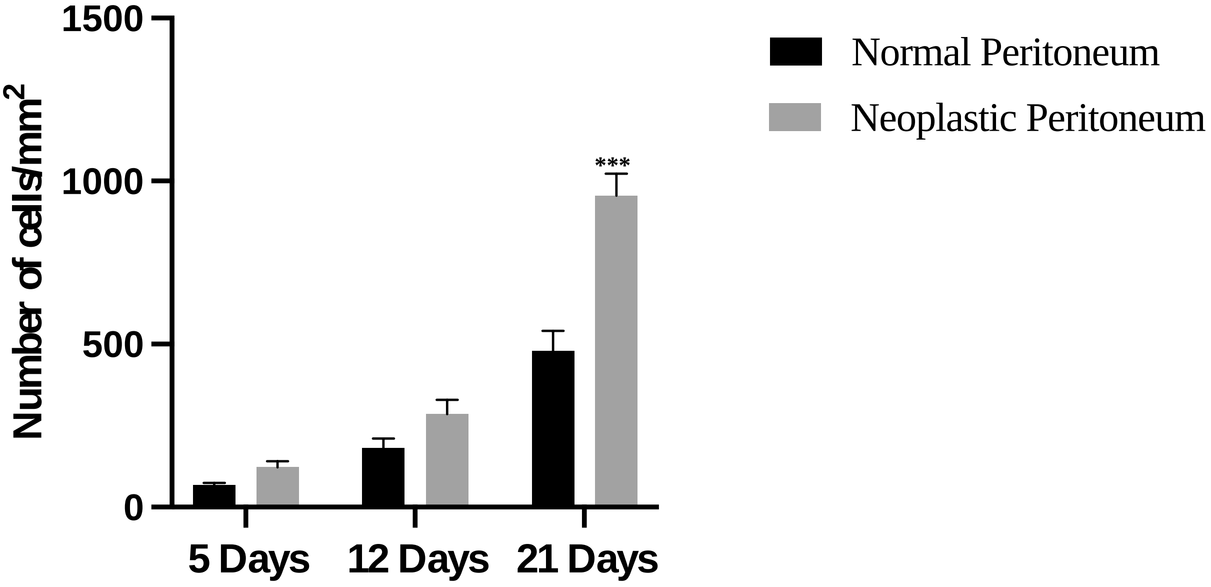

Supplementary Fig. S3. (C) Number of C2 PM-organoid derived cells grown onto normal and neoplastic-derived 3D-dECM per mm^2 after 5, 12 and 21 days. Three fields per experiments were counted using Qpath software. Data are presented as median and SD. One-way ANOVA (****p*<0.001). **(D)** Number of C3 PM-organoid derived cells grown onto normal and neoplastic-derived 3D-dECM per mm^2 after 5, 12 and 21 days. Three fields per experiments were counted using Qpath software. Data are presented as median and SD for surgical specimens of three patients. One-way ANOVA (****p*<0.001). The repopulation experiments were performed in triplicate.

E

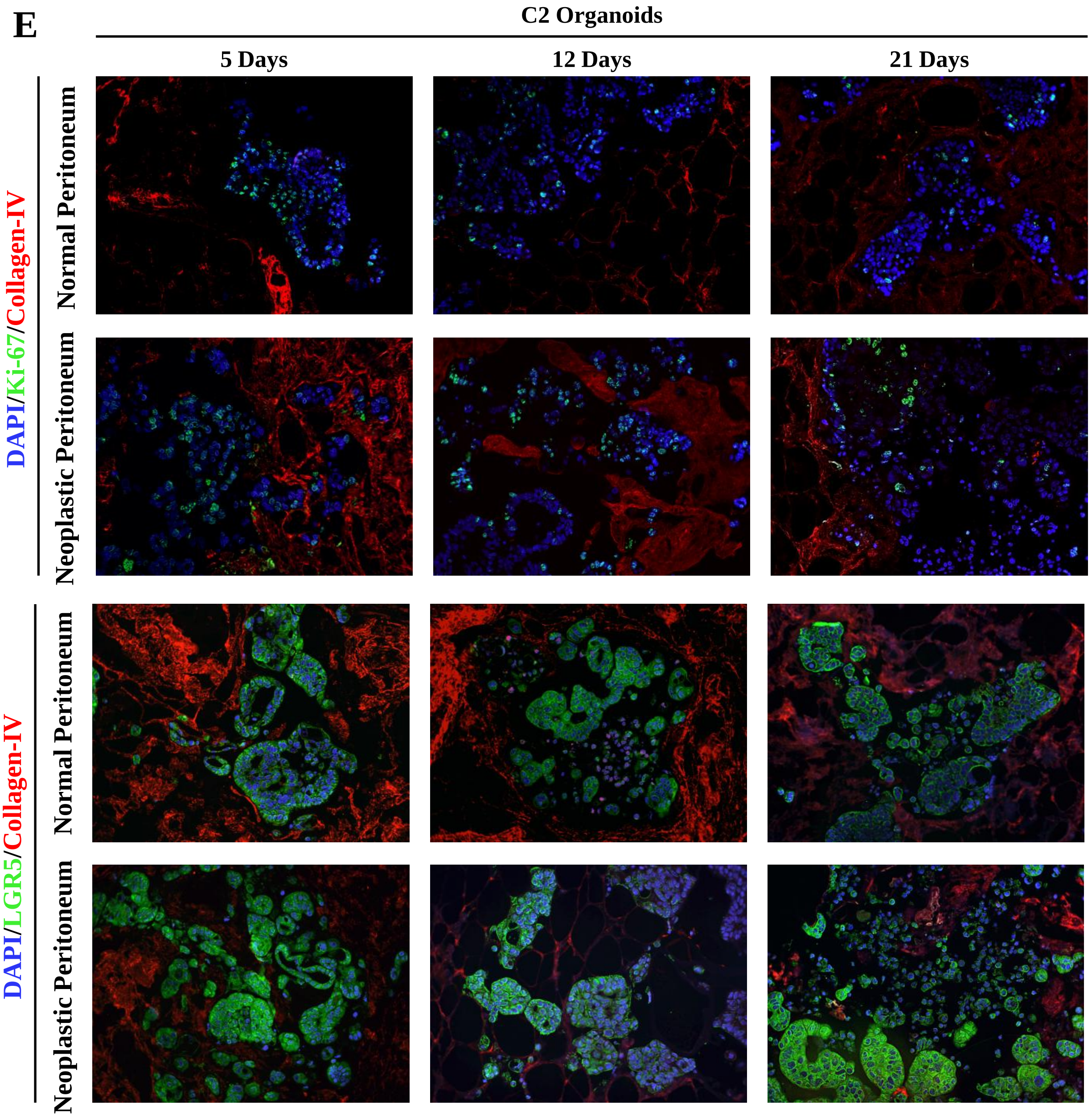

F

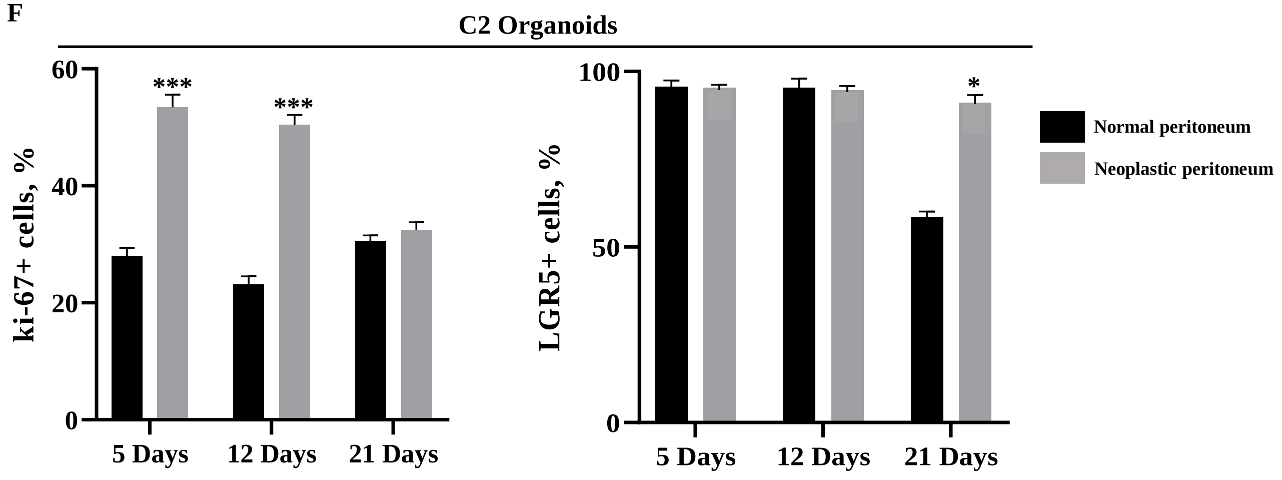

Supplementary Fig. S3. (E) Top panel: IF analysis of 3D-dECMs derived from normal (top) and neoplastic (bottom) peritoneum repopulated with PM-derived organoids (C2) at different time points as indicated, using Ki-67 (green) and collagen IV (red) antibodies. Scale bar: 50 µm. The experiments were performed in triplicate. **Bottom panel:** IF analysis of 3D-dECMs derived from normal (top) and neoplastic (bottom) peritoneum repopulated with PM-derived organoids (C2) at different time points as indicated, using LGR5 (green) and collagen IV (red) antibodies. The samples were counterstained with DAPI (blue) Scale bar: 50 µm. The experiments were performed in triplicate. **(F) Left panel:** proliferation rate of PM-derived organoids, measured as the percentage of Ki-67^{+} cells present in fields devoid of dead cells. Five fields per experiment (40X magnification) were counted. Data are presented as median and SD. One-way ANOVA (***p<0.001). **Right panel:** amount of stem cells in PM-derived organoids, measured as the percentage of LGR5^{+} cells present in fields devoid of dead cells. Five fields per experiment (40X magnification) were counted. Data are presented as median and SD. One-way ANOVA (*p<0.05). The experiments were performed in triplicate.

G

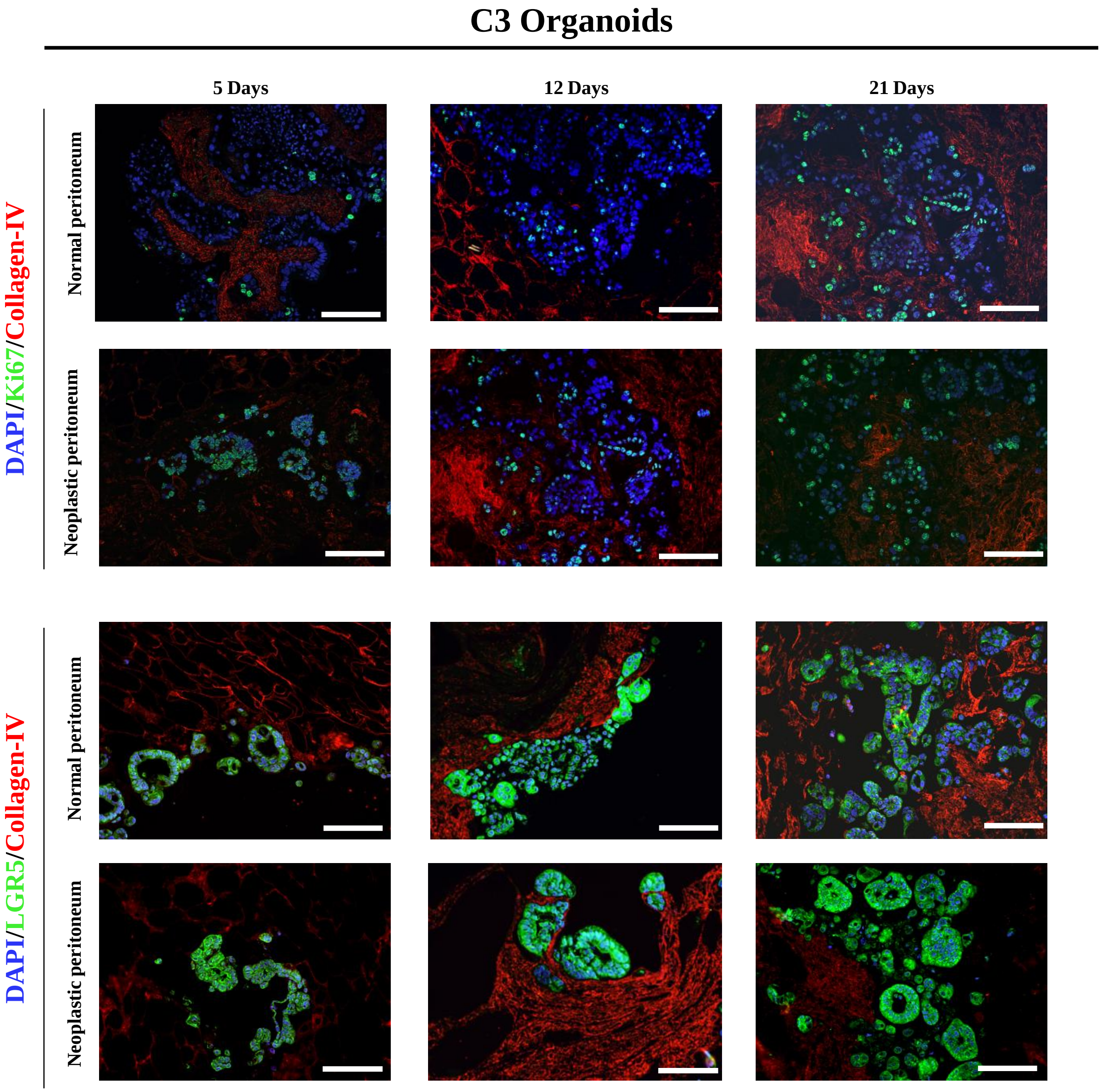

H

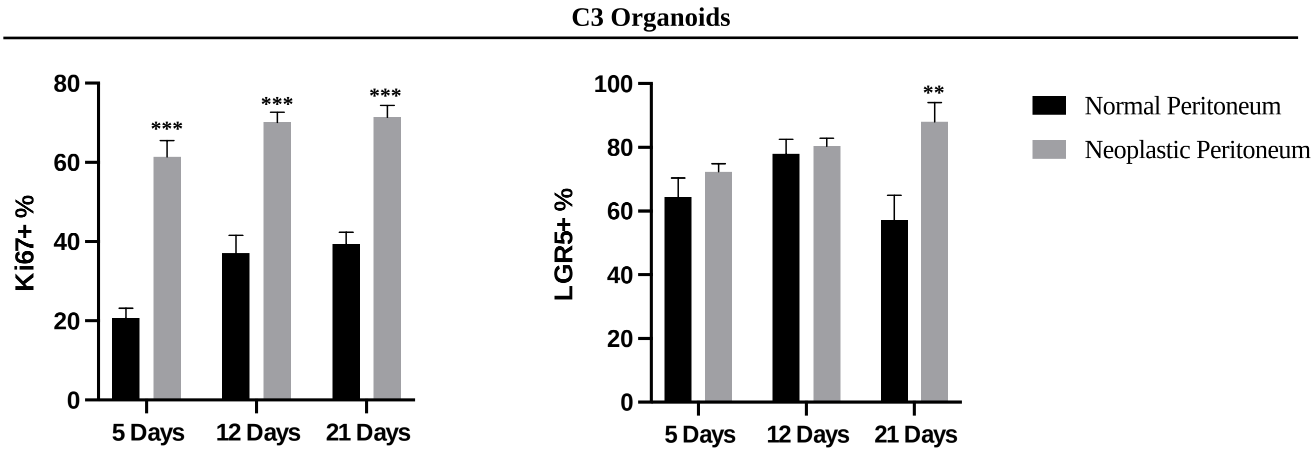

Supplementary Fig. S3. (G) Top panel: IF analysis of 3D decellularized matrices derived from normal (top) and neoplastic (bottom) peritoneum repopulated with PM-derived organoids (C3), using Ki-67 (green) and collagen IV (red) (left panel). **Bottom panel:** LGR5 (green) and collagen IV (red) (right panel) antibodies. The samples were counterstained with DAPI (blue). Scale bar: 50 µm. The experiments were performed in triplicate. **(H) Left panel:** proliferation rate of PM-derived organoids measured as the percentage of Ki-67$^+$ cells present in fields devoid of dead cells. Five fields per experiment (40X magnification) were counted. Data are presented as median and SD. Student's *t-test* (***p<0.01). **Right panel:** percentage of stem cells in PM-derived organoids, measured as the percentage of LGR5$^+$ cells present in fields devoid of dead cells. Five fields per experiment (40X magnification) were counted. Data are presented as median and SD. Student's *t-test* (*p<0.05). The experiments were performed in triplicate.

Supplementary Figure S4

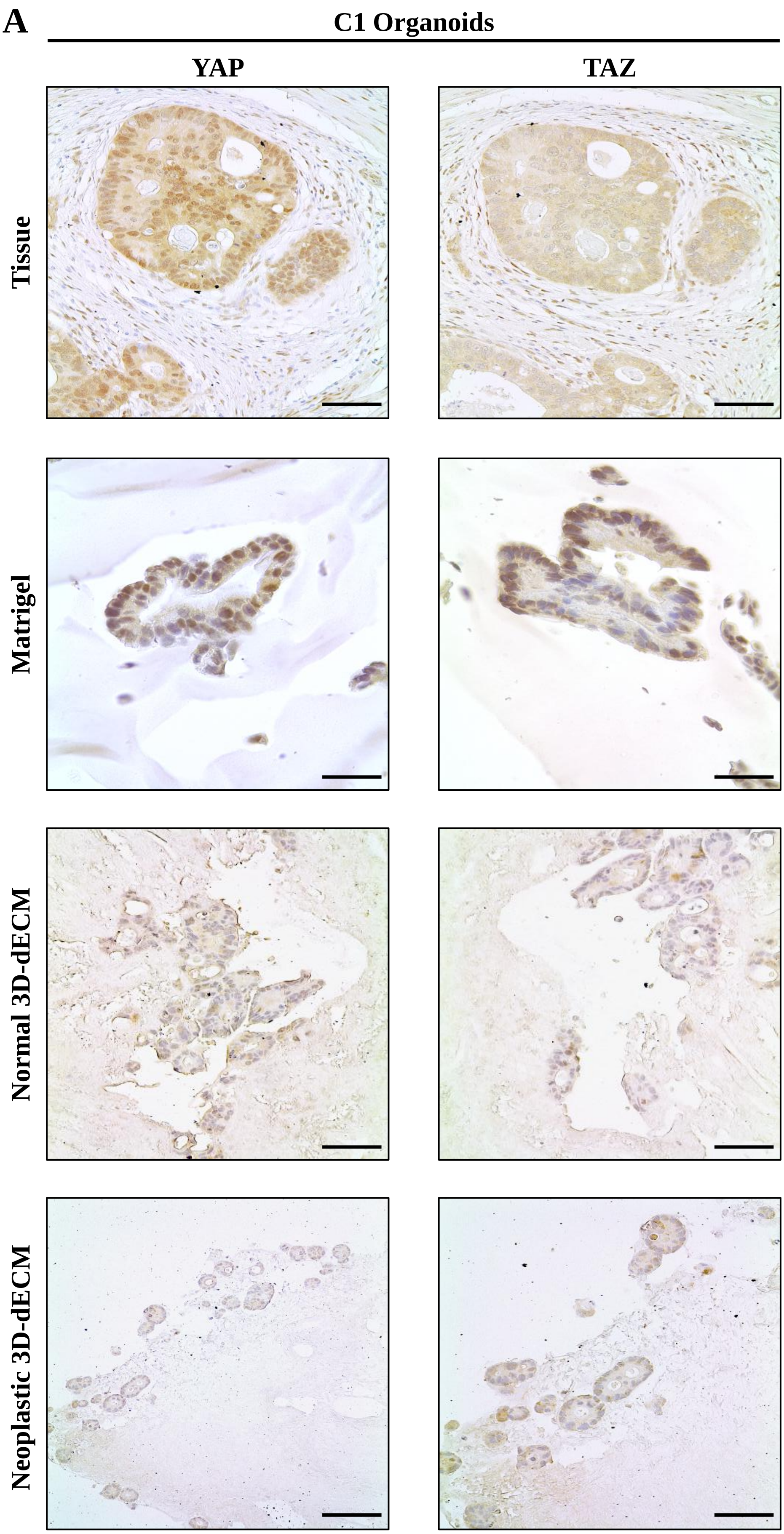

Supplementary Fig. S4. (A) Comparative immunohistochemical images of PM-derived organoids (C1) grown on different substrates (Matrigel, Normal 3D-dECM and Neoplastic 3D-dECM) and their corresponding tumor of origin. Expression of YAP and TAZ proteins was analysed. Scale bar: 100 µM.

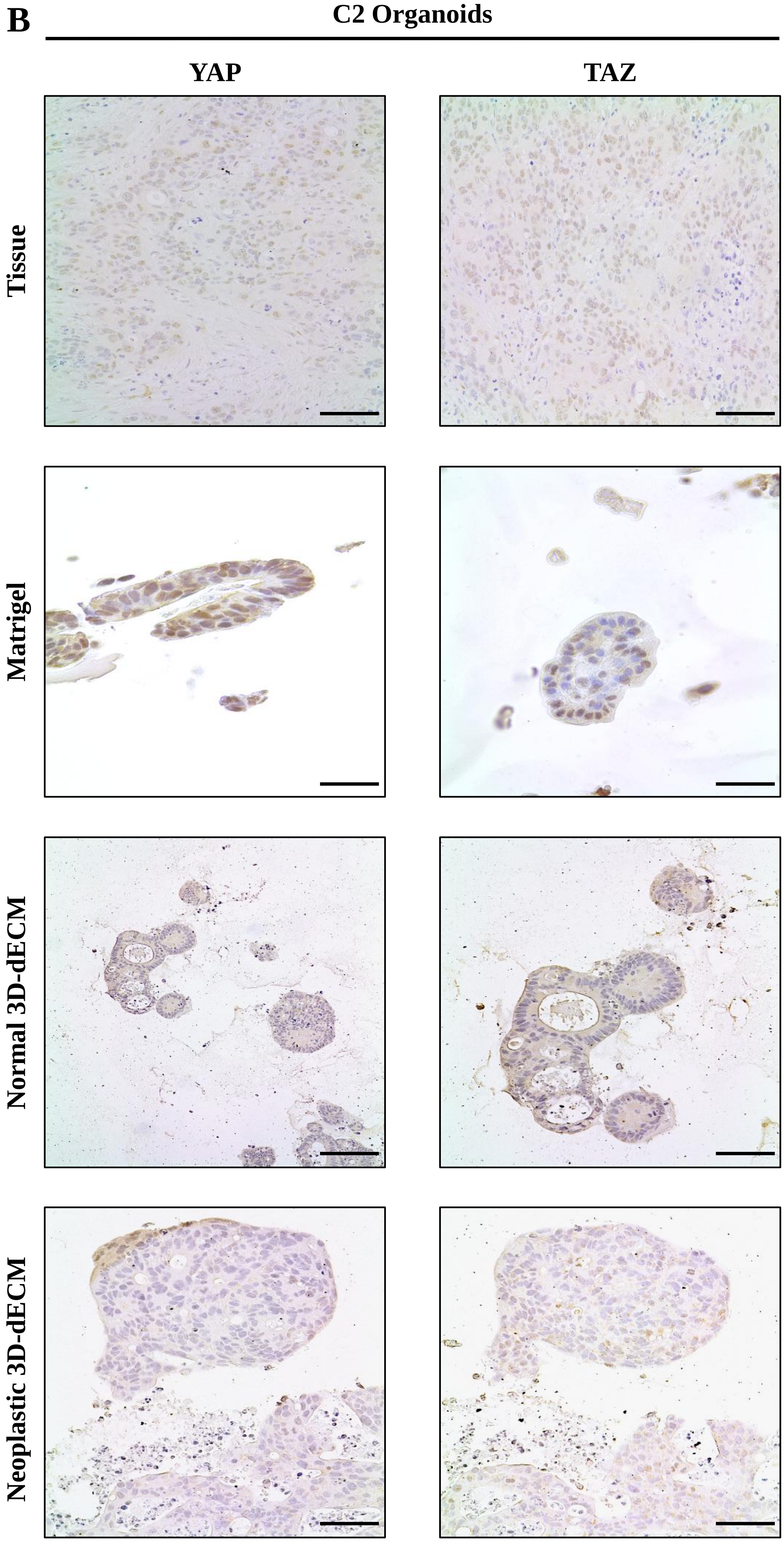

Supplementary Fig. S4. (B) Comparative immunohistochemical images of PM-derived organoids (C2) grown on different substrates (Matrigel, Normal 3D-dECM and Neoplastic 3D-dECM) and their corresponding tumor of origin. Expression of YAP and TAZ proteins was analyzed. Scale bar: 100 μM.

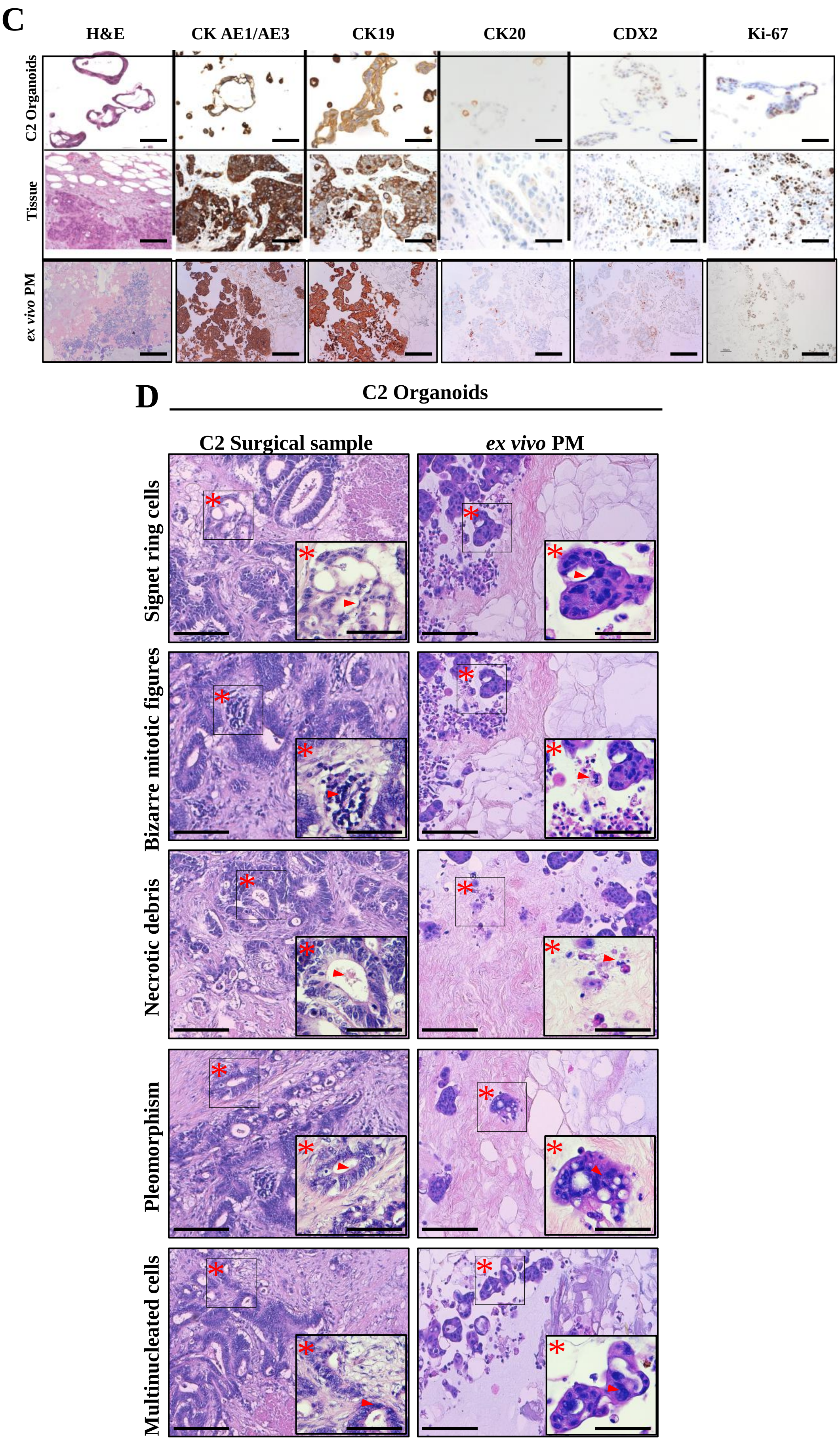

Supplementary Fig. S4. (C) Comparative histological and immunohistochemical analysis of C2 PM-derived organoids Vs their corresponding tumor of origin and the *ex vivo* engineered PM lesion. Samples were analyzed for the expression of the CRC-specific markers as indicated. Scale bar: 50 μM. Images in the first two lanes were previously published [11]. **(D)** Histological analysis of PM and neoplastic-derived 3D-dECM repopulated with C2 PM-derived organoids. The *ex vivo* engineered PM lesions present histological features that are typical of PMs of gastrointestinal origin. Asterisks and arrows indicated the main morphological features. Scale bar: 20 μm.

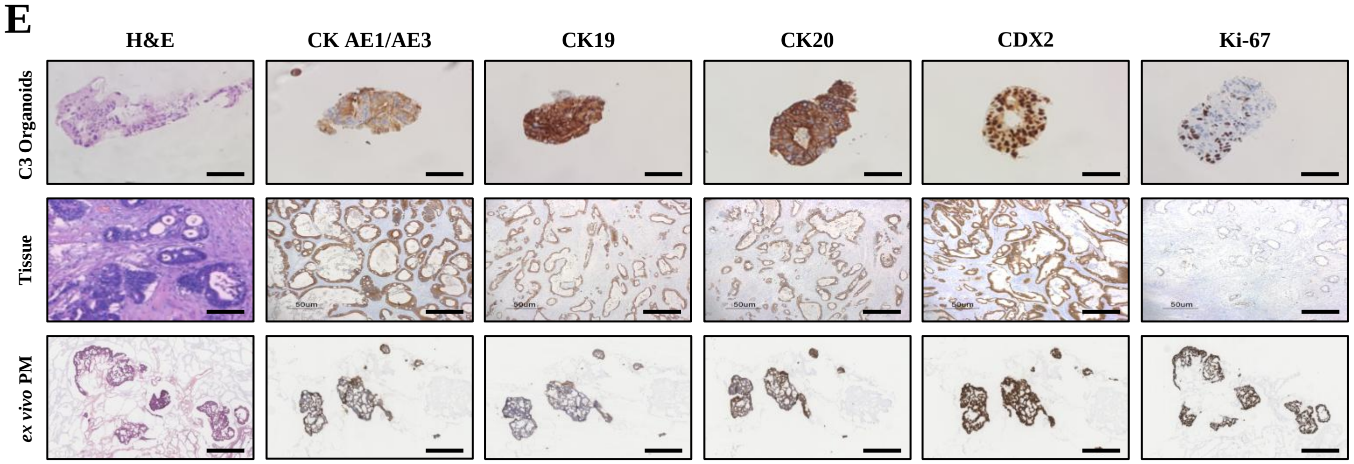

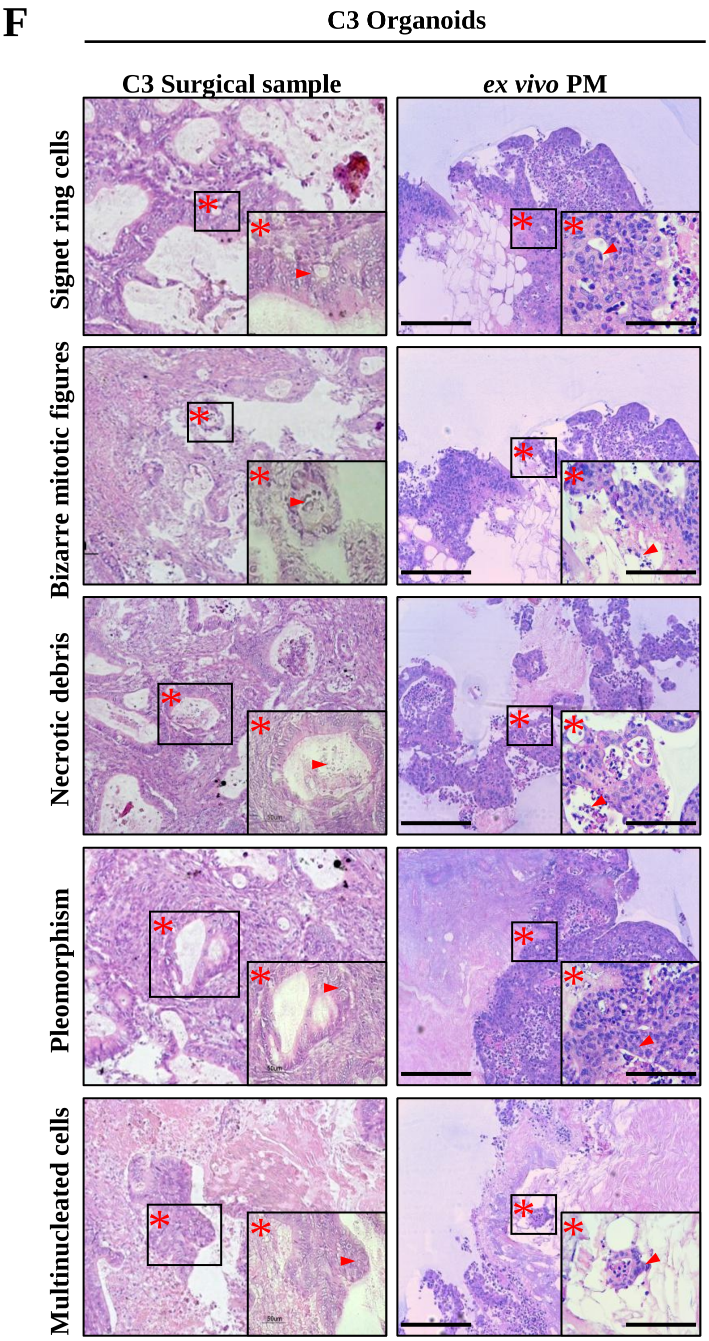

Supplementary Fig. S4. (E) Comparative histological and immunohistochemical analysis of C3 PM-derived organoids Vs their corresponding tumor of origin and the *ex vivo* engineered PM lesion. Samples were analyzed for the expression of the CRC-specific markers as indicated. Scale bar: 50 µm. **(F)** Histological analysis of peritoneal metastasis and neoplastic-derived 3D-dECMS repopulated with C3 PM-derived organoids. The *ex vivo* engineered PM lesions present histological features that are typical of PMs of gastrointestinal origin. Asterisks and arrows indicated the main morphological features. Scale bar: 20 µm.

G

C2 Organoids

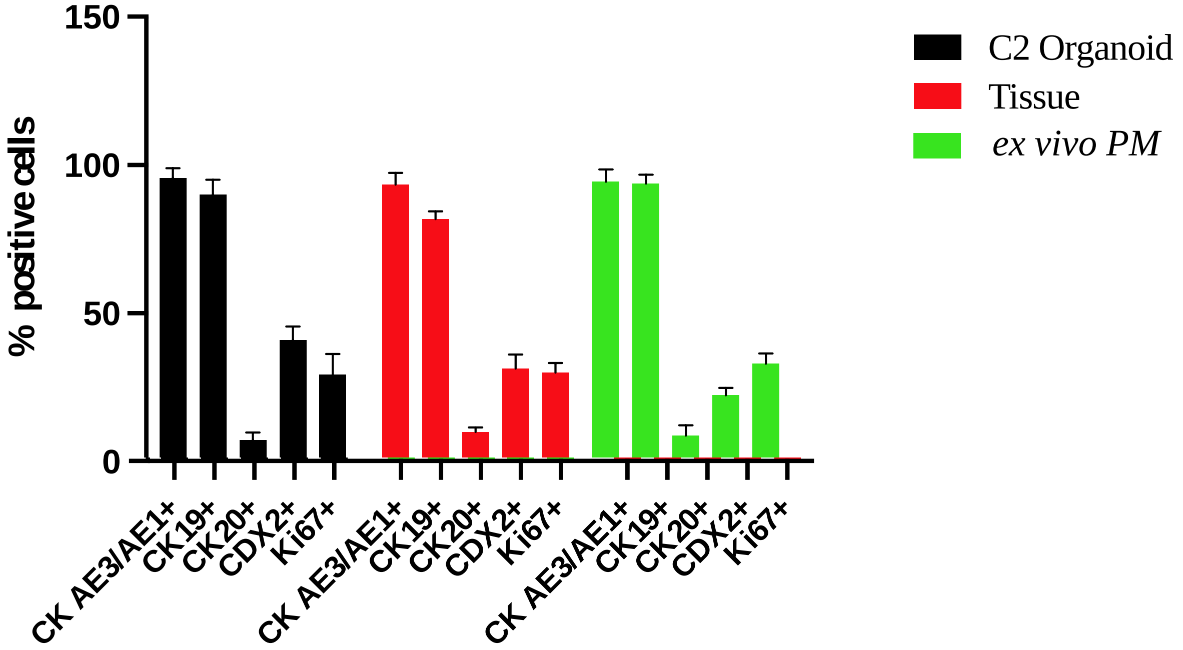

H

C3 Organoids

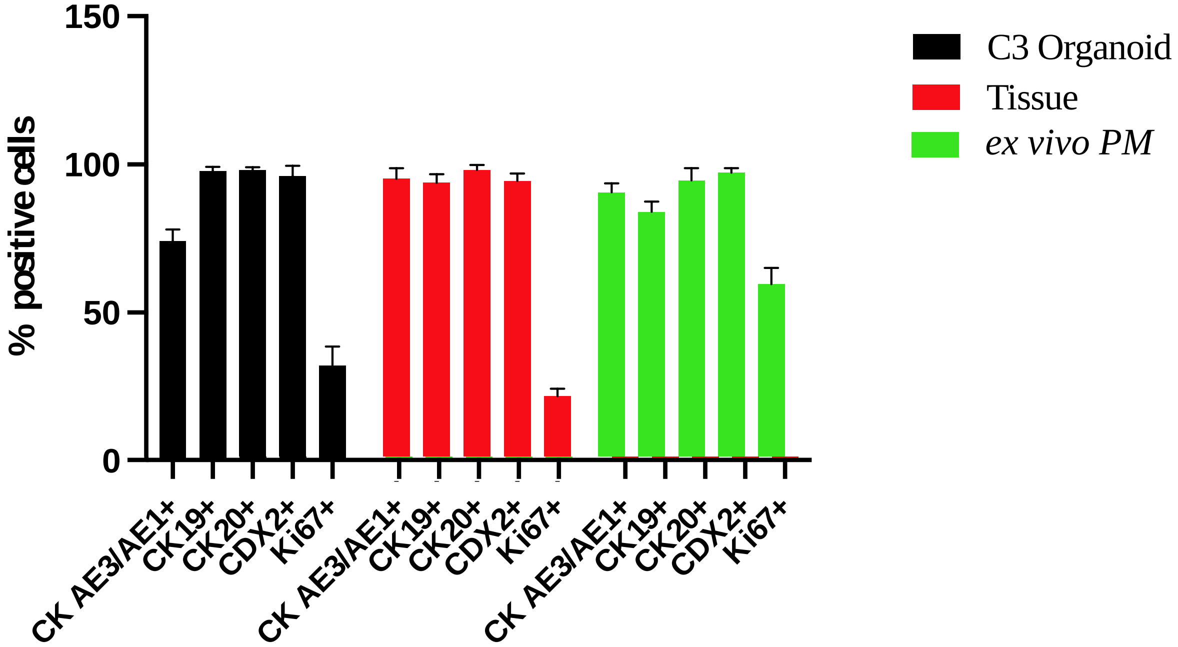

Supplementary Fig. S4. (G) Quantitative counts of the percentage of CK AE/AE3, CK19, CK20, CDX2 and Ki-67 positive cells in C2 PM-derived organoids Vs their corresponding tumor of origin and the *ex vivo* engineered PM lesion Three fields per experiments were counted using Qpath software. Data are presented as median and SD. One-way ANOVA did not show differences between the two groups. **(H)** Quantitative counts of the percentage of CK AE/AE3, CK19, CK20, CDX2 and Ki-67 positive cells in C3 PM-derived organoids Vs their corresponding tumor of origin and the *ex vivo* PM engineered lesion. Three fields per experiments were counted using Qpath software. Data are presented as median and SD. One-way ANOVA did not show differences between the two groups.

Supplementary Figure S5

A

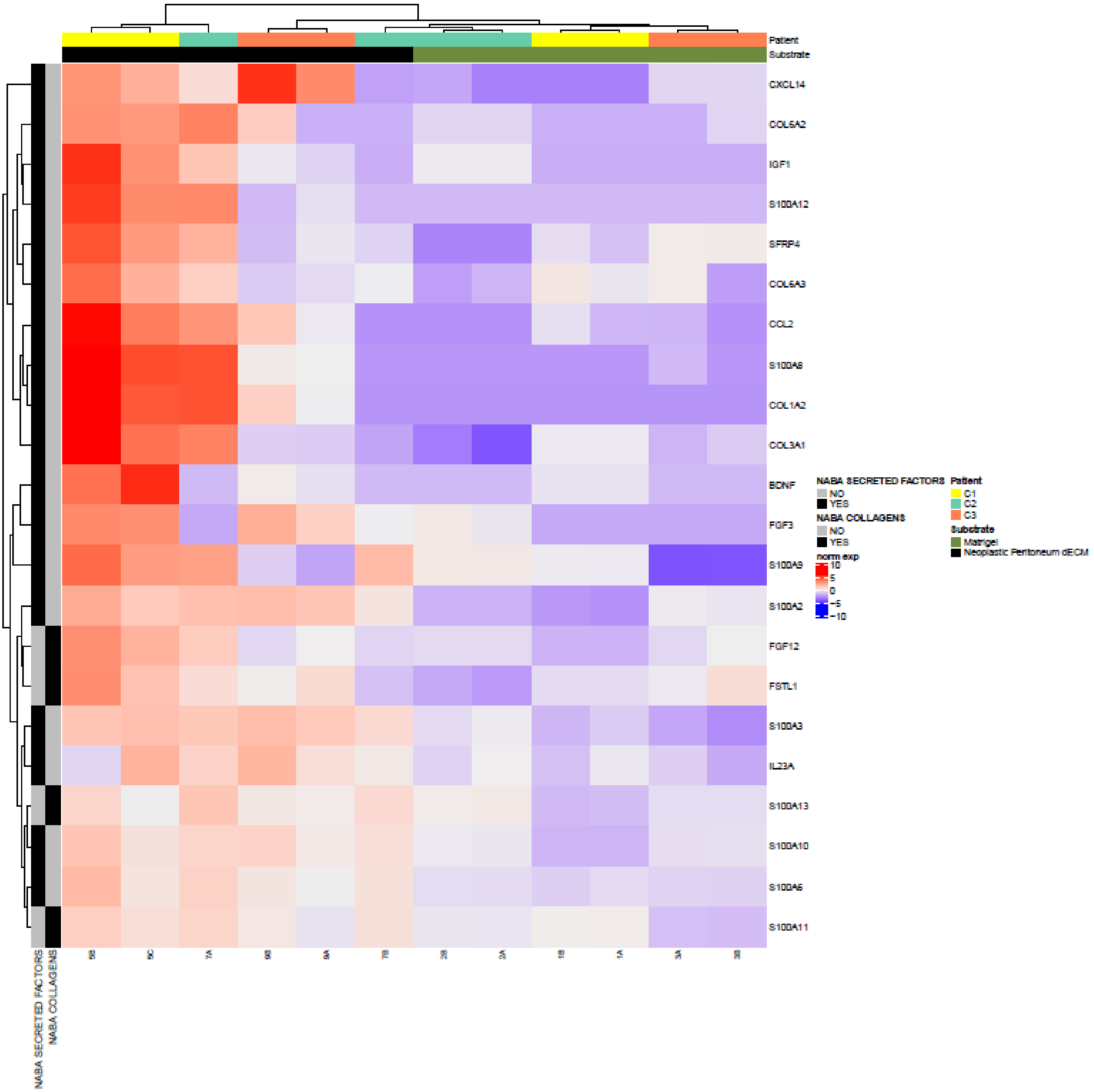

Supplementary Fig. S5. (A) Unsupervised hierarchical clustering of organoids based on the expression of the top DEGs between organoids grown on neoplastic 3D-dECM and in Matrigel, and present in Naba Secreted Factors or Naba Collagen categories.

B

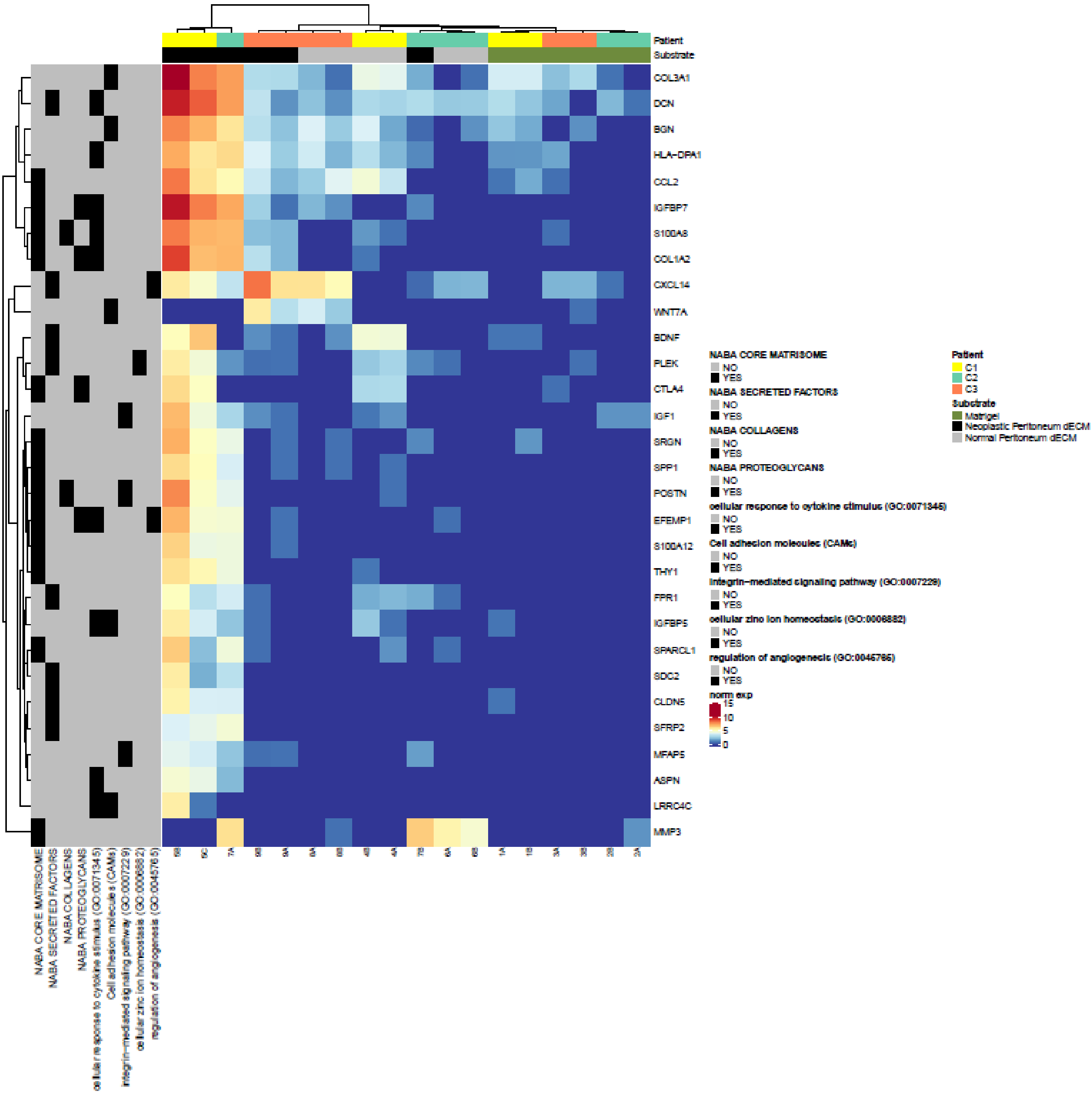

Supplementary Fig. S5. (B) Unsupervised hierarchical clustering of organoids based on the expression of the top DEGs between organoids grown on neoplastic 3D-dECM, normal 3D-dECM and in Matrigel, and present in Naba Core Matrisome, Naba Secreted Factors, Naba Collagen categories, Naba Proteoglycan categories, cellular response to cytokine stimulus, cell adhesion molecules, integrin mediated signaling pathway, cellular zinc ion homeostasis, and regulation of angiogenesis categories.

C

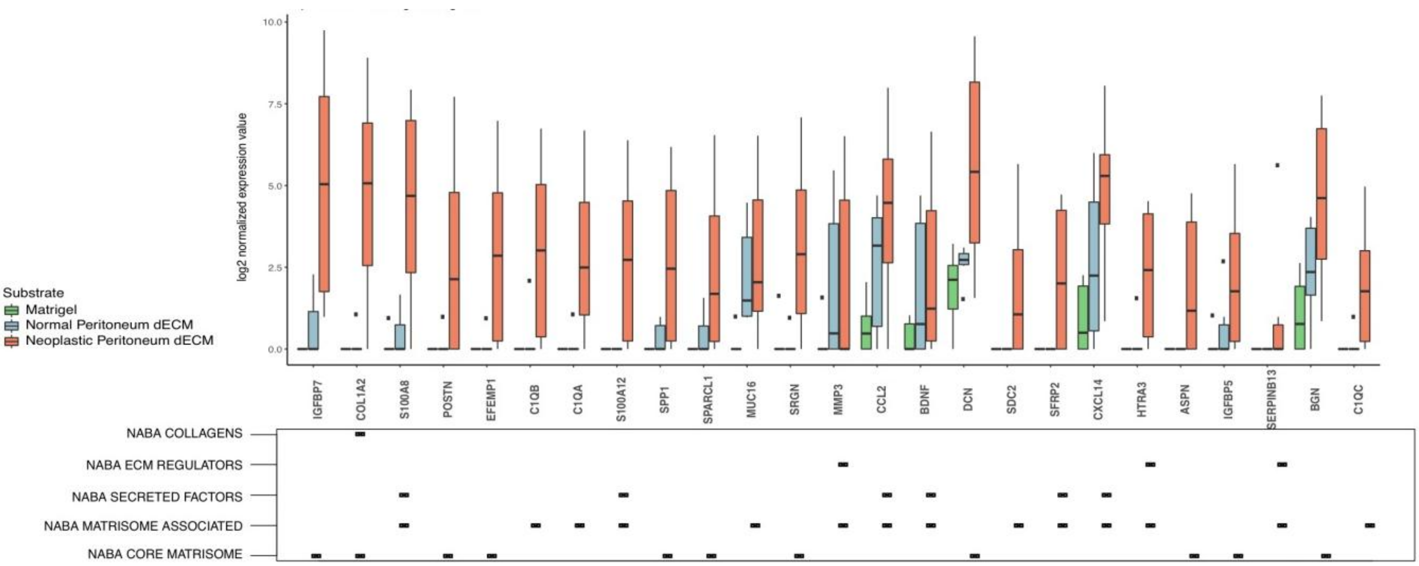

D

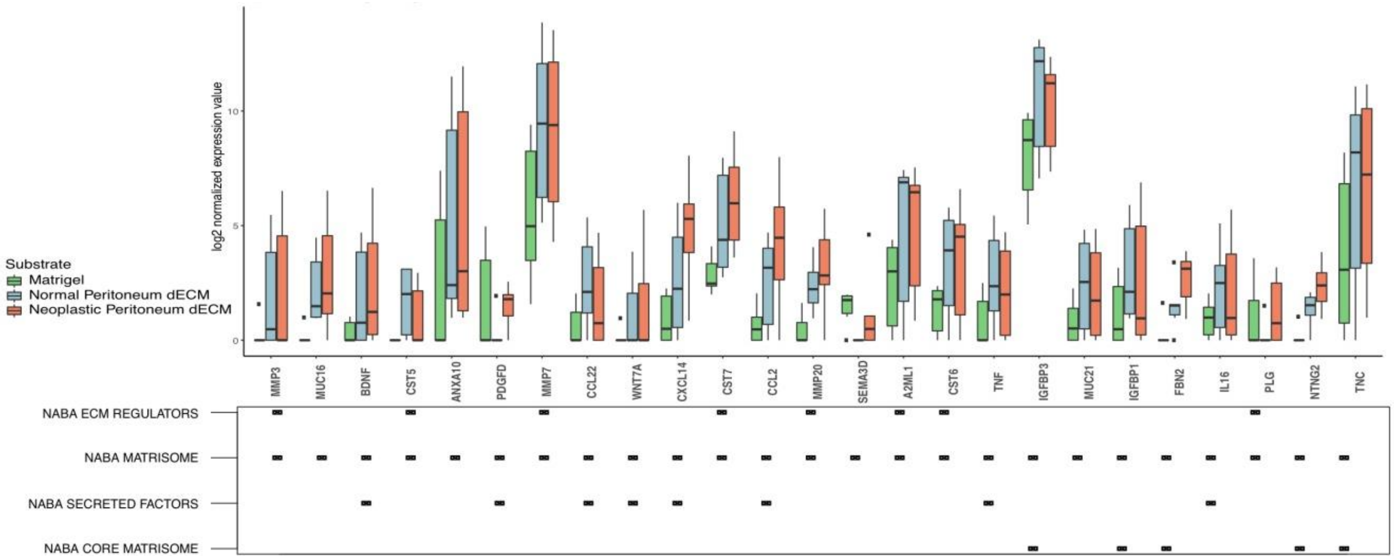

Supplementary Fig. S5. (C) Box plot showing the expression in organoids grown on neoplastic 3D-dECM and in Matrigel, of DEGs selected based on their involvement in the indicated processes of the Naba Matrisome geneset. DEGs expression in organoids grown on normal 3D-dECM is also shown. Median and interquartile range are displayed as horizontal lines. Black squares in the bottom panel indicate which category the genes belong to. **(D)** Box plot showing the expression, in organoids grown on normal 3D-dECM and in Matrigel, of DEGs selected based on their involvement in the indicated processes of the Naba Matrisome geneset. DEGs expression in organoids grown on neoplastic 3D-dECM is also shown. Median and interquartile range are displayed as horizontal lines. Black squares in the bottom panel indicate which category the genes belong to.

E

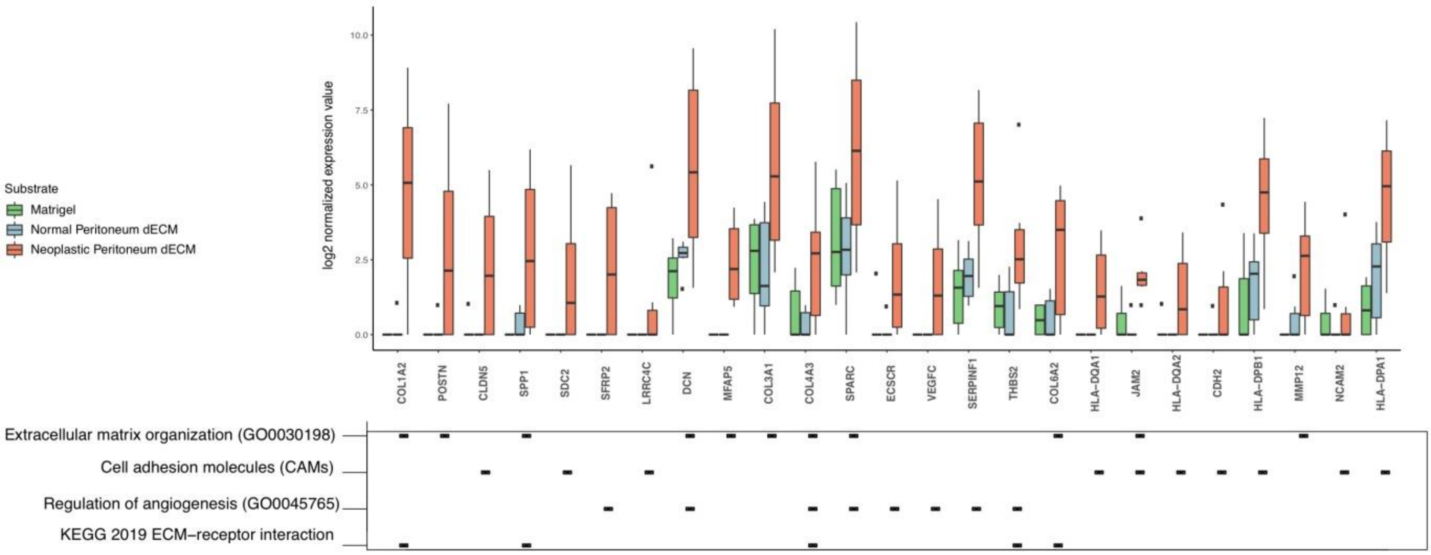

F

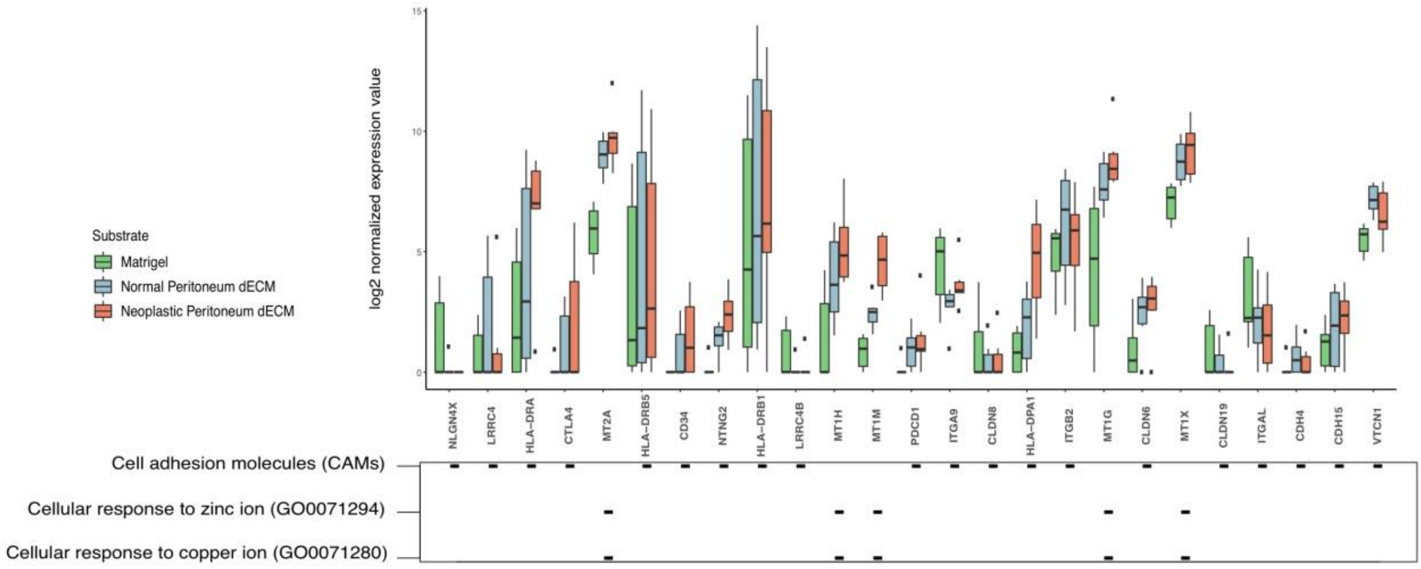

Supplementary Fig. S5. (E) Box plot showing the expression in organoids grown on normal 3D-dECMs and in Matrigel, of DEGs selected based on their involvement in the indicated processes of GO Biological Process, KEGG and Reactome databases. DEGs expression in organoids grown on neoplastic 3D-dECM is also shown. Median and interquartile range are displayed as horizontal lines. Black squares in the bottom panel indicate which category the genes belong to. **(F)** Box plot showing the expression levels of genes selected based on their involvement in the indicated processes. The expression levels of the selected genes were compared in organoids grown in Matrigel, on neoplastic 3D-dECM and on normal 3D-dECM. Median and interquartile range are displayed as horizontal lines. Black squares in the bottom panel indicate which category the genes belong to.

G

	Collection	ID	Term	Adjusted *p-value*	Leading Edge genes (%)	# Genes
Neoplastic 3D-dECM Vs Matrigel	GO BP	GO:0071345	Cellular response to cytokine stimulus	0.003365	27.1	413
	GO BP	GO:0045765	Regulation of angiogenesis	0.003365	19.3	171
	GO BP	GO:0006882	Cellular zinc ion homeostasis	0.003365	46.7	30
	GO BP	GO:0071280	Cellular response to copper ion	0.003365	55.0	20
	GO BP	GO:0007229	Integrin-mediated signaling pathway	0.011230	19.6	56
	KEGG		Wnt signaling pathway	0.022987	20.5	151
Normal 3D-dECM Vs Matrigel	GO BP	GO:0071280	Cellular response to copper ion	0.006015	55.0	20
	GO BP	GO:0071294	Cellular response to zinc ion	0.006015	64.7	17
	KEGG		Cell adhesion molecules (CAMs)	0.006015	32.8	128
Neoplastic 3D-dECM Vs Normal 3D-dECM	KEGG		ECM-receptor interaction	0.004499	25.0	80
	GO BP	GO:0045765	Regulation of angiogenesis	0.009176	15.2	171
	KEGG		Cell adhesion molecules (CAMs)	0.020511	22.7	132
	Reactome	R-HSA-5660526	Response to metal ions Homo sapiens	0.040965	81.8	11
	GO BP	GO:0030198	Extracellular matrix organization	0.002740	25.3	221

H

	Collection	Term	Adjusted *p-value*	Leading Edge genes (%)	# Genes
Neoplastic 3D-dECM Vs Matrigel	NABA MATRISOME	Naba Matrisome Associated	0.003365	38.2	663
	NABA MATRISOME	Naba Core Matrisome	0.003365	36.1	266
	NABA MATRISOME	Naba ECM Regulators	0.003365	30.6	222
	NABA MATRISOME	Naba Secreted Factors	0.003365	45.1	288
	NABA MATRISOME	Naba Collagens	0.004307	31.2	44
Normal 3D-dECM Vs Matrigel	NABA MATRISOME	Naba ECM Regulators	0.0060150	39.2	217
	NABA MATRISOME	Naba Core Matrisome	0.006015	22.7	256
	NABA MATRISOME	Naba Secreted Factors	0.006015	34.7	274
	NABA MATRISOME	Naba Matrisome	0.006024	32.9	897
Neoplastic 3D-dECM Vs Normal 3D-dECM	NABA MATRISOME	Naba Core Matrisome	0.002740	26.3	266
	NABA MATRISOME	Naba Secreted Factors	0.002740	39.2	291
	NABA MATRISOME	Naba ECM Glycoproteins	0.002740	21.9	192
	NABA MATRISOME	Naba Collagens	0.002740	40.9	44
	NABA MATRISOME	Naba Proteoglycans	0.002740	53.3	30
	NABA MATRISOME	Naba ECM Regulators	0.024908	28.2	16

Supplementary Fig. S5. (G) Selected categories enriched in DEGs between organoids grown on neoplastic 3D-dECM, normal 3D-dECM and in Matrigel involved in relevant biological processes according to GO Biological Process, KEGG and Reactome databases. **(H)** Selected Matrisome categories enriched in DEGs between organoids grown on neoplastic 3D-dECM, normal 3D-dECM and in Matrigel involved in relevant biological processes according to Naba Maatrisome dataset.

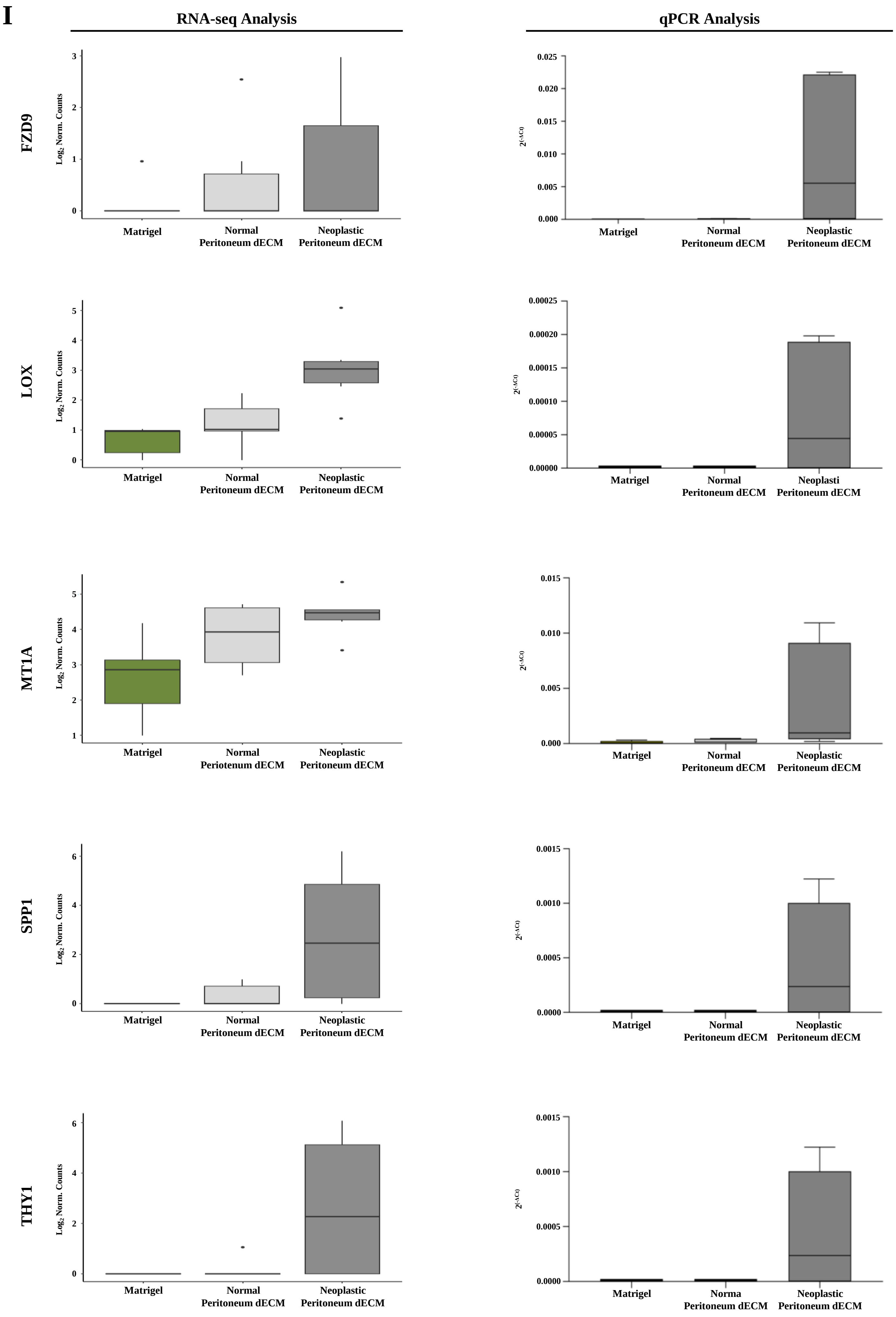
I
RNA-seq Analysis
qPCR Analysis
FZD9
LOX
MT1A
SPP1
THY1
Log2 Norm. Counts
2(-ΔCt)
Matrigel
Normal
Peritoneum dECM
Neoplastic
Peritoneum dECM
Periotenum dECM
Neoplasti
Norma
0.025
0.020
0.015
0.010
0.005
0.000
0.00025
0.00020
0.00015
0.00010
0.00005
0.00000
0.0015
0.0010
0.0005
0.0000

L

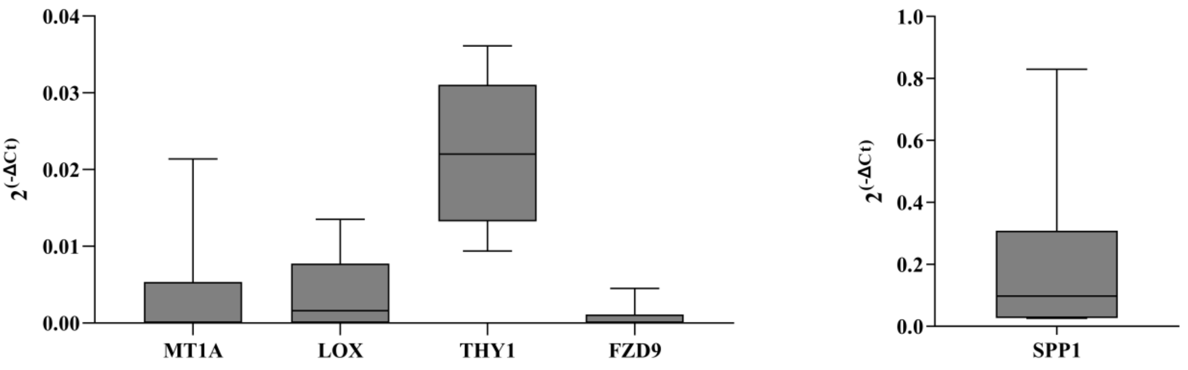

Supplementary Fig. S5. (I) Expression levels of MT1A, LOX, THY1, FZD9 and SPP1 in C1, C2 and C3 grown into Matrigel and on normal and neoplastic-derived 3D-dECMs (left panel). Values are calculated as log_2 of the total normal counts. The horizontal line inside the box indicates the median and the whiskers indicate the extreme measured values.

Expression levels of MT1A, LOX, THY1, FZD9 and SPP1 genes on organoid cultures grown in Matrigel and on normal or neoplastic peritoneal 3D-dECMs (right panel). Values are calculated as $2(^{-\Delta Ct})$ and normalized to GAPDH. The horizontal line inside the box indicates the median and the whiskers indicate the extreme measured values. **(L)** Expression levels of MT1A, LOX, THY1, FZD9 and SPP1 genes on FFPE tissues from PM-derived organoids. Values are calculated as $2(^{-\Delta Ct})$ and normalized to GAPDH. The horizontal line inside the box indicates the median and the whiskers indicate the extreme measured values.

Supplementary Figure S6

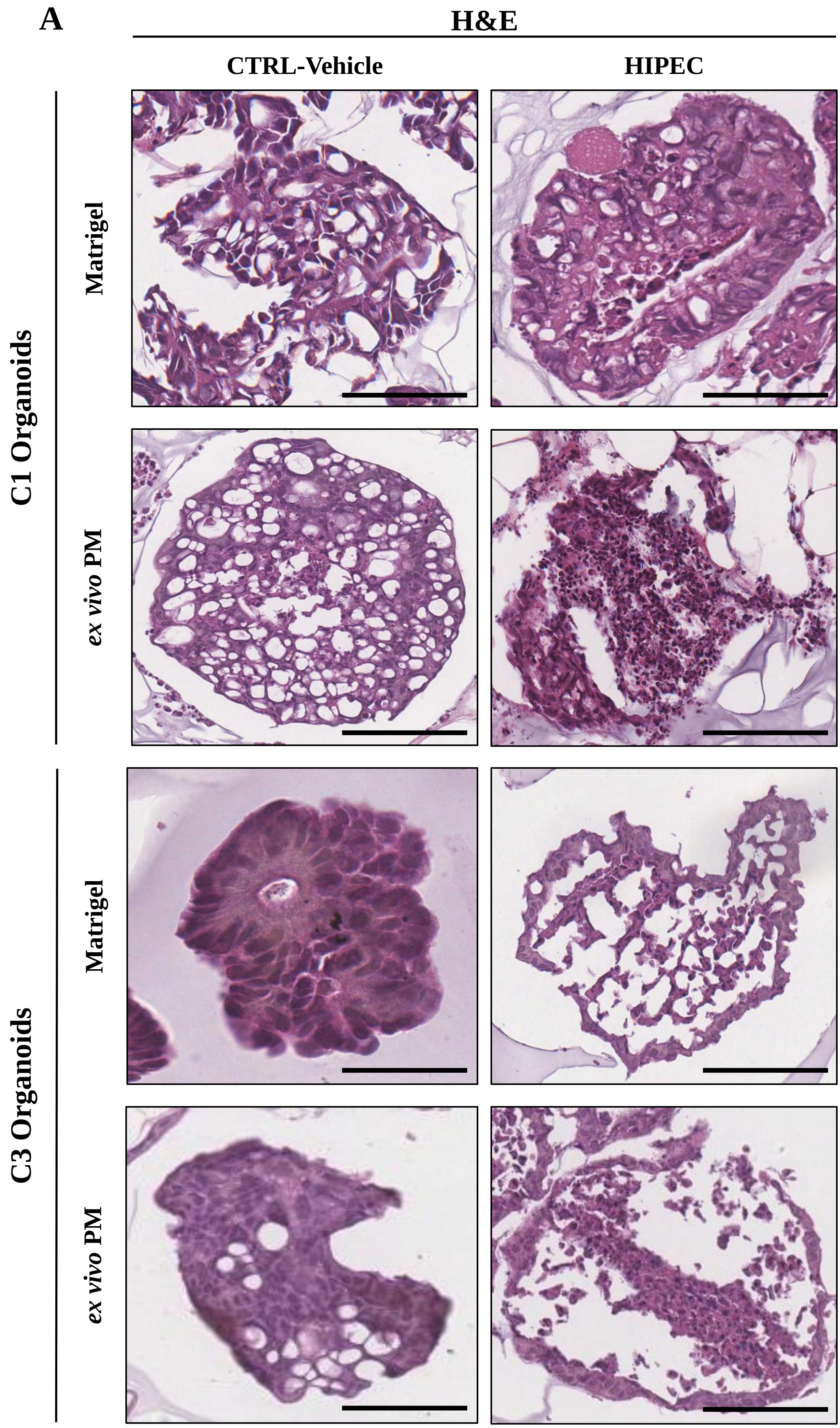

Supplementary Fig. S6. (A) H&E staining of C1 and C3 organoids cultured in Matrigel and on neoplastic-derived peritoneal 3D-dECMs after *in vitro* HIPEC treatments. Scale bar: 50 µm.

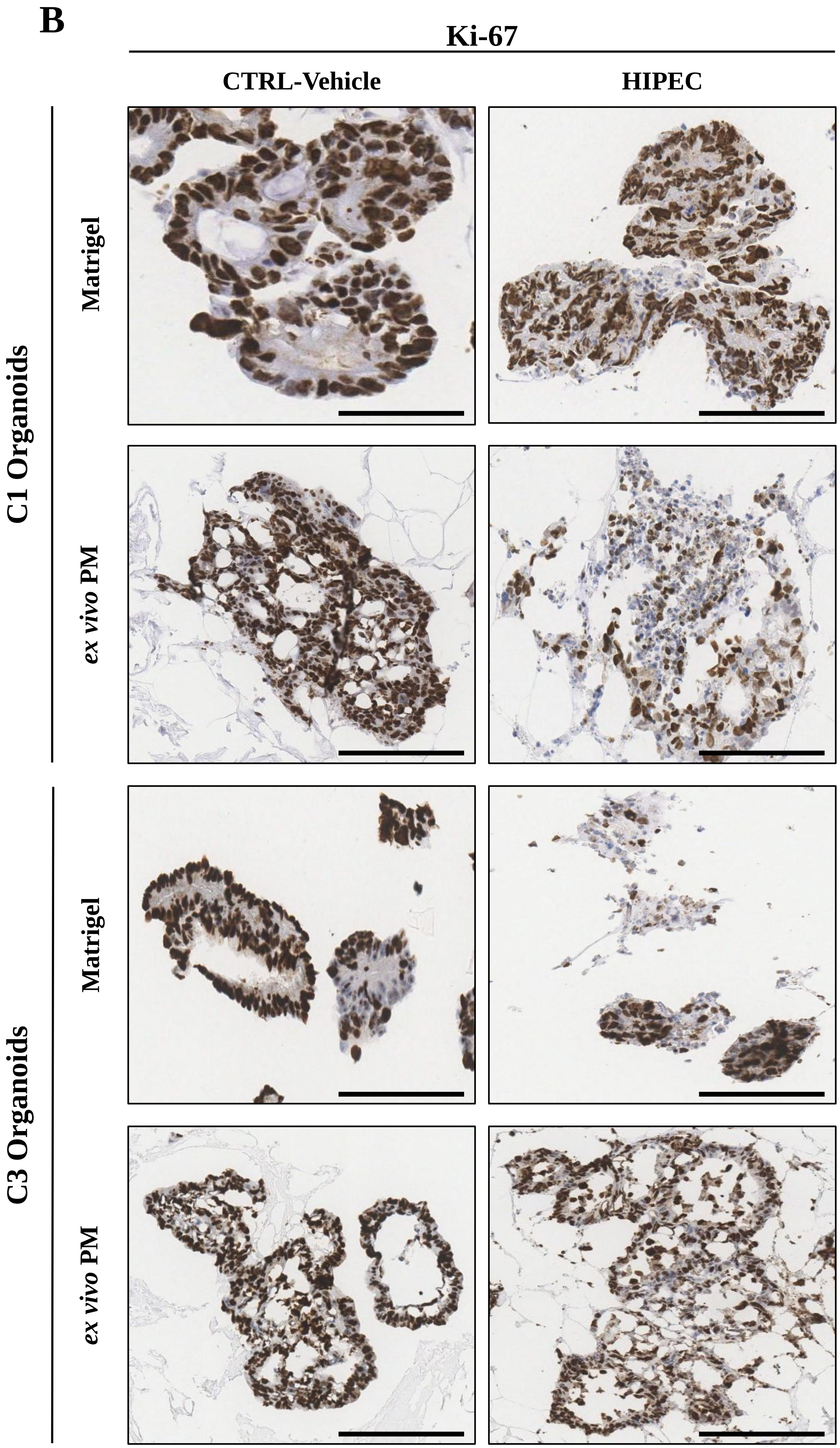

Supplementary Fig. S6. (B) Ki-67 immunostaining of C1 and C3 organoids cultured in Matrigel and on neoplastic-derived peritoneal 3D-dECMs after *in vitro* HIPEC treatments. Scale bar: 50 µm.

C

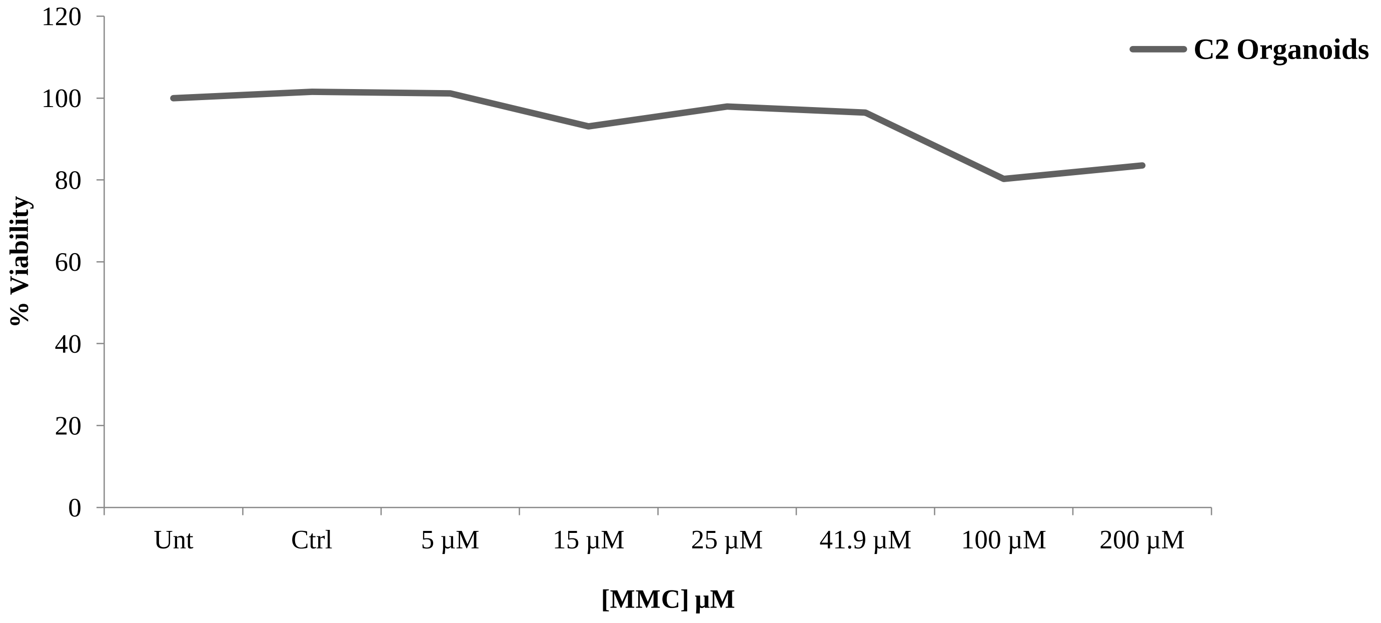

D

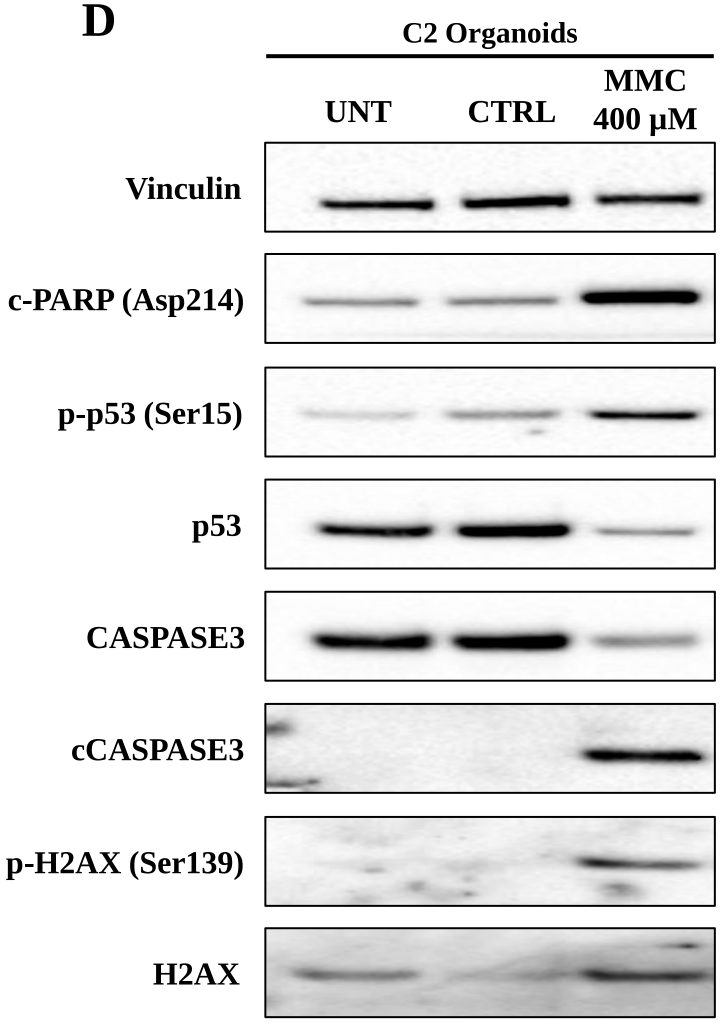

Supplementary Fig. S6. (C) Dose response curve of C2 PM-derived organoids cultured in Matrigel and treated with MMC at different concentrations at 42.5 °C for 1 h. **(D)** Immunoblots of cPARP, p-p53, p53, CASPASE3, cCASPASE3, p-H2AX and H2AX in C2 PM-derived organoids treated with MMC 400 µM. Vinculin was used as loading control.

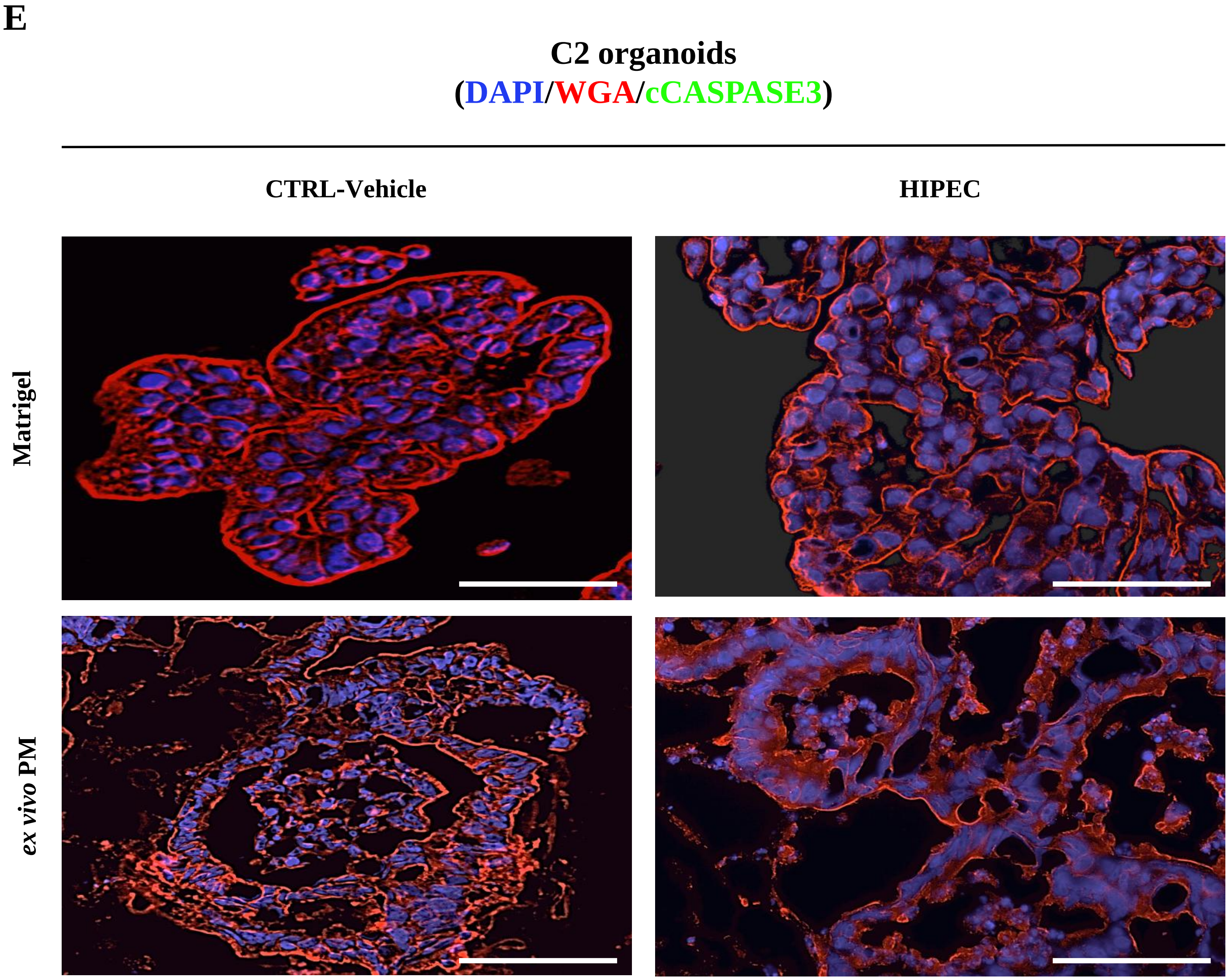

Supplementary Fig. S6 (E) IF analysis of C2 PM-derived organoids cultured in Matrigel and on neoplastic-derived peritoneal 3D-dECMs after *in vitro* HIPEC treatments, using cCASPASE3 (green) antibody. The samples were counterstained with WGA (red) and DAPI (blue). Scale bar: 50 µm.

F

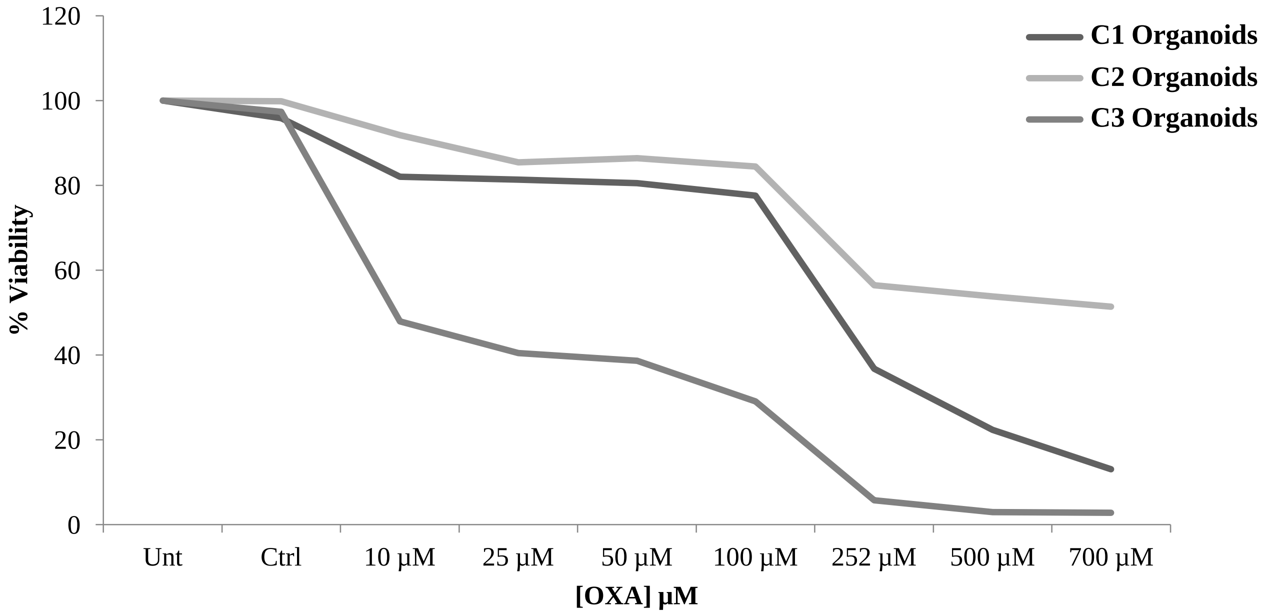

G

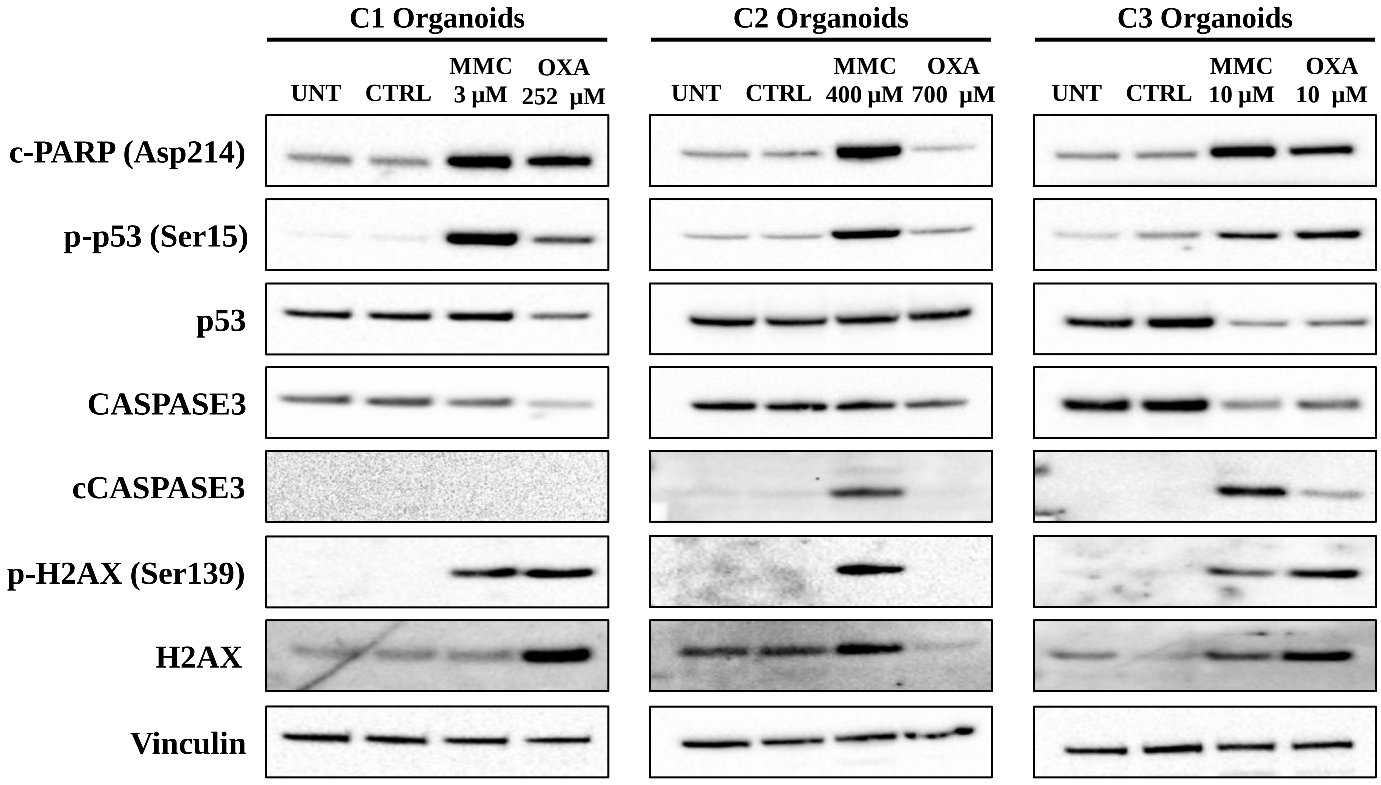

Supplementary Fig. S6. (**F**) Dose response curve of C1, C2 and C3 PM-derived organoids cultured in Matrigel and treated with OXA at different concentrations at 42.5 °C for 90 min. **(G)** Immunoblots of cPARP, p-p53, p53, CASPASE3, cCASPASE3, p-H2AX and H2AX in C1, C2 and C3 PM-derived organoids treated with MMC and OXA at the respective IC_{50} concentrations. Vinculin was used as loading control. Ki-67 immunostaining of C2 PM-derived organoids cultured in Matrigel and on neoplastic-derived peritoneal 3D-dECMs after *in vitro* HIPEC treatments.

H

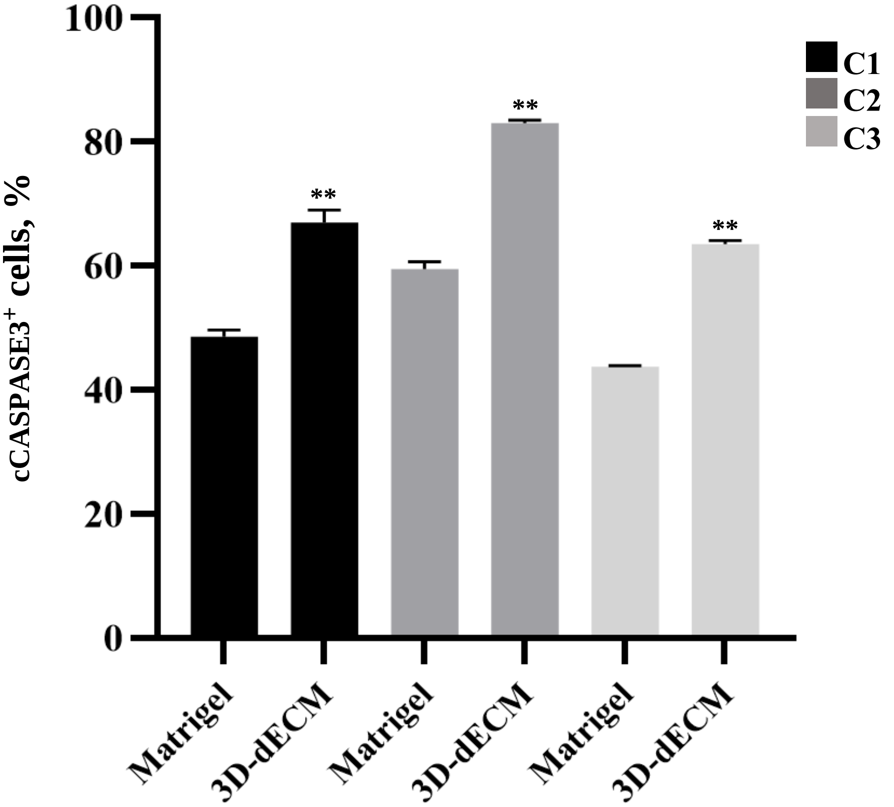

Supplementary Fig. S6. (**H**) Percentage of apoptotic TDO treated with OXA, measured as the percentage of cCASPASE-3+ cells present in selected fields. Five fields per experiment (40X magnification) were counted. Data are presented as median and SD . One-way ANOVA (**p<0.01).
